# Global burden and trends of firearm violence in 204 countries/territories from 1990 to 2019

**DOI:** 10.3389/fpubh.2022.966507

**Published:** 2022-08-30

**Authors:** Zejin Ou, Yixian Ren, Danping Duan, Shihao Tang, Shaofang Zhu, Kexin Feng, Jinwei Zhang, Jiabin Liang, Yiwei Su, Yuxia Zhang, Jiaxin Cui, Yuquan Chen, Xueqiong Zhou, Chen Mao, Zhi Wang

**Affiliations:** ^1^Key Laboratory of Occupational Environment and Health, Guangzhou Twelfth People's Hospital, Guangzhou, China; ^2^Department of Hematology, Guangzhou Twelfth People's Hospital, Guangzhou, China; ^3^School of Basic Medicine and Public Health, Jinan University, Guangzhou, China; ^4^School of Public Health, Sun Yat-sen University, Guangzhou, China; ^5^Department of Occupational Health and Medicine, School of Public Health, Southern Medical University, Guangzhou, China; ^6^Department of Epidemiology, School of Public Health, Southern Medical University, Guangzhou, China

**Keywords:** firearm violence, Global Burden of Disease, estimated annual percentage change, age-standardized rate, sociodemographic index

## Abstract

**Background:**

Gaps remained in the updated information of the firearm violence (FV) burden from a global landscape. Understanding the global burden of FV could contribute to decision-making.

**Methods:**

Data on the FV burden, including physical violence by firearm (PVF), self-harm by firearm (SHF), and unintentional firearm injuries (UFI), were extracted from the Global Burden of Disease 2019. The temporal trends of age-standardized rate (ASR) were estimated using estimated annual percentage change (EAPC).

**Results:**

In 2019, PVF, SHF, and UFI reported 710.64 × 10^3^, 335.25 × 10^3^, and 2,133.88 × 10^3^, respectively, incident cases worldwide. Their ASR (/100,000 people-years) were 9.31, 4.05, and 28.07. During 1990–2019, the overall incident ASRs of PVF presented an increasing trend (EAPC = 0.61, 95% confidence interval [CI]: 0.48 to 0.75). Notably, pronounced increasing trends were observed in Tropical Latin America, and North Africa and Middle East. However, incident trends of SHF and UFI declined globally, with the respective EAPCs being −0.68 (95% CI: −0.83 to −0.54) and −0.98 (95% CI: −1.19 to −0.77). In 2019, the ASR of death due to PVF, SHF, and UFI were 2.23, 0.65, and 0.26, and that of DALYs were 127.56, 28.10, and 17.64, respectively. Decreasing trends in the ASRs of FV were observed in most regions and countries worldwide over the past three decades, particularly that of PVF in Estonia.

**Conclusion:**

The FV burden was heterogeneous across regions and countries, which was deeply subjected to socioeconomic factors. The findings highlighted that specific prevention strategies and interventions were required, particularly in the high prevalent settings.

## Introduction

Firearm violence (FV) mainly included physical violence, self-harm, and unintentional injury by firearm, which has been recognized as a substantial threat to public health due to its high prevalence and economic loss (1, 2). The Global Burden of Disease study (GBDs) reported that physical violence, self-harm, and unintentional injury by firearm respectively caused 174.4 × 10^3^, 63.8 × 10^3^, and 22.6 × 10^3^ deaths globally in 2017 (3). The FV burden heterogeneously varied among regions and countries. It was estimated that over 50% of total firearm injury deaths in 2016 were contributed from several countries with a high prevalence, including Brazil, the United States, Colombia, Mexico, Venezuela, and Guatemala (4). Many complex socioeconomic factors are involved in the FV burden, including military conflicts and unrest in the Middle East over the past years (5–7), and economic plight and drug violence in Latin America (8, 9).

Firearm violence frequently led to long-term disability and psychological trauma (10–12), and brought enormous economic costs. Peters et al. projected that firearm-related fatalities would lead to an estimated loss of 239 billion dollars in 36 Economic Cooperation and Development (OECD) countries from 2018 to 2030, 48.5% of which would contribute from physical violence (13). FV was regarded as a public health priority that needed to be addressed urgently (14–16).

Gaps remained in comprehensive understanding about the current status of the FV burden and its changing trends from a global landscape. Importantly, tracking the changes of the FV burden was required for health strategies. Therefore, this work aimed to investigate the global variation of the FV burden, and estimate the changing trends from 1990 to 2019 using the updated GBD data.

## Materials and methods

### Data source

The firearm violent factors mainly included physical violence by firearm (PVF), self-harm by firearm (SHF), and unintentional firearm injuries (UFI). PVF encompasses a variety of bodily harms due to firearm resulting in injury or death. Data of FV were retrieved from the Global Health Data Exchange query tool (http://ghdx.healthdata.org/gbd-results-tool), using the collective terms of “physical violence by firearm,” “self-harm by firearm,” and “unintentional firearm injuries.” Data were processed using a Bayesian meta-regression model Dismod-MR II, providing robust and reliable estimation of the epidemiology of diseases and causes. According to the GBD instructions, the number and rate of incidence, death, and disability adjusted life years (DALYs) of PVF, SHF, and UFI were extracted by sex, age, and multiple geographical levels from 1990 to 2019, without any inclusion/exclusion criteria. Data of the FV burden were available globally in 21 geographical regions and 204 countries/territories.

Sociodemographic index (SDI) is a compound indicator that reflects the strong correlations between social development and health outcomes. The SDI value ranged between 0 and 1, reflecting the lowest and highest level of average per capita incomes, educational opportunities, and fertility rates. In 2019, the SDI value ranged from 0.081 in Somalia to 0.929 in Switzerland. According to the SDI standards, these regions and countries were divided into five levels, namely, low, low-middle, middle, high-middle, and high.

### Statistical analysis

When data involved different age structures and populations over time, age-standardized estimates are necessary for cross-sectional comparisons. The age-standardized rate (ASR) per 100,000 person-years was calculated as the following formula:


$$\begin{array}{l} ASR=\frac{\sum_{i=1}^{A}a_{i}w_{i}}{\sum_{i=1}^{A}w_{i}}\times 100,000 \end{array}$$

In the above mentioned formula, *a*_*i*_ is the age-specific rate of the *i*^th^ age group; *w* is the population numbers in the corresponding *i*^th^ age group among the GBD standard population; *A* is the number of age groups.

The estimated annual percentage change (EAPC) is estimated to quantify the changing trend of ASR, which are commonly used in public health research (17). EAPC is estimated in the following steps. First, the natural logarithm of ASR is calculated to be linearly regressed with time, where y is the natural logarithm of ASR, and x is the corresponding calendar year. Then, EAPC and its 95% confidence interval (CI) are estimated using a linear regression model.


$$\begin{array}{lll} y & = & \alpha +\beta x+\varepsilon \\ EAPC & = & 100\times \left[exp\left(\beta \right)-1\right] \end{array}$$

The determination of trends is judged as follows: (1) if both the EAPC value and 95% CI > 0, it is regarded as an increasing trend; (2) if both the EAPC value and 95% CI <0, it is regarded as a decreasing trend; (3) others are regarded as stable over time. To explore the influential factors of EAPC, the associations between ASRs and SDI among regions were calculated using a Pearson correlation analysis. Data were analyzed using R version 3.6.2 (Institute for Statistical Computing, Vienna, Austria). A *p*-value of < 0.05 is regarded as statistically significant.

## Results

### Analysis on the burden and trends of PVF

**Figure 1:**
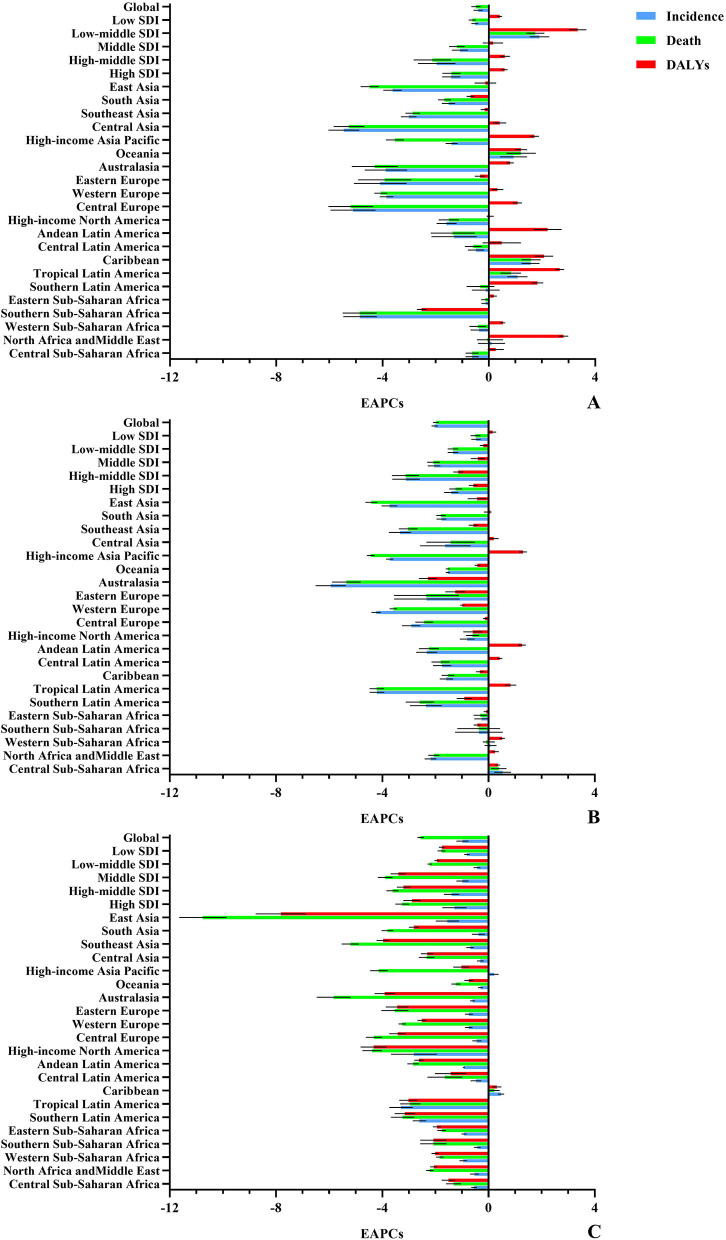
Trends in the ASR of the FV burden, 1990–2019. The FV burden included incidence, death, and DALYs of PVF **(A)**, SHF **(B)**, and UFI **(C)**, in the period 1990–2019. PVF, physical violence; SHF, self-harm by firearm; UFI, unintentional firearm injuries; DALYs, disability-adjusted life years.

**Figure 2:**
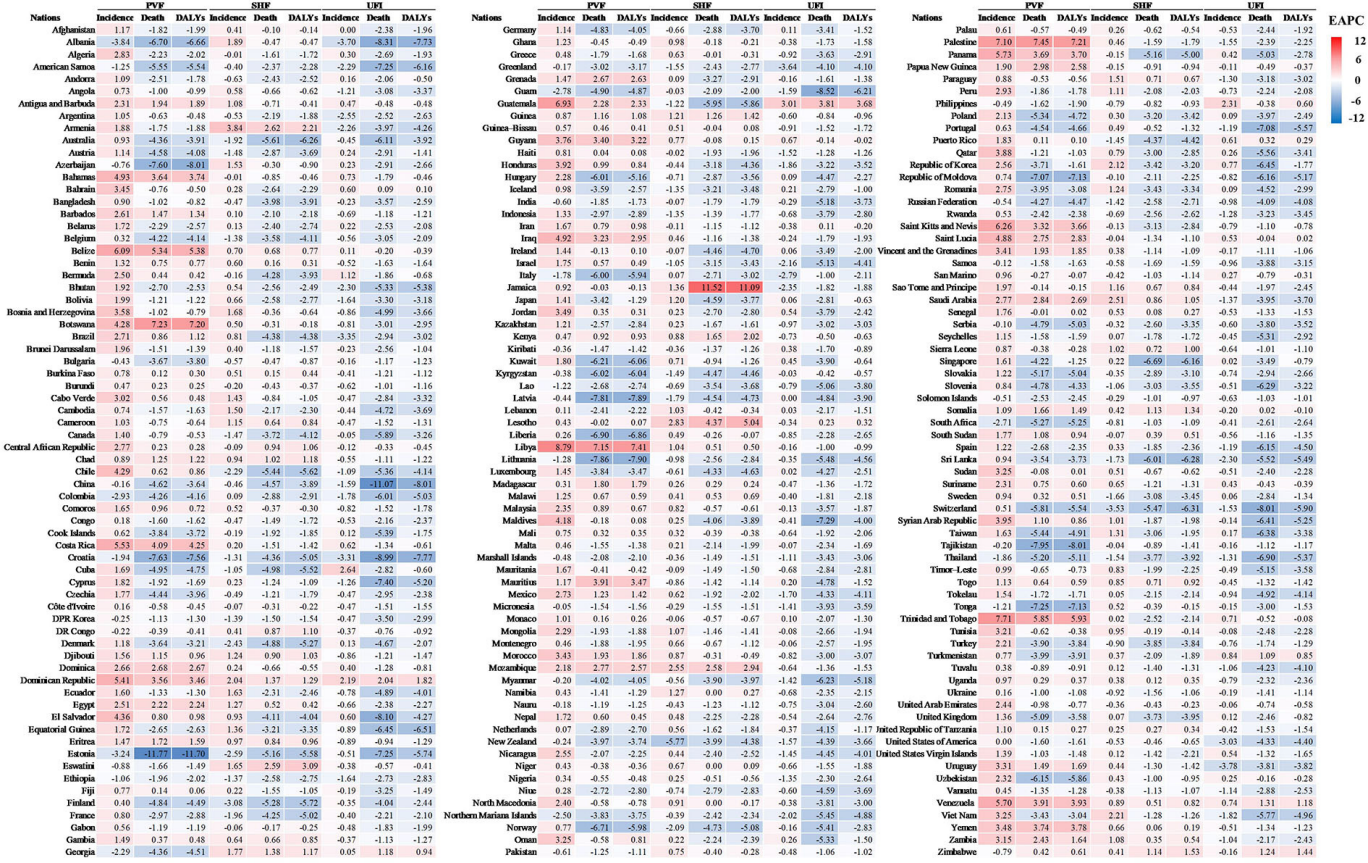
The heatmap of EAPCs of incidence, death, and DALYs of PVF, SHF, and UFI at the national level. Red indicated higher value of EAPCs, while blue indicated lower one. PVF, physical violence; SHF, self-harm by firearm; UFI, unintentional firearm injuries; DALYs, disability-adjusted life years; EAPC, estimated annual percentage change.

The overall age-standardized incidence rate (ASIR) increased from 7.22 to 9.31 over the past three decades, with an annual average increase of 0.61% (EAPC = 0.61, 95% CI: 0.48 to 0.75) (Table 1, Figure 1A). Compared with women, men had a 4-fold higher incident number, and a larger rising trend of ASIRs (Table 1). Among the age groups, the youths aged 15–29 years undertook the major proportion of PVF incidence (Supplementary Table S1). The ASIRs varied from 0.98 in central Sub-Saharan Africa to 63.35 in Tropical Latin America in 2019. Upward trends of ASIRs appeared in 13 regions, particularly North Africa and the Middle East. However, downward trends occurred in four settings, including East Asia and South Asia (Table 1, Figure 1A). The incident pattern of PVF was heterogeneous across countries. Brazil undertook the highest incident number, followed by the United States of America and China in 2019. The ASIRs ranged from 0.84 in the Democratic Republic of the Congo to 147.41 in Venezuela in 2019. During 1990–2019, rising trends in the ASIRs of PVF were observed in 156 countries/territories, and the most pronounced ones occurred in Libya (EAPC = 8.79, 95% CI: 7.44 to 10.16). In contrast, trends declined in 27 countries, being significant in Albania and Estonia (Figure 2, Supplementary Table S4 and Supplementary Figure S2).

**Table 1:** Global incident burden and trends of physical violence by firearm in sexes, SDI areas, and regions, 1990–2019.

	***1990***		***2019***		***1990–2019***	
**Characteristics**	**Number ×10^3^ (95% UI)**	**ASR/100,000** **(95% UI)**	**Number ×10^3^ (95% UI)**	**ASR/100,000** **(95% UI)**	**Percentage** **(%)**	**EAPC** **(95%CI)**
**Overall**	401.80 (300.77 to 538.82)	7.22 (5.50 to 9.46)	710.64 (535.67 to 932.43)	9.31 (7.02 to 12.28)	76.87	0.61 (0.48 to 0.75)
**Sex**						
Male	298.34 (221.56 to 397.75)	10.78 (8.16 to 14.06)	565.28 (424.34 to 729.12)	14.68 (11.12 to 18.98)	89.48	0.76 (0.58 to 0.95)
Female	103.46 (74.24 to 143.7)	3.72 (2.72 to 5.10)	145.36 (107.45 to 197.84)	3.89 (2.84 to 5.34)	40.50	0.03 (−0.06 to 0.12)
**SDI**						
Low	11.26 (8.09 to 15.53)	2.34 (1.82 to 2.96)	30.45 (21.66 to 42.25)	2.68 (2.04 to 3.48)	170.34	0.41 (0.34 to 0.48)
Low–middle	41.57 (31.88 to 55.16)	3.75 (2.96 to 4.75)	160.07 (125.41 to 203.39)	8.67 (6.87 to 10.82)	285.05	3.34 (3.04 to 3.65)
Middle	140.12 (106.47 to 189.90)	7.71 (5.99 to 10.13)	234.12 (176.84 to 305.18)	10.09 (7.71 to 13.23)	67.08	0.16 (−0.21 to 0.52)
High–middle	81.04 (59.02 to 111.71)	7.06 (5.15 to 9.79)	117.61 (87.4 to 157.71)	9.46 (6.92 to 13.11)	45.13	0.60 (0.43 to 0.78)
High	127.52 (88.90 to 179.83)	15.79 (10.95 to 22.38)	167.84 (118.20 to 234.51)	18.79 (12.91 to 26.48)	31.62	0.59 (0.48 to 0.69)
**Regions**						
East Asia	67.06 (44.76 to 97.85)	5.51 (3.68 to 8.08)	78.89 (55.01 to 110.47)	6.26 (4.28 to 9.18)	17.63	−0.13 (−0.52 to 0.26)
South Asia	11.40 (7.88 to 16.37)	1.43 (1.06 to 1.92)	19.46 (13.61 to 28.29)	1.23 (0.88 to 1.72)	70.62	−0.69 (−0.82 to −0.56)
Southeast Asia	24.38 (17.82 to 32.12)	5.63 (4.20 to 7.33)	40.60 (29.77 to 53.21)	6.00 (4.46 to 7.82)	66.55	−0.16 (−0.29 to −0.03)
Central Asia	1.84 (1.35 to 2.52)	2.49 (1.89 to 3.30)	2.53 (1.71 to 3.75)	2.79 (1.91 to 4.10)	36.95	0.41 (0.17 to 0.64)
High–incomeAsia Pacific	6.20 (4.06 to 9.69)	3.92 (2.55 to 6.13)	9.81 (6.84 to 13.61)	6.20 (4.00 to 9.67)	58.17	1.71 (1.56 to 1.87)
Oceania	0.11 (0.07 to 0.16)	1.81 (1.31 to 2.50)	0.32 (0.22 to 0.48)	2.60 (1.86 to 3.69)	193.49	1.21 (1.00 to 1.42)
Australasia	1.22 (0.83 to 1.81)	6.37 (4.29 to 9.51)	2.11 (1.49 to 3.05)	8.02 (5.30 to 12.38)	72.40	0.80 (0.68 to 0.92)
Eastern Europe	7.60 (5.29 to 10.95)	3.68 (2.52 to 5.33)	6.21 (4.42 to 8.85)	3.52 (2.34 to 5.24)	−18.28	−0.33 (−0.51 to −0.15)
Western Europe	25.57 (17.63 to 36.32)	7.22 (4.86 to 10.66)	30.33 (21.14 to 42.41)	8.14 (5.42 to 12.15)	18.64	0.33 (0.13 to 0.53)
Central Europe	4.40 (3.11 to 6.27)	3.96 (2.72 to 5.68)	4.93 (3.42 to 7.06)	5.78 (3.76 to 8.95)	11.90	1.08 (0.92 to 1.23)
High–income North America	98.81 (67.11 to 141.43)	35.11 (23.56 to 50.68)	122.58 (83.28 to 171.87)	36.53 (24.42 to 52.37)	24.05	0.05 (−0.06 to 0.17)
Andean Latin America	1.88 (1.46 to 2.41)	4.82 (3.86 to 6.01)	5.63 (4.25 to 7.37)	8.69 (6.54 to 11.38)	199.07	2.21 (1.71 to 2.72)
Central Latin America	63.76 (48.83 to 106.29)	36.60 (28.88 to 58.29)	145.81 (115.80 to 180.67)	56.02 (44.71 to 69.08)	128.69	0.48 (−0.22 to 1.19)
Caribbean	6.17 (4.94 to 7.68)	15.80 (12.81 to 19.39)	11.98 (9.71 to 14.87)	25.34 (20.57 to 31.48)	93.97	2.08 (1.75 to 2.40)
Tropical Latin America	46.77 (32.64 to 65.41)	29.20 (20.86 to 39.90)	143.05 (98.01 to 196.49)	63.35 (43.32 to 87.40)	205.83	2.67 (2.52 to 2.82)
Southern Latin America	4.50 (3.43 to 5.81)	8.93 (6.85 to 11.46)	11.21 (8.57 to 14.33)	17.50 (13.41 to 22.53)	149.01	1.83 (1.62 to 2.03)
Eastern Sub–Saharan Africa	4.55 (3.11 to 6.49)	2.61 (2.00 to 3.39)	10.40 (7.09 to 14.89)	2.81 (2.14 to 3.65)	128.49	0.18 (0.08 to 0.28)
Southern Sub–Saharan Africa	5.62 (4.04 to 7.68)	11.66 (8.52 to 15.68)	4.67 (3.35 to 6.40)	6.10 (4.44 to 8.26)	−16.96	−2.53 (−2.69 to −2.36)
Western Sub–Saharan Africa	5.68 (3.60 to 8.53)	2.51 (1.80 to 3.41)	15.37 (9.53 to 23.16)	2.95 (2.07 to 4.17)	170.71	0.53 (0.45 to 0.60)
North Africaand Middle East	13.73 (9.25 to 19.93)	3.39 (2.43 to 4.65)	43.35 (31.24 to 59.12)	7.20 (5.26 to 9.73)	215.68	2.81 (2.64 to 2.97)
Central Sub–Saharan Africa	0.52 (0.33 to 0.80)	0.85 (0.61 to 1.20)	1.41 (0.88 to 2.11)	0.98 (0.69 to 1.38)	173.19	0.26 (−0.03 to 0.55)

EAPC, estimated annual percentage change; ASR, age–standardized rate; CI, confidence interval; UI, uncertainty interval; SDI, sociodemographic index.

The overall ASRs of death and DALYs were 2.23 and 127.56 in 2019, and presented downward trends from 1990 to 2019, with the respective EAPCs of −0.49 (95% CI: −0.64 to −0.33) and −0.40 (95%CI: −0.56 to −0.24) (Supplementary Table S5, Figure 1A). Men had far more number of deaths and DALYs than women, but women had more pronounced downward trends of the ASRs (Supplementary Table S5). Among the age groups, those aged 15–44 years undertook the greatest health loss due to PVF (Supplementary Table S1 and Supplementary Figures S1B-C). In 2019, the ASRs of death and DALYs due to PVF ascended from high-income Asia Pacific to Tropical Latin America. Similar decreasing trends in the ASRs of death and DALYs occurred in most regions from 1990 to 2019, particularly Central Asia, whereas only three regions showed increasing trends, including the Caribbean, Oceania, and Tropical Latin America (Supplementary Table S5, Figure 1A). Among 204 countries/territories, the highest ASRs of death and DALYs due to PVF were seen in Latin American countries, including El Salvador and Venezuela. In the past 30 years, downward trends in ASRs of death and DALYs occurred in more than 120 countries/territories, and the largest ones occurred in Estonia. In contrast, upward trends were seen in over 50 countries, particularly Palestine, Botswana, and Libya (Figure 2, Supplementary Table S6, and Supplementary Figure S2).

### Analysis on the burden and trends of SHF

Globally, the ASIR of SHF was 4.05 in 2019, and presented a decreasing trend from 1990 to 2019 (EAPC = −0.68, 95% CI: −0.83 to −0.54). Compared with women, men had a higher decreasing trend of ASIR (Table 2, Figure 1B). Among the age groups, the adults aged 40–45 years had the highest number of ASIRs, and people over 80 years had the most pronounced increase (Supplementary Table S2, Supplementary Figure S1D). Regionally, the ASIRs varied from 1.01 in Southeast Asia to 10.64 in Eastern Europe in 2019. During the period of 1990–2019, trends in ASIRs of SHF declined in twelfth regions, but rose in the other eight regions (Table 2, Figure 1B). Nationally, the ASIRs of SHF varied from 0.27 in Indonesia to 12.12 in Ukraine in 2019. In the past three decades, trends in the ASIRs of SHF rose in 111 countries/territories, particularly Armenia. However, trends declined in 68 countries, and the most pronounced ones were seen in New Zealand (EAPC = −5.77, 95%CI: −6.37 to −5.17), followed by Switzerland and Finland (Figure 2, Supplementary Table S7, Supplementary Figures S3A–B).

**Table 2:** Global incident burden and trends in self–harm by firearm and unintentional firearm injuries in sexes, SDI areas, and regions 1990–2019.

**Characteristics**	**Self–harm by firearm**				**Unintentional firearm injuries**			
	***2019***		***1990–2019***		***2019***		***1990–2019***	
	**Number ×10^3^** **(95% UI)**	**ASR/100,000** **(95% UI)**	**Percentage** **(%)**	**EAPC** **(95%CI)**	**Number ×10^3^** **(95% UI)**	**ASR/100,000** **(95% UI)**	**Percentage** **(%)**	**EAPC** **(95%CI)**
**Overall**	335.25 (225.62 to 470.37)	4.05 (2.73 to 5.67)	53.76	−0.68 (−0.83 to −0.54)	2133.88 (1490.43 to 2933.36)	28.07 (19.60 to 39.20)	10.75	−0.98 (−1.19 to −0.77)
**Sex**								
Male	177.52 (125.55 to 242.11)	4.34 (3.08 to 5.90)	43.59	−1.02 (−1.13 to −0.92)	1278.94 (923.79 to 1728.40)	33.33 (24.03 to 45.16)	6.85	−1.12 (−1.30 to −0.94)
Female	157.73 (97.77 to 238.77)	3.77 (2.32 to 5.68)	67.08	−0.23 (−0.49 to 0.03)	854.94 (555.15 to 1236.91)	22.89 (14.65 to 33.55)	17.16	−0.77 (−1.01 to −0.52)
**SDI**								
Low	20.50 (13.99 to 28.82)	2.68 (1.88 to 3.71)	133.92	0.15 (0.04 to 0.27)	300.68 (203.51 to 442.40)	26.85 (19.49 to 36.74)	81.40	−0.81 (−0.90 to −0.73)
Low–middle	73.42 (47.54 to 106.35)	4.37 (2.89 to 6.28)	89.92	−0.19 (−0.31 to −0.07)	428.54 (304.33 to 596.37)	24.46 (17.62 to 33.60)	34.87	−0.43 (−0.55 to −0.32)
Middle	92.36 (55.44 to 136.92)	3.43 (2.1 to 5.05)	74.89	−0.40 (−0.65 to −0.15)	748.76 (515.3 to 1039.60)	32.52 (22.12 to 46.20)	3.99	−0.98 (−1.19 to −0.77)
High–middle	99.27 (65.73 to 144.32)	5.29 (3.50 to 7.62)	24.44	−1.14 (−1.31 to −0.97)	384.91 (271.02 to 524.85)	28.77 (19.87 to 40.30)	−9.40	−1.39 (−1.66 to −1.12)
High	49.59 (36.52 to 67.54)	3.69 (2.68 to 4.99)	30.65	−0.57 (−0.72 to −0.43)	267.55 (184.39 to 379.19)	27.57 (18.44 to 39.75)	−9.62	−1.28 (−1.72 to −0.84)
**Regions**								
East Asia	105.4 (50.16 to 174.53)	5.29 (2.47 to 8.68)	55.39	−0.43 (−0.77 to −0.09)	377.76 (255.17 to 526.33)	28.24 (18.86 to 39.71)	−12.13	−1.55 (−1.97 to −1.13)
South Asia	92.99 (60.11 to 136.31)	5.26 (3.43 to 7.68)	107.74	−0.03 (−0.16 to 0.09)	189.75 (120.39 to 280.71)	10.63 (6.87 to 15.54)	45.48	−0.38 (−0.61 to −0.15)
Southeast Asia	7.26 (4.86 to 10.34)	1.01 (0.67 to 1.42)	84.25	−0.57 (−0.73 to −0.41)	298.41 (194.49 to 429.74)	45.24 (29.28 to 66.38)	13.23	−0.69 (−0.82 to −0.57)
Central Asia	2.27 (1.61 to 3.10)	2.55 (1.83 to 3.43)	75.15	0.20 (0.04 to 0.36)	15.20 (10.40 to 21.53)	16.48 (11.34 to 23.13)	20.04	−0.31 (−0.42 to −0.20)
High–income Asia Pacific	7.58 (5.05 to 11.23)	2.73 (1.79 to 3.98)	82.49	1.30 (1.17 to 1.43)	43.95 (29.69 to 62.65)	24.30 (15.75 to 35.76)	8.80	0.21 (0.06 to 0.36)
Oceania	0.35 (0.24 to 0.48)	2.96 (2.14 to 3.99)	114.33	−0.42 (−0.51 to −0.33)	5.26 (3.44 to 7.69)	35.46 (24.14 to 49.82)	85.88	−0.30 (−0.38 to −0.23)
Australasia	0.53 (0.38 to 0.71)	1.42 (1.00 to 1.94)	3.22	−2.28 (−2.6 to −1.96)	4.10 (2.72 to 5.84)	15.06 (9.69 to 22.30)	17.48	−0.60 (−0.68 to −0.53)
Eastern Europe	30.96 (21.73 to 43.64)	10.64 (7.37 to 14.96)	−16.41	−1.25 (−1.61 to −0.89)	36.72 (25.24 to 51.45)	18.41 (12.26 to 26.86)	−24.62	−0.74 (−0.87 to −0.60)
Western Europe	21.69 (16.67 to 27.95)	3.50 (2.64 to 4.59)	4.94	−0.98 (−1.05 to −0.92)	79.61 (53.43 to 115.32)	18.42 (11.85 to 27.11)	−2.07	−0.74 (−0.87 to −0.62)
Central Europe	9.36 (6.99 to 12.25)	5.40 (3.99 to 7.08)	19.41	−0.13 (−0.19 to −0.07)	21.07 (14.53 to 29.87)	18.29 (12.32 to 26.13)	−14.03	−0.44 (−0.59 to −0.28)
High–income North America	17.31 (11.92 to 24.38)	3.95 (2.70 to 5.62)	38.70	−0.59 (−0.93 to −0.25)	117.38 (78.91 to 166.92)	33.57 (22.17 to 49.42)	−32.32	−2.81 (−3.65 to −1.96)
Andean Latin America	0.96 (0.70 to 1.27)	1.54 (1.13 to 2.03)	193.81	1.26 (1.13 to 1.39)	45.83 (33.30 to 62.81)	71.42 (52.17 to 96.94)	31.11	−0.93 (−0.95 to −0.91)
Central Latin America	4.35 (3.19 to 5.76)	1.69 (1.24 to 2.24)	123.72	0.42 (0.34 to 0.50)	179.25 (131.15 to 242.36)	71.11 (52.33 to 95.95)	18.72	−0.47 (−0.66 to −0.29)
Caribbean	1.38 (1.04 to 1.8)	2.73 (2.04 to 3.54)	55.29	−0.33 (−0.46 to −0.20)	70.73 (51.99 to 94.68)	148.71 (109.21 to 199.22)	51.76	0.47 (0.37 to 0.57)
Tropical Latin America	5.06 (3.49 to 7.20)	2.02 (1.40 to 2.89)	147.48	0.83 (0.63 to 1.03)	66.05 (47.01 to 91.59)	29.60 (20.98 to 41.27)	−34.78	−3.29 (−3.72 to −2.86)
Southern Latin America	3.10 (2.45 to 3.86)	4.15 (3.27 to 5.18)	42.96	−0.91 (−1.18 to −0.64)	49.46 (35.37 to 69.04)	73.39 (51.78 to 102.46)	−20.89	−2.60 (−2.84 to −2.36)
Eastern Sub– Saharan Africa	4.08 (2.91 to 5.61)	1.90 (1.36 to 2.60)	122.84	−0.09 (−0.17 to −0.01)	135.57 (90.46 to 201.23)	35.43 (26.16 to 47.75)	82.58	−0.92 (−1.00 to −0.84)
Southern Sub– Saharan Africa	2.17 (1.46 to 3.14)	3.03 (2.07 to 4.32)	72.7	−0.42 (−0.54 to −0.31)	15.66 (10.23 to 23.48)	19.95 (13.24 to 29.53)	27.63	−0.42 (−0.53 to −0.31)
Western Sub– Saharan Africa	4.19 (2.94 to 5.83)	1.70 (1.21 to 2.37)	163.99	0.51 (0.43 to 0.60)	130.65 (83.92 to 200.07)	28.61 (20.24 to 39.90)	90.05	−0.95 (−1.08 to −0.82)
North Africa and Middle East	12.48 (8.99 to 16.94)	2.14 (1.57 to 2.85)	168.57	0.25 (0.13 to 0.37)	216.82 (153.58 to 294.33)	35.24 (25.16 to 47.43)	47.87	−0.53 (−0.68 to −0.38)
Central Sub– Saharan Africa	1.77 (1.28 to 2.40)	2.50 (1.80 to 3.40)	171.29	0.35 (0.27 to 0.42)	34.65 (21.82 to 52.39)	26.99 (19.28 to 37.34)	100.36	−0.54 (−0.63 to −0.45)

EAPC, estimated annual percentage change; ASR, age–standardized rate; CI, confidence interval; UI, uncertainty interval; SDI, socio–demographic index.

The ASRs of death and DALYs due to SHF were 0.65 and 28.10. During 1990–2019, the ASRs of death and DALYs due to SHF presented decreasing trends with the respective EAPCs of −1.97 (95% CI, −2.07 to −1.88) and −2.03 (95% CI, −2.13 to −1.92). Although undertaking far higher death and DALYs of SHF than women, men gained larger decreasing trends of the ASRs (Supplementary Table S8, Figure 1B). Among the age groups, the youths and adults responded to the largest number of deaths and DALYs (Supplementary Table S2, Supplementary Figures S1E,F). Regionally, high-income North America and South Asia undertook the harvest death and DALYs. Decreasing trends in the ASRs were observed in most regions, and the most pronounced ones occurred in Australasia (Supplementary Table S8, Figure 1B). Nationally, the United States of America, India, France, and Brazil undertook the largest number of deaths and DALYs of SHF. During 1990–2019, trends in ASRs of death and DALYs declined in most countries/territories, markedly in Singapore and Switzerland. However, increasing trends of death and DALYs were seen in over 30 countries, particularly Jamaica (Figure 2, Supplementary Table S9 and Supplementary Figures S3C–F).

### Analysis on the burden and trends of UFI

Globally, the ASIRs of UFI were 4.05 in 2019, with a decreasing trend from 1990 to 2019 (EAPC = −0.98, 95%CI: −1.19 to −0.77) (Table 2, Figure 1C). Men had a higher incident number than women, but showed a larger decreasing trend of ASIR (Table 2). The children and youths undertook the heavy number, especially those aged 1–4 years (Supplementary Table S3, Supplementary Figure S1G). At the regional levels, the ASIR ranged from 10.63 in South Asia to 148.71 in Caribbean in 2019. Decreasing trends in ASIRs of UFI were seen in most regions over the past 30 years, particularly Tropical Latin America and high-income North America (Table 2, Figure 1C). At the national levels, the ASIRs of UFI were heterogeneous from 10.07 in Pakistan to 229.75 in Antigua and Barbuda in 2019. In the past three decades, trends in the ASIR of UFI declined in 138 countries/territories, especially Uruguay and Albania. In contrast, trends rose in 40 countries, and the largest ones occurred in Guatemala, Cuba, and the Philippines (Figure 2, Supplementary Table S10 and Supplementary Figures S4A,B).

In 2019, the ASRs of death and DALYs due to UFI were 0.26 and 17.64, respectively. Trends in the overall ASRs of death and DALYs pronouncedly declined from 1990 to 2019, and their corresponding EAPCs were −2.55 (95% CI: −2.66 to −2.45) and −2.31 (95%CI: −2.44 to −2.17) (Supplementary Table S11, Figure 1C). Although the number of deaths and DALYs due to UFI were higher in women, men had larger downward trends of the ASRs (Supplementary Table S11). Those aged 15–39 years responded to a larger number of deaths and DALYs due to UFI than the other age groups (Supplementary Table S3, Supplementary Figures S1H,I). At the regional level, North Africa and Middle East and Eastern Sub-Saharan Africa suffered from the highest number of deaths and DALYs. In the period 1990–2019, downward trends in the ASRs of death and DALYs were seen in all regions, except the Caribbean (Supplementary Table S11, Figure 1C). At the national level, the highest ASRs of death and DALYs occurred in Haiti and Guatemala in 2019. In the past 30 years, the ASRs of death and DALYs presented decreasing trends in most countries, particularly China and Croatia. However, trends of death and DALYs rose only in several countries, including Guatemala, Dominican Republic, and Venezuela (Figure 2, Supplementary Table S12 and Supplementary Figures S4C–F).

### Trends of the FV burden related to sociodemographic factors

**Figure 3:**
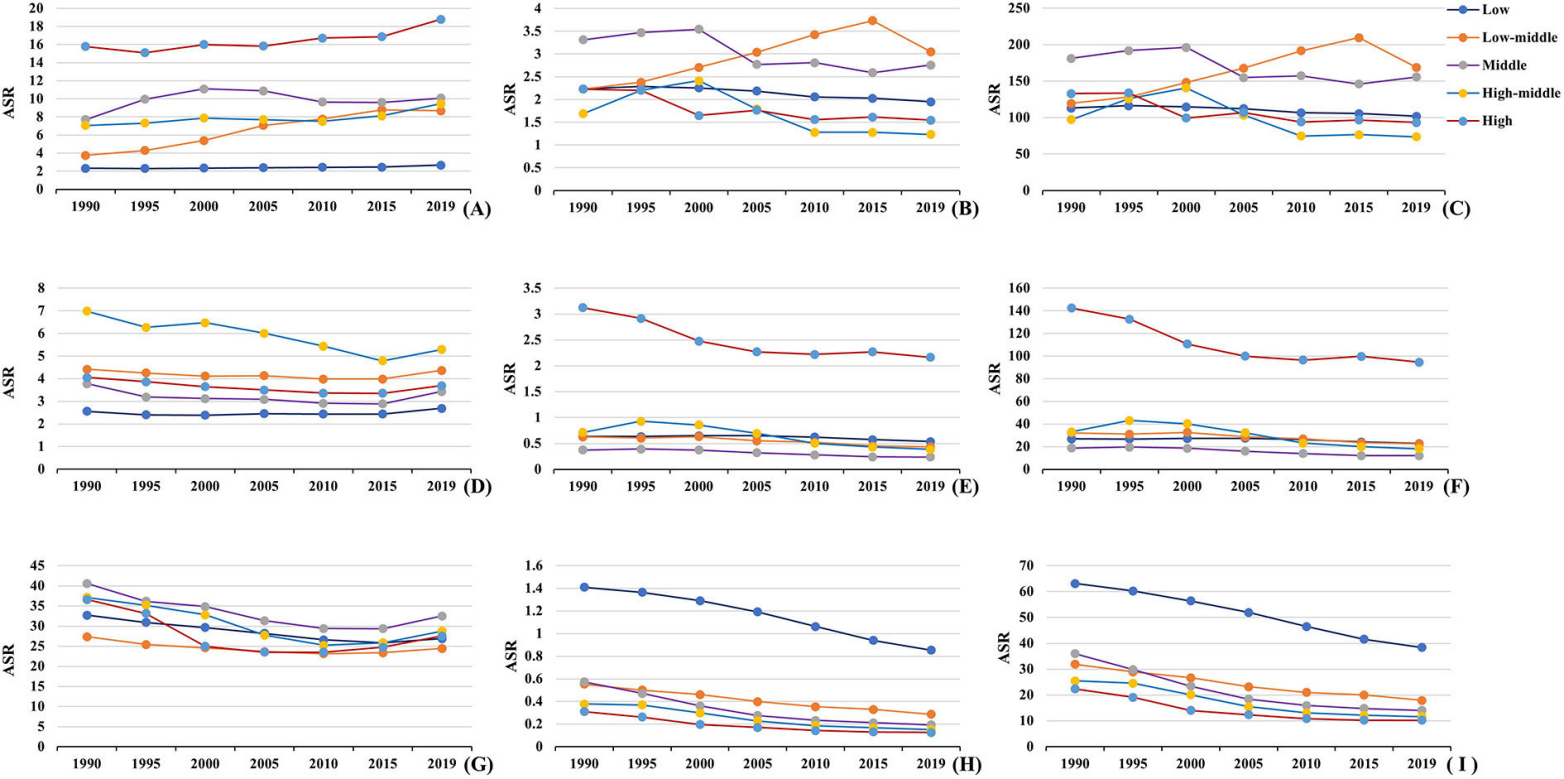
The changes in the ASRs of FV burden in SDI areas from 1990 to 2019. **(A–C)** were the ASRs of incidence, death, and DALYs of PVF. **(D–F)** were the ASRs of death, and DALYs of SHF. **(G–I)** were the ASRs of incidence, death, and DALYs of UFI, respectively. PVF, physical violence; SHF, self-harm by firearm; UFI, unintentional firearm injuries; ASR, age-standardized rate; SDI, sociodemographic index; DALYs, disability-adjusted life years.

**Figure 4:**
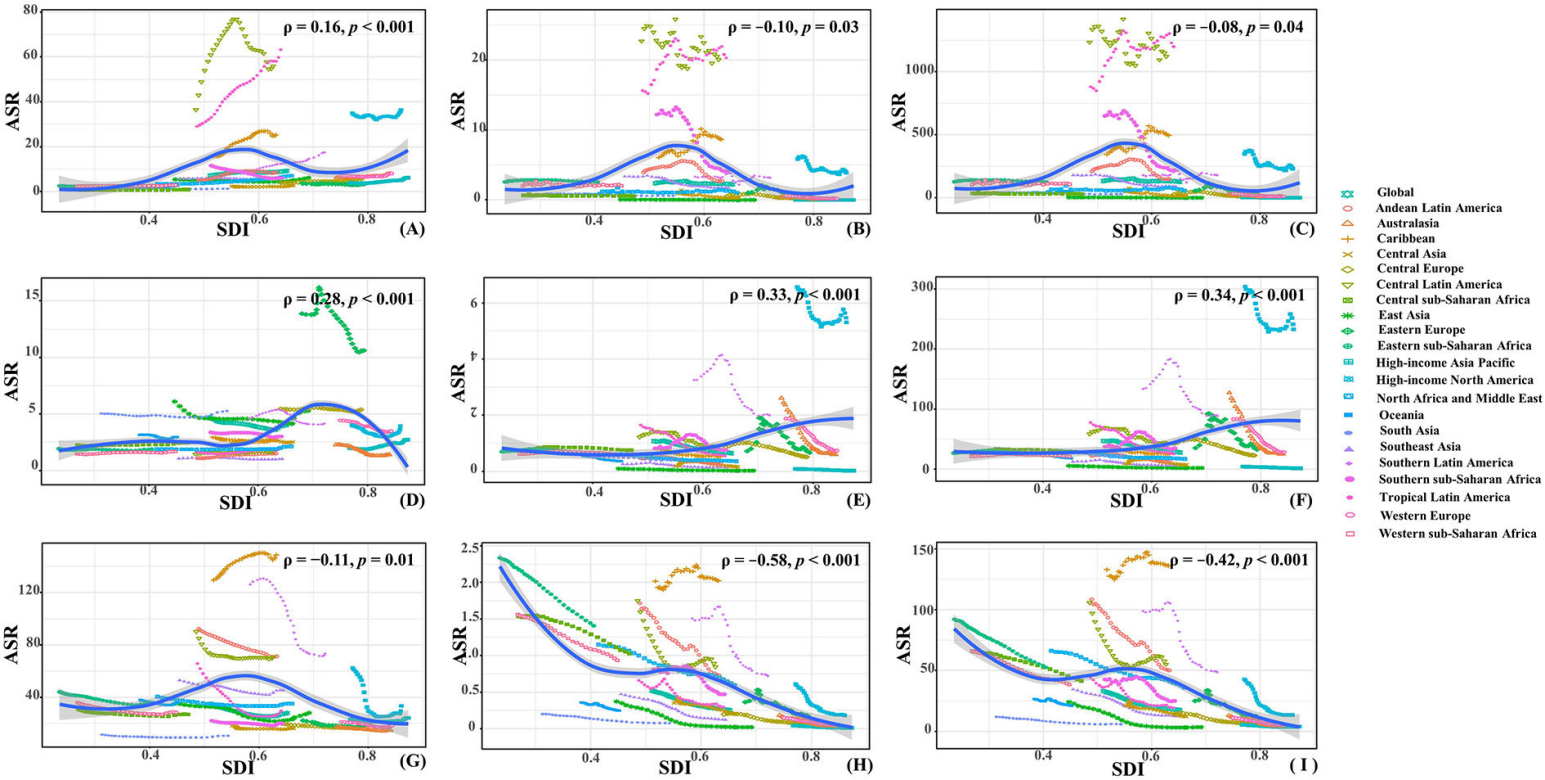
The associations between ASRs and SDI among regions. **(A–C)** were that of incidence, death, and DALYs of PVF; **(D–F)** were that of death, and DALYs of SHF; **(G–I)** were that of incidence, death, and DALYs of UFI, respectively. The association was calculated with Pearson correlation analysis. PVF, physical violence; SHF, self-harm by firearm; UFI, unintentional firearm injuries; ASR, age-standardized rate; SDI, sociodemographic index; DALYs: disability-adjusted life years.

In 2019, the high SDI area had the highest ASIR of PVF, and the low-middle and middle ones undertook the high ASRs of death and DALYs. During 1990–2019, trends of ASIR increased in most SDI areas, especially the low-middle ones (EAPC = 3.34, 95%CI: 3.04 to 3.65) (Table 1, Supplementary Table S5 and Figures 3A–C). In contrast, trends of death and DALYs declined in most SDI areas except the low-middle one. The ASIRs were positively related to SDI, but the ASRs of death were negatively related to SDI among regions (Figures 4A–C). The largest ASIR of SHF was observed in the high-middle SDI area, and trends in ASIRs of SHF declined in most SDI areas, particularly the high-middle ones. The largest ASRs of death and DALYs occurred in the year 2019. Decreasing trends of death and DALYs were observed in all SDI areas, and the largest ones occurred in the high-middle one (Table 2, Supplementary Table S8 and Figures 3D–F). The ASRs of SHF burden were positively related to SDI among regions (Figures 4D–F). The middle SDI area suffered from the highest UFI incidence, and the low one undertook the largest death and DALYs in 2019. Decreasing trends in ASRs of incidence, death, and DALYs were observed in all SDI areas, particularly that of death in middle and high-middle ones (Table 2, Supplementary Table S11 and Figures 3G-I). The ASRs of UFI burden were negatively related to SDI among regions (Figures 4G-I).

## Discussion

Comparative studies on the burden and trends of FV could provide important information to public health strategies. However, previous studies only focused on death due to FV (3, 4, 8). In the present work, the burden and trends of FV were comprehensively analyzed using the indexes of incidence, death, and DALYs with the updated data of GBD 2019.

The results highlighted that the incident trends in ASRs of PVF increased globally from 1990 to 2019, which involved many complex socioeconomic factors, including poverty, educational attainment (18, 19), drug violence, and alcohol abuse (8, 20, 21), and regional political unrest (5, 22, 23). Meanwhile, the depressed economy and unstable politics brought rapid growth of poverty population and drug violence (24). Military and political conflicts continued to erupt in North Africa and Middle East, and many civilians were killed or injured by gunfire (25). Downward trends in the ASIRs of SHF and UFI were observed in most regions and nations worldwide, and were similar to the previous study (26). In recent decades, stronger gun laws promoted the reduction of SHF and UFI (27). Meanwhile, the safe household storage of firearms remarkably declined firearm-related suicide and unintentional injuries (28). Importantly, enforced behavioral and mental health care was an effective measure to reduce the firearm suicide rate (29). Female victims of SHF and UFI were more vulnerable to psychological and sexual stress due to intimate partner violence (30), which probably explained why women achieved a lower downward trend. Nationally, the Latin American countries, including Brazil, Venezuela, Colombia, and Mexico, undertook a high incident number of PVF, probably related to the high firearm availability (31), prevalent drug and alcohol abuse (32), and socioeconomic inequality among young people (33). The most pronounced increasing trend of PVF was observed in Libya, where armed conflicts existed over the years (7), and the civilians suffered from continued violence of small arms and light weapons (34). In contrast, effective law enforcement, gun control, and improved trauma system had contributed to a significant decrease in incidence and mortality of firearm injuries since Estonian independence (35). New Zealand, Switzerland, and Finland presented the most pronounced decreasing trends, mainly due to the establishment of strict gun policies, and the SHF and UFI could be effectively prevented (36).

In the past three decades, decreasing trends in death and DALYs caused by FV were observed globally, which was similar to the previous studies (3, 4). The reasons were probably due to increasing concern for FV, and measures had been taken to strengthen the healthcare systems of firearm injury (37, 38). Firearm injuries are the leading cause of mortality among youth, and non-fatal firearm injuries are far more than fatalities, indicating that huge medical resources had to be invested in trauma emergency and long-term recovery (39). For example, the US government had embraced interventions in primary care, mental health care, and emergency departments due to the decline in non-fatal firearms-related injuries (12). In other words, high SDI areas had adequate medical care to decrease the number of the death and DALYs, which probably explained the trends of death and DALYs due to PVF negatively associated with the SDI level. Trends of death and DALYs due to UFI and SHF were probably because of the stronger firearm management among youth (40). Furthermore, the risk factors of firearm death declined in several high-prevalence Latin American countries in recent years (8). Strong measures declined in the proliferation of firearms in some countries, for example, the Brazilian government had instituted an arms confiscation policy since 2004 (9). Central European countries had suffered from social upheaval and armed violence, and caused millions of deaths in the early 1990s, but the gradual restoration of social order in recent years (41) greatly promoted the downward trends of PVF. However, in the past years, the Eastern Mediterranean Region, including Palestine, Libya, Iraq, and Yemen, had experienced several conflicts and unrests (42), and rigorous public health problems caused by population displacement, social disorganization, and the collapsed healthcare systems (43) largely brought significant upward trends of deaths and DALYs.

Several limitations should be interpreted in this study. First, the GBD studies provide a methodological and conceptual framework to quantify the comparative magnitude of health loss due to diseases, injuries, and risk factors. The accuracy and robustness of GBD estimates relied on the quality and quantity of data, which might have been impaired by potential bias, including misclassification, miscoding, and underreported cases. Second, data sources had gaps in quality and coverage in many countries, and the GBD estimates used various models to estimate for settings with sparse data, and the details reported in previous studies (4, 8). Although many limitations existed in the estimate and credibility of GBD data, the GDB studies were considered a systematic, scientific measure in health assessment. Third, due to the limitation of ASR estimation, trends of PVF burden in age groups were estimated by only using the percentage changes of absolute number. Finally, in terms of the SDI relations between ASRs and SDI, the various differences and nonlinear associations in some cases were a potential impact on the reliability of the results.

## Conclusion

The present study comprehensively analyzed the burden and trends of PVF, SHF, and UFI from a global landscape, in 1990–2019. The FV burden was still a substantial challenge to global well-being. Therefore, reducing the FV burden was urgent, and governments needed to formulate effective strategies of prevention and intervention according to specific socioeconomic factors.

## Data availability statement

The original contributions presented in the study are included in the article/Supplementary material, further inquiries can be directed to the corresponding author/s.

## Author contributions

ZO conceptualized and wrote the draft in consultation with ZW and CM. ST and SZ collected the data. DD, JZ, YS, YC, JC, and XZ collated the data. YR, KF, JL, and YZ analyzed and visualized the data. ZW is the guarantor of this manuscript. All authors reviewed the manuscript. All authors contributed to the article and approved the submitted version.

## Funding

The Major Science and Technology Project of Guangzhou Municipal Health Commission (Project Number: 2021A031003). Guangzhou Key Medical Discipline (2021-2023). Key Research and Development Programme of Guangzhou Science and Technology Project (Project Number: 202206010061).

## Conflict of interest

The authors declare that the research was conducted in the absence of any commercial or financial relationships that could be construed as a potential conflict of interest.

## Publisher's note

All claims expressed in this article are solely those of the authors and do not necessarily represent those of their affiliated organizations, or those of the publisher, the editors and the reviewers. Any product that may be evaluated in this article, or claim that may be made by its manufacturer, is not guaranteed or endorsed by the publisher.

## Abbreviations

FV: Firearm violence
PVF: Physical violence by firearm
SHF: self-harm by firearm
UFI: unintentional firearm injuries
DALYs: disability adjusted life years
GBD: Global Burden of Disease
ASR: Age-standardized rate
EAPC: Estimated annual percentage change
UI: Uncertainty interval
CI: Confidence interval
GHDx: Global Health Data Exchange
SDI: Socio-demographic index.
