# Supplementary material for: Global burden and trends of firearm violence in 204 countries/territories from 1990 to 2019
Source: Front Public Health. 2022 Aug 30;10:966507. doi: 10.3389/fpubh.2022.966507 (PMC9470124; doi:10.3389/fpubh.2022.966507)
Supplement: Supplementary file 1 [file Data_Sheet_1.docx]

**Figure S1**. The distribution of burden of physical violence by firearm, self-harm by firearm, and unintentional firearm injuries in age groups from 1990 to 2019. (A), (B), and (C) were the number of incidence, death and DALYs of physical violence by firearm; (D), (E), and (F) were the number of incidence, death and DALYs of self-harm by firearm; (G), (H), and (I) were the number of incidence, death and DALYs of unintentional firearm injuries, respectively. disability adjusted life years.

**
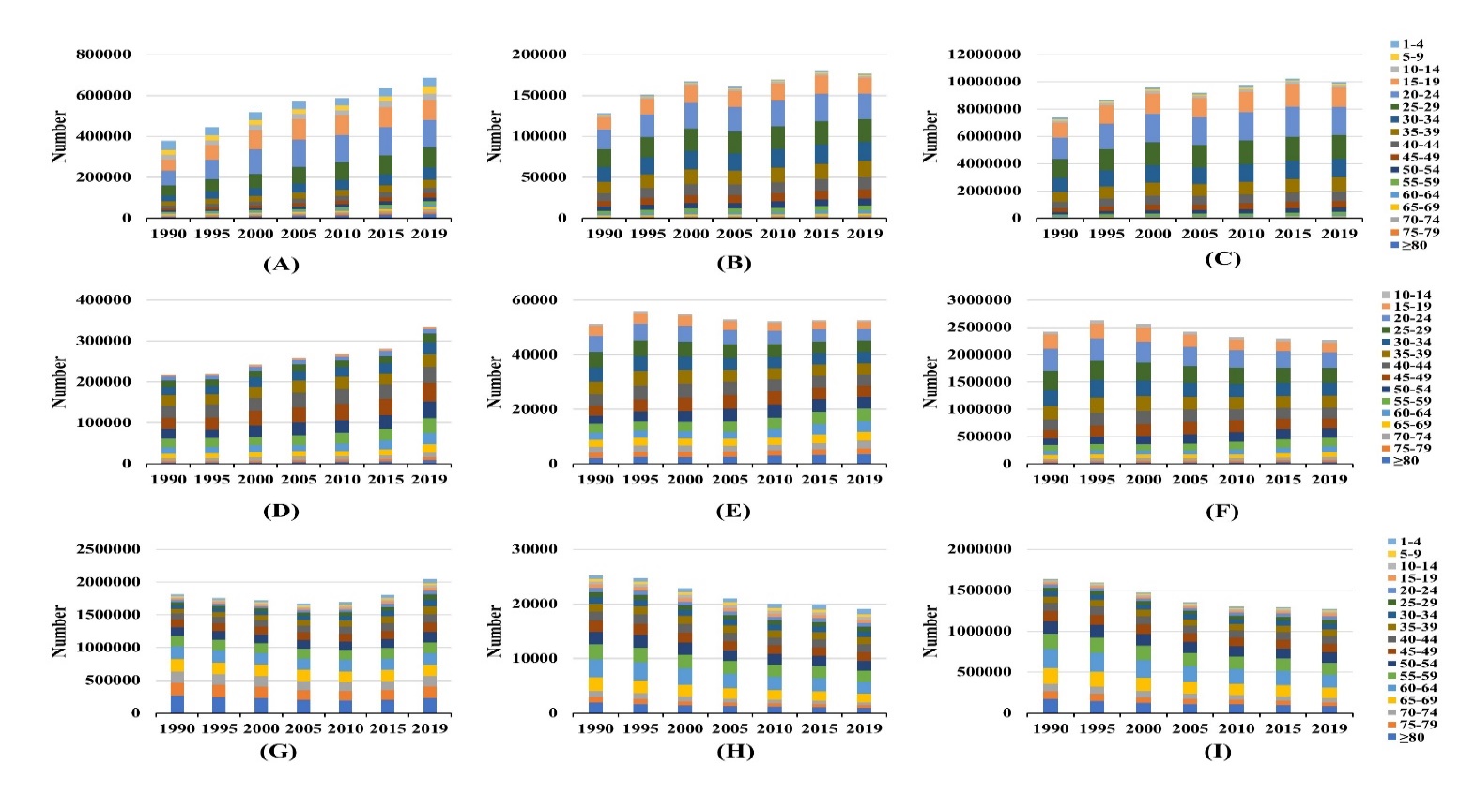
**

**Figure S2.** The distribution of ASRs and EAPCs of physical violence by firearm at the national level. (A) was the incident ASRs in 2019; (B) was the EAPCs of incidence; (C) was the ASRs of death in 2019; (D) was the EAPCs of death; (E) was the ASRs of DALYs in 2019; (F) was the EAPCs of DALYs, respectively. Countries/territories with an extreme value were annotated. ASR, age-standardized rate; EAPC, estimated annual percentage change; DALYs: disability adjusted life years.

**
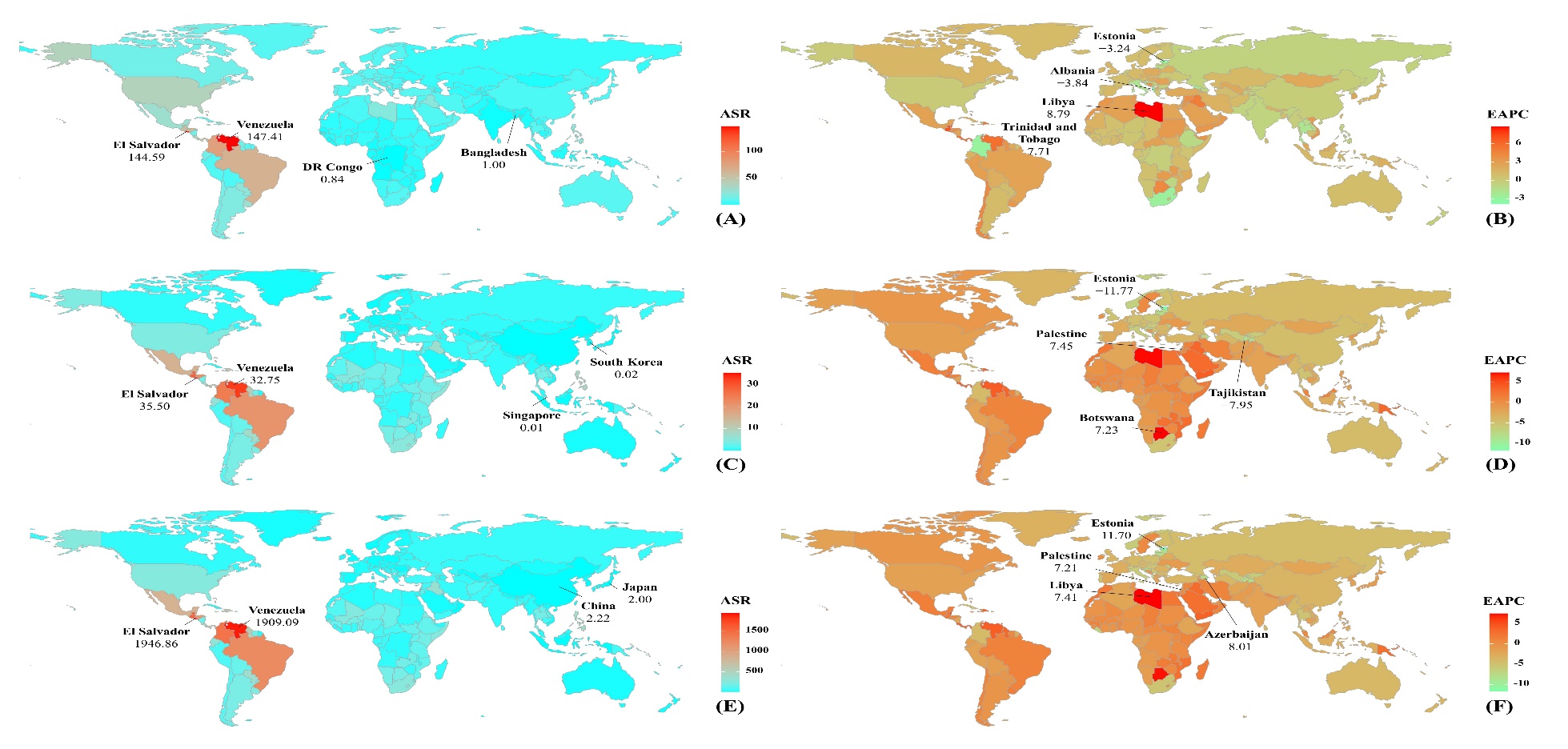
**

**Figure S3.** The distribution of ASRs and EAPCs of self-harm by firearm at the national level. (A) was the incident ASRs in 2019; (B) was the EAPCs of incidence; (C) was the ASRs of death in 2019; (D) was the EAPCs of death; (E) was the ASRs of DALYs in 2019; (F) was the EAPCs of DALYs, respectively. Countries/territories with an extreme value were annotated. ASR, age-standardized rate; EAPC, estimated annual percentage change; DALYs: disability adjusted life years.


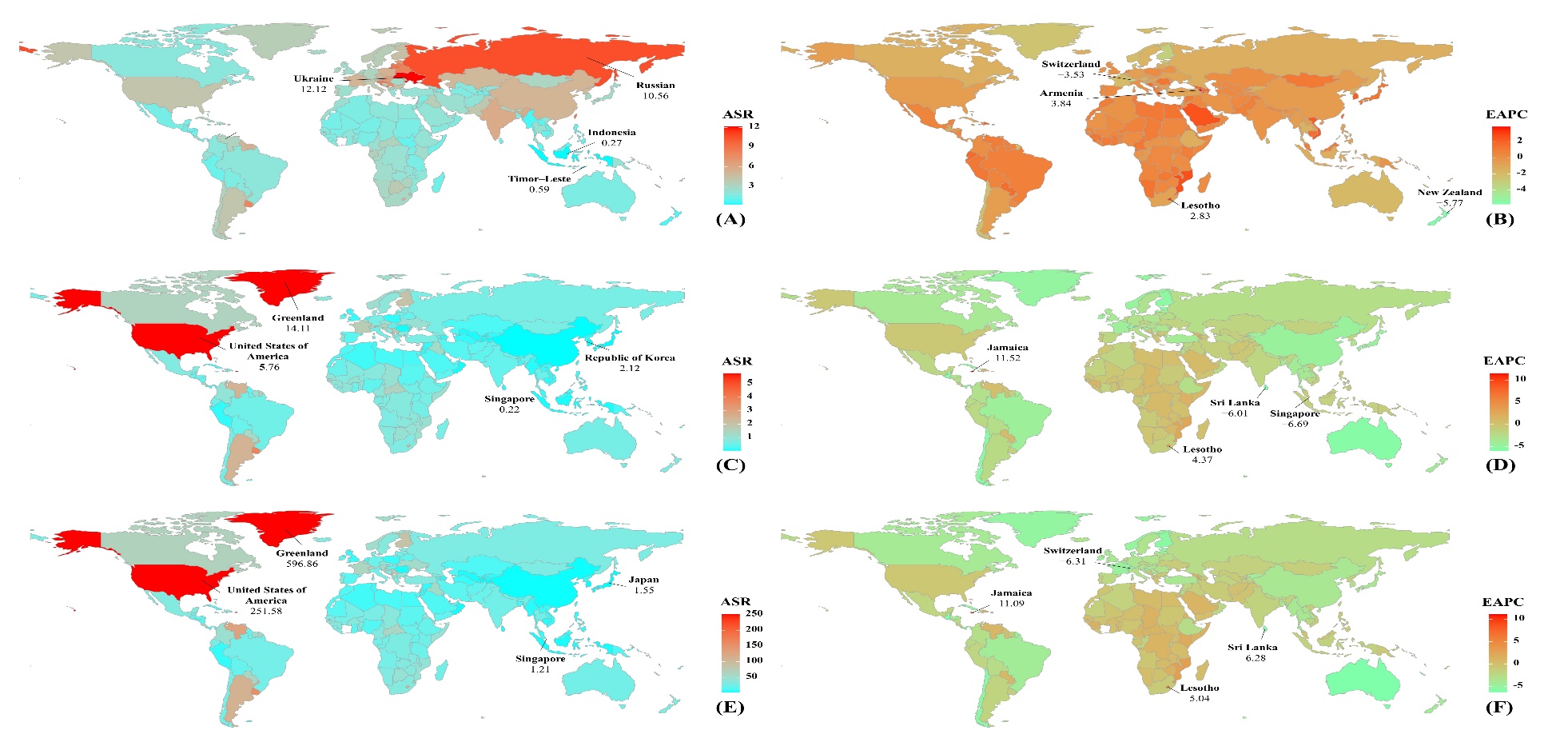


**Figure S4.** The distribution of ASRs and EAPCs of unintentional firearm injuries at the national level. (A) was the incident ASRs in 2019; (B) was the EAPCs of incidence; (C) was the ASRs of death in 2019; (D) was the EAPCs of death; (E) was the ASRs of DALYs in 2019; (F) was the EAPCs of DALYs, respectively. Countries/territories with an extreme value were annotated. ASR, age-standardized rate; EAPC, estimated annual percentage change; DALYs: disability adjusted life years.

**
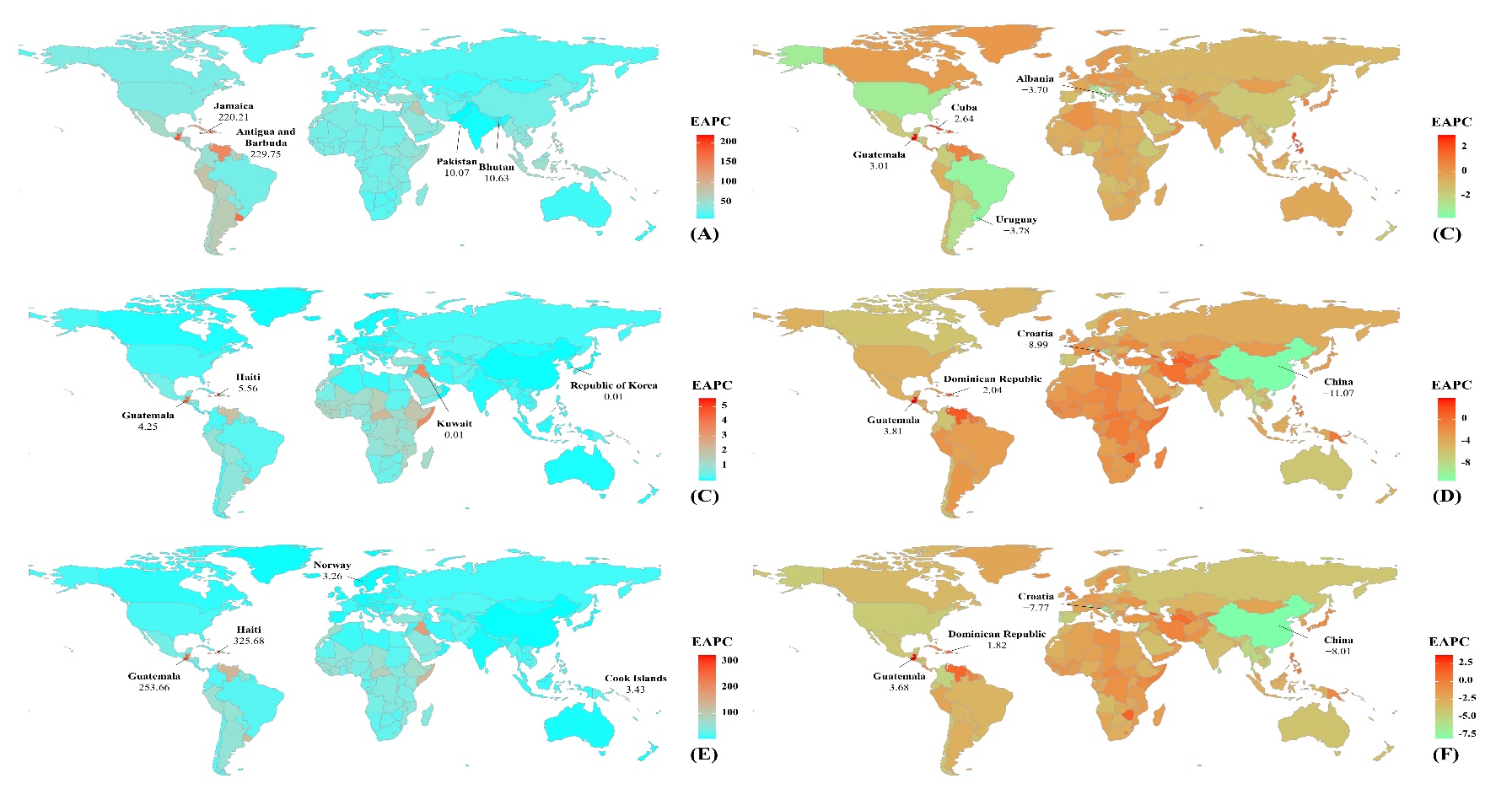
**

**Table S1**. The number and all-age rate of physical violence by firearm in 2019, and the percentage changes of number in age groups during the period 1990- 2019.

| **Age Groups** | **Incidence** | | | **Death** | | | **DALYs** | | |
| --- | --- | --- | --- | --- | --- | --- | --- | --- | --- |
|  | Number  ×10^3^(95% UI) | Rate/100,000  (95% UI) | Percent  (%) | Number  ×10^3^(95% UI) | Rate/100,000  (95% UI) | Percent (%) | Number  ×10^3^(95% UI) | Rate/100,000  (95% UI) | Percent (%) |
| **1–4** | 46.66  (21.24–82.68) | 8.79  (4–15.57) | 5.26 | 1.57  (1.11–2.08) | 0.3  (0.21–0.39) | 1.00 | 136.25  (97.4–180.24) | 25.67  (18.35–33.95) | 0.99 |
| **5–9** | 31.18  (11.9–63.42) | 4.76  (1.82–9.69) | 28.93 | 1.02  (0.85–1.21) | 0.16  (0.13–0.18) | -5.56 | 85.37  (71.88–100.71) | 13.04  (10.98–15.38) | -5.28 |
| **10–14** | 33.38  (12.77–69.18) | 5.2  (1.99–10.77) | 47.52 | 2.17  (1.92–2.44) | 0.34  (0.3–0.38) | 7.26 | 168.81  (149.52–190.01) | 26.29  (23.28–29.59) | 7.35 |
| **15–19** | 96.37  (51.63–171.41) | 15.56  (8.33–27.67) | 75.67 | 19.51  (17.7–21.45) | 3.15  (2.86–3.46) | 28.49 | 1396.7  (1266.84–1535.36) | 225.44  (204.48–247.82) | 28.39 |
| **20–24** | 135.15  (77.88–219.4) | 22.52  (12.98–36.56) | 88.02 | 31.17  (28.81–33.8) | 5.19  (4.8–5.63) | 31.15 | 2082.39  (1926.27–2257.97) | 346.98  (320.97–376.24) | 31.18 |
| **25–29** | 96.88  (50.66–165.59) | 16  (8.37–27.35) | 97.73 | 27.83  (25.87–30.13) | 4.6  (4.27–4.98) | 25.78 | 1726.7  (1605.38–1868.58) | 285.18  (265.15–308.62) | 25.95 |
| **30–34** | 60.24  (32.54–100.02) | 10.01  (5.41–16.62) | 103.3 | 23.65  (21.94–25.57) | 3.93  (3.65–4.25) | 31.83 | 1354.06  (1258.03–1465.95) | 225.03  (209.07–243.62) | 32.12 |
| **35–39** | 38.85  (20.2–69.18) | 7.18  (3.73–12.79) | 92.15 | 19.64  (18.07–21.5) | 3.63  (3.34–3.97) | 42.33 | 1030.1  (948.22–1125.53) | 190.42  (175.28–208.05) | 42.68 |
| **40–44** | 26.96  (13.59–49.27) | 5.46  (2.75–9.99) | 99.71 | 15.01  (13.66–16.43) | 3.04  (2.77–3.33) | 55.24 | 716.36  (652.1–783.13) | 145.18  (132.15–158.71) | 55.81 |
| **45–49** | 22.07  (10.83–41.61) | 4.66  (2.29–8.78) | 131.83 | 10.85  (9.83–12.07) | 2.29  (2.07–2.55) | 63.18 | 469.12  (425.11–521) | 99.01  (89.72–109.96) | 64.6 |
| **50–54** | 17.85  (8.83–32.13) | 4.09  (2.02–7.36) | 144.14 | 8.52  (7.75–9.39) | 1.95  (1.77–2.15) | 69.71 | 330.69  (301.65–362.15) | 75.7  (69.06–82.91) | 71.87 |
| **55–59** | 14.35  (6.83–27.53) | 3.87  (1.84–7.42) | 143.16 | 6.06  (5.5–6.67) | 1.63  (1.48–1.8) | 71.96 | 209.57  (191.97–229.57) | 56.49  (51.74–61.88) | 75.31 |
| **60–64** | 12.43  (5.54–24.88) | 3.98  (1.77–7.96) | 134.24 | 3.88  (3.57–4.27) | 1.24  (1.14–1.37) | 64.95 | 118.88  (109.44–129.97) | 38.04  (35.02–41.59) | 69.94 |
| **65–69** | 11.85  (5.16–22.64) | 4.58  (1.99–8.75) | 144.09 | 2.48  (2.27–2.71) | 0.96  (0.88–1.05) | 67.12 | 66.54  (61.17–72.88) | 25.73  (23.66–28.18) | 74.57 |
| **70–74** | 12.09  (5.59–22.14) | 6.46  (2.99–11.83) | 159.68 | 1.58  (1.46–1.71) | 0.84  (0.78–0.92) | 72.97 | 36.37  (33.26–39.67) | 19.44  (17.78–21.2) | 84.03 |
| **75–79** | 10.52  (5.02–19.5) | 8.28(  3.95–15.35) | 149.2 | 1.06  (0.97–1.16) | 0.84  (0.76–0.91) | 68.01 | 20.16  (18.28–22.15) | 15.87  (14.39–17.44) | 79.89 |
| **≥ 80** | 21.02  (12.46–34.58) | 14.06  (8.33–23.13) | 232.99 | 0.69  (0.58–0.76) | 0.46  (0.39–0.51) | 107.75 | 11.34  (9.71–13.23) | 7.59  (6.49–8.85) | 133.2 |

DALYs: disability adjusted life years; ASR, age-standardized rate; CI, confidence interval; UI: uncertainty interval.

**Table S2**. The number and all-age rate of self-harm by firearm in 2019, and the percentage changes of number in age groups during the period 1990- 2019.

| **Age Groups** | **Incidence** | | | **Death** | | | **DALYs** | | |
| --- | --- | --- | --- | --- | --- | --- | --- | --- | --- |
|  | Number  ×10^3^(95% UI) | Rate/100,000  (95% UI) | Percent  (%) | Number  ×10^3^(95% UI) | Rate/100,000  (95% UI) | Percent  (%) | Number  ×10^3^(95% UI) | Rate/100,000  (95% UI) | Percent  (%) |
| **10–14** | 1.11  (0.29–2.92) | 0.17  (0.05–0.45) | 15.13 | 0.65  (0.52–0.77) | 0.1  (0.08–0.12) | -3.4 | 49.99  (39.77–59.01) | 7.78  (6.19–9.19) | -3.43 |
| **15–19** | 4.7  (1.63–10.36) | 0.76  (0.26–1.67) | 17.48 | 2.52  (2.23–3.16) | 0.41  (0.36–0.51) | -32.44 | 180.31  (159.63–225.8) | 29.1  (25.77–36.45) | -32.44 |
| **20–24** | 11.95  (4.01–25.92) | 1.99  (0.67–4.32) | 22.24 | 4.25  (3.76–5.35) | 0.71  (0.63–0.89) | -28.11 | 284.63  (251.64–357.57) | 47.43  (41.93–59.58) | -28 |
| **25–29** | 20.43(  6.95–44.18) | 3.37  (1.15–7.3) | 31.65 | 4.3  (3.79–5.35) | 0.71  (0.63–0.88) | -23.68 | 268.29  (237.22–332.48) | 44.31  (39.18–54.91) | -23.34 |
| **30–34** | 28.61  (11.59–56.11) | 4.75  (1.93–9.33) | 39.07 | 4.16  (3.53–5.13) | 0.69  (0.59–0.85) | -20.8 | 240.41  (205.01–295.95) | 39.95  (34.07–49.18) | -20.15 |
| **35–39** | 32.23  (13.24–62.32) | 5.96  (2.45–11.52) | 22.43 | 4.02  (3.35–4.74) | 0.74  (0.62–0.88) | -12.9 | 213.56  (178.13–251.03) | 39.48  (32.93–46.4) | -12.17 |
| **40–44** | 38.72  (17.28–72.26) | 7.85  (3.5–14.64) | 37.02 | 4.01  (3.41–4.75) | 0.81  (0.69–0.96) | -1.14 | 193.3  (164.82–228.13) | 39.17  (33.4–46.23) | -0.21 |
| **45–49** | 45.27  (21.05–84.78) | 9.55  (4.44–17.89) | 70.27 | 4.3  (3.68–5.2) | 0.91  (0.78–1.1) | 19.17 | 186.8  (160.4–224.8) | 39.42  (33.85–47.45) | 20.31 |
| **50–54** | 40.44  (19.07–74.48) | 9.26  (4.37–17.05) | 62.47 | 4.33  (3.81–5.11) | 0.99  (0.87–1.17) | 35.7 | 167.74  (149.4–196.83) | 38.4  (34.2–45.06) | 36.46 |
| **55–59** | 35.62  (16.5–66.43) | 9.6  (4.45–17.91) | 73.53 | 4.48  (4.03–5.27) | 1.21  (1.09–1.42) | 50.92 | 152.1  (137.71–177.34) | 41  (37.12–47.8) | 51.25 |
| **60–64** | 28  (12.52–54.64) | 8.96  (4.01–17.48) | 67.86 | 3.86  (3.44–4.4) | 1.23  (1.1–1.41) | 38.28 | 114.22  (102.61–129.58) | 36.55  (32.83–41.46) | 39.25 |
| **65–69** | 20.73  (9.84–38.16) | 8.02  (3.81–14.76) | 94.05 | 3.32  (2.89–3.77) | 1.28  (1.12–1.46) | 28.86 | 83.88  (74.01–93.77) | 32.44  (28.62–36.26) | 31.81 |
| **70–74** | 11.19  (4.81–20.84) | 5.98  (2.57–11.14) | 100.93 | 2.76  (2.41–3.02) | 1.47  (1.29–1.62) | 33.13 | 57.27  (50.49–62.63) | 30.61  (26.99–33.48) | 36.67 |
| **75–79** | 7.44  (3.2–14.13) | 5.85  (2.52–11.12) | 73.7 | 2.24  (1.93–2.48) | 1.76  (1.52–1.95) | 12.73 | 36.97  (32.11–40.69) | 29.1  (25.27–32.02) | 16.25 |
| **≥ 80** | 8.81  (4.43–15.27) | 5.89  (2.96–10.21) | 159.42 | 3.51  (2.98–4) | 2.35  (1.99–2.67) | 60.78 | 38.19  (32.86–42.79) | 25.54  (21.97–28.62) | 58.29 |

DALYs: disability adjusted life years; ASR, age-standardized rate; CI, confidence interval; UI: uncertainty interval.

**Table S3**. The number and all-age rate of unintentional firearm injuries in 2019, and the percentage changes of number in age groups during the period 1990-2019.

| **Age Groups** | **Incidence** | | | **Death** | | | **DALYs** | | |
| --- | --- | --- | --- | --- | --- | --- | --- | --- | --- |
|  | Number  ×10^3^(95% UI) | Rate/100,000  (95% UI) | Percent  (%) | Number  ×10^3^(95% UI) | Rate/100,000  (95% UI) | Percent  (%) | Number  ×10^3^(95% UI) | Rate/100,000  (95% UI) | Percent  (%) |
| **1–4** | 229.71  (100.95–466.70) | 43.27  (19.02–87.91) | -14.6 | 0.94  (0.6–1.38) | 0.18  (0.11–0.26) | -51.65 | 84.75  (56.32–123.07) | 15.96  (10.61–23.18) | -50.65 |
| **5–9** | 179.64  (65.92–398.39) | 27.44  (10.07–60.85) | -7.51 | 0.52  (0.37–0.71) | 0.08  (0.06–0.11) | -49.33 | 49  (36.24–65.26) | 7.48  (5.54–9.97) | -46.17 |
| **10–14** | 159.21  (55.31–374.31) | 24.79  (8.61–58.29) | -6.43 | 0.54  (0.44–0.72) | 0.08  (0.07–0.11) | -50.69 | 50.64  (41.1–64.93) | 7.89  (6.4–10.11) | -46.13 |
| **15–19** | 170.7  (64.99–379.78) | 27.55  (10.49–61.3) | -11.08 | 1.54  (1.2–2.03) | 0.25  (0.19–0.33) | -37.08 | 122.19  (97.41–158.4) | 19.72  (15.72–25.57) | -35.06 |
| **20–24** | 175.74  (71.17–350.98) | 29.28  (11.86–58.48) | -8.48 | 2.19  (1.69–2.86) | 0.36  (0.28–0.48) | -34.38 | 161.15  (127.44–207.59) | 26.85  (21.24–34.59) | -32.38 |
| **25–29** | 165.01  (70.35–330.21) | 27.25  (11.62–54.54) | 1.85 | 2.07  (1.5–2.61) | 0.34  (0.25–0.43) | -23.76 | 146.61  (112.09–181.73) | 24.22  (18.51–30.02) | -21.1 |
| **30–34** | 157.54  (68.51–307.68) | 26.18  (11.39–51.13) | 17.06 | 1.81  (1.33–2.27) | 0.3  (0.22–0.38) | -21.87 | 125.43  (98.37–153.25) | 20.85  (16.35–25.47) | -17.2 |
| **35–39** | 144.72  (61.6–279.73) | 26.75  (11.39–51.71) | 21.4 | 1.59  (1.11–2.03) | 0.29  (0.2–0.37) | -22.54 | 107.46  (81.22–131.73) | 19.86  (15.01–24.35) | -15.91 |
| **40–44** | 128.86  (56.14–260.97) | 26.11  (11.38–52.89) | 41.84 | 1.44  (1.02–1.85) | 0.29  (0.21–0.37) | -12.31 | 93.43  (73.08–114.12) | 18.93  (14.81–23.13) | -3.3 |
| **45–49** | 119.12  (50.51–261.68) | 25.14  (10.66–55.23) | 73.22 | 1.32  (0.88–1.7) | 0.28  (0.19–0.36) | -7.86 | 82.92  (65.28–101.18) | 17.5  (13.78–21.36) | 6.18 |
| **50–54** | 102.01  (37.87–223.06) | 23.35  (8.67–51.06) | 80.47 | 1.01  (0.69–1.27) | 0.23  (0.16–0.29) | -16.95 | 64.74  (51.4–79.19) | 14.82  (11.77–18.13) | 3.46 |
| **55–59** | 78.01  (27.65–175.55) | 21.03  (7.45–47.32) | 76.52 | 0.83  (0.59–1.07) | 0.22  (0.16–0.29) | -11.95 | 51.99  (41.34–64.41) | 14.01  (11.14–17.36) | 11.44 |
| **60–64** | 59.57  (22.81–124.21) | 19.06  (7.3–39.74) | 76.24 | 0.73  (0.5–0.92) | 0.23  (0.16–0.29) | -8.16 | 42.14  (32.81–51.99) | 13.48  (10.5–16.63) | 16.47 |
| **65–69** | 45.82  (18.7–92.84) | 17.72  (7.23–35.9) | 91.8 | 0.61  (0.44–0.76) | 0.23  (0.17–0.29) | -3.96 | 32.39  (25.68–40.65) | 12.53  (9.93–15.72) | 26.2 |
| **70–74** | 36.45  (14.42–79.23) | 19.48  (7.71–42.35) | 95.86 | 0.51  (0.37–0.62) | 0.27  (0.2–0.33) | -1.48 | 23.23  (18.15–29.4) | 12.42  (9.7–15.71) | 33.35 |
| **75–79** | 29.22  (12.07–60.26) | 23  (9.5–47.43) | 78.97 | 0.5  (0.38–0.6) | 0.39  (0.3–0.47) | 9.4 | 16.93  (13.34–21.42) | 13.32  (10.5–16.86) | 37.22 |
| **≥ 80** | 60.18  (33.7–98.52) | 40.25  (22.53–65.89) | 148.12 | 0.94  (0.71–1.12) | 0.63  (0.48–0.75) | 57.88 | 20.77  (16.08–26.43) | 13.89  (10.76–17.67) | 86.32 |

DALYs: disability adjusted life years; ASR, age-standardized rate; CI, confidence interval; UI: uncertainty interval.

**Table S4**. Incident burden and trends of physical violence by firearm at the national level, 1990-2019.

|  | **1990** | | **2019** | | **1990-2019** | |
| --- | --- | --- | --- | --- | --- | --- |
| **Characteristics** | Number  ×10^2^ (95% UI) | ASR/100,000  (95% UI) | Number  ×10^2^ (95% UI) | ASR/100,000  (95% UI) | Percent (%) | EAPC  (95%CI) |
| Afghanistan | 3.44(2.36–4.85) | 2.81(2.1–3.62) | 20.29(12.65–30.67) | 4.19(2.85–5.86) | 489.19 | 1.17(0.9–1.44) |
| Albania | 4.09(3.03–5.43) | 11.44(8.66–15) | 1.76(1.26–2.37) | 7.2(5.16–9.99) | -57.02 | -3.84(-4.73–-2.94) |
| Algeria | 7.88(4.91–12.15) | 2.56(1.71–3.73) | 24.01(15.83–35.59) | 5.86(3.9–8.64) | 204.7 | 2.83(2.63–3.02) |
| American Samoa | 0.01(0.01–0.02) | 2.34(1.77–3.14) | 0.01(0.01–0.01) | 1.83(1.24–2.7) | -14.01 | -1.25(-1.42–-1.07) |
| Andorra | 0.03(0.02–0.05) | 6.06(3.98–9.48) | 0.05(0.04–0.08) | 8.11(5.3–12.33) | 75.42 | 1.09(0.95–1.22) |
| Angola | 0.93(0.58–1.47) | 0.81(0.57–1.13) | 3.65(2.15–5.81) | 1.02(0.7–1.49) | 292.88 | 0.73(0.49–0.98) |
| Antigua and Barbuda | 0.04(0.03–0.06) | 5.89(4.01–8.41) | 0.08(0.06–0.1) | 10.37(7.45–14.09) | 96.92 | 2.31(2.15–2.48) |
| Argentina | 34.97(26.83–44.72) | 10.54(8.11–13.43) | 79.19(60.24–101.52) | 17.92(13.67–23.08) | 126.49 | 1.05(0.75–1.36) |
| Armenia | 0.85(0.61–1.2) | 2.47(1.8–3.44) | 1.03(0.76–1.36) | 3.83(2.72–5.33) | 21.16 | 1.88(1.63–2.12) |
| Australia | 10.22(7.04–14.66) | 6.46(4.37–9.42) | 18.73(13.18–27.17) | 8.5(5.63–13.07) | 83.23 | 0.93(0.84–1.03) |
| Austria | 4.11(2.84–5.96) | 5.63(3.78–8.48) | 6.11(4.3–8.62) | 7.91(5.26–12.17) | 48.6 | 1.14(0.99–1.28) |
| Azerbaijan | 1.96(1.39–2.77) | 2.5(1.81–3.45) | 1.82(1.19–2.73) | 2.15(1.4–3.25) | -7.01 | -0.76(-1.41–-0.09) |
| Bahamas | 0.58(0.44–0.77) | 19.75(15.27–25.71) | 2.59(2.02–3.32) | 67.1(52.62–85.42) | 344.09 | 4.93(4.67–5.19) |
| Bahrain | 0.16(0.1–0.24) | 2.95(1.96–4.33) | 0.73(0.52–1.02) | 7.24(4.97–10.31) | 349.91 | 3.45(3.28–3.61) |
| Bangladesh | 6.7(4.52–9.89) | 0.72(0.54–0.99) | 14.68(9.91–22.27) | 1(0.7–1.46) | 119.02 | 0.9(0.53–1.27) |
| Barbados | 0.2(0.15–0.27) | 8(5.92–10.59) | 0.39(0.3–0.5) | 15.97(12.24–20.84) | 90.02 | 2.61(2.41–2.8) |
| Belarus | 2.3(1.65–3.18) | 2.39(1.66–3.37) | 3.08(2.21–4.27) | 3.76(2.55–5.55) | 33.5 | 1.72(1.48–1.97) |
| Belgium | 6.36(4.56–8.89) | 7.08(4.95–10.14) | 8.17(5.86–11.47) | 7.95(5.39–11.84) | 28.4 | 0.32(-0.04–0.68) |
| Belize | 0.14(0.1–0.19) | 6.36(4.84–8.25) | 1.33(1.04–1.69) | 28.99(22.8–36.26) | 842.95 | 6.09(5.62–6.56) |
| Benin | 1.01(0.64–1.53) | 1.77(1.28–2.37) | 3.91(2.44–6) | 2.56(1.82–3.57) | 288.57 | 1.32(1.24–1.41) |
| Bermuda | 0.04(0.03–0.06) | 8.39(5.82–11.94) | 0.07(0.05–0.09) | 15.51(11.17–21.04) | 57.67 | 2.5(2.32–2.69) |
| Bhutan | 0.04(0.02–0.06) | 0.73(0.52–1.04) | 0.08(0.05–0.12) | 1.21(0.8–1.82) | 115.25 | 1.92(1.62–2.22) |
| Bolivia | 2.33(1.73–3.08) | 3.6(2.78–4.65) | 8.53(6.12–11.68) | 6.54(4.8–8.82) | 266.48 | 1.99(1.73–2.25) |
| Bosnia and Herzegovina | 0.83(0.52–1.31) | 1.99(1.24–3.16) | 1.07(0.75–1.53) | 4.44(2.8–6.81) | 28.84 | 3.58(3.28–3.88) |
| Botswana | 0.25(0.16–0.38) | 1.75(1.19–2.48) | 1.38(0.99–1.86) | 5.83(4.28–7.74) | 451.4 | 4.28(3.71–4.84) |
| Brazil | 462.04(321.92–647.6) | 29.59(21.07–40.5) | 1413.76(969.24–1944.12) | 64.81(44.3–89.42) | 205.98 | 2.71(2.57–2.86) |
| Brunei Darussalam | 0.07(0.04–0.11) | 2.39(1.49–3.83) | 0.16(0.1–0.26) | 4.03(2.61–6.41) | 131.28 | 1.96(1.79–2.12) |
| Bulgaria | 3.54(2.64–4.75) | 4.84(3.51–6.52) | 2.38(1.71–3.32) | 4.73(3.17–7.11) | -32.71 | -0.43(-0.64–-0.22) |
| Burkina Faso | 2.74(1.88–3.99) | 3.01(2.31–3.87) | 9.52(6.21–13.95) | 3.82(2.83–5.16) | 247.34 | 0.78(0.73–0.84) |
| Burundi | 0.68(0.4–1.06) | 1.09(0.73–1.55) | 1.79(1.01–2.9) | 1.32(0.86–1.94) | 165.4 | 0.47(0.12–0.82) |
| Cabo Verde | 0.16(0.11–0.22) | 3.87(2.87–5.09) | 0.47(0.35–0.61) | 8.16(6.06–10.74) | 195.5 | 3.02(2.83–3.22) |
| Cambodia | 1.51(1.13–1.99) | 1.7(1.32–2.16) | 3.6(2.58–4.98) | 2.19(1.6–2.96) | 138.16 | 0.74(0.49–0.99) |
| Cameroon | 1.3(0.83–1.97) | 1.1(0.79–1.5) | 5(3.04–7.75) | 1.51(1.01–2.19) | 285.68 | 1.03(0.71–1.36) |
| Canada | 24.45(17.6–33.66) | 9.39(6.69–13.24) | 45.34(33.26–60.3) | 13.43(9.35–18.77) | 85.48 | 1.4(1.23–1.57) |
| Central African Republic | 0.33(0.22–0.48) | 1.14(0.85–1.53) | 1.2(0.9–1.61) | 2.36(1.83–3) | 264.47 | 2.77(2.01–3.53) |
| Chad | 1.18(0.78–1.76) | 1.67(1.24–2.24) | 4.05(2.56–6.1) | 2.2(1.61–2.94) | 242.72 | 0.89(0.82–0.97) |
| Chile | 7.01(4.92–9.77) | 5(3.55–6.89) | 24(18.16–31.23) | 14.5(10.74–19.38) | 242.37 | 4.29(4.01–4.56) |
| China | 650.97(433.57–952.35) | 5.55(3.68–8.16) | 765.57(531.45–1076.54) | 6.29(4.3–9.25) | 17.6 | -0.16(-0.56–0.24) |
| Colombia | 400.71(284.62–817.09) | 110.83(80.77–216.92) | 385.23(298.02–487.19) | 77.72(60.3–98.47) | -3.86 | -2.93(-4.18–-1.67) |
| Comoros | 0.08(0.06–0.11) | 1.78(1.35–2.3) | 0.19(0.14–0.26) | 2.85(2.12–3.83) | 132.03 | 1.65(1.58–1.73) |
| Congo | 0.27(0.18–0.41) | 1.12(0.81–1.51) | 0.63(0.41–0.94) | 1.19(0.84–1.67) | 131.71 | 0.18(-0.03–0.38) |
| Cook Islands | 0(0–0.01) | 2.09(1.47–2.94) | 0(0–0.01) | 2.74(1.8–4.07) | 13.24 | 0.62(0.44–0.79) |
| Costa Rica | 3.61(2.57–5) | 10.49(7.72–13.99) | 18.33(14.03–23.57) | 39.02(29.62–50.03) | 407.16 | 5.53(5.23–5.84) |
| Croatia | 4.06(3–5.48) | 9.3(6.74–12.69) | 2.04(1.43–2.84) | 6.38(4.13–9.55) | -49.82 | -1.94(-2.36–-1.51) |
| Cuba | 7.04(5.03–9.6) | 6.81(4.74–9.48) | 9.82(7.35–13.04) | 11.13(7.9–15.58) | 39.49 | 1.69(1.4–1.98) |
| Cyprus | 0.38(0.26–0.56) | 5.06(3.44–7.49) | 0.96(0.7–1.31) | 8.14(5.65–11.86) | 150.9 | 1.82(1.71–1.93) |
| Czechia | 4.11(2.78–6.08) | 4.72(3.09–6.99) | 6.51(4.51–9.21) | 8.1(5.19–12.33) | 58.21 | 1.77(1.62–1.92) |
| Côte d'Ivoire | 2.93(2.01–4.11) | 2.78(2.18–3.53) | 7.77(5.23–11.31) | 3.07(2.31–4.03) | 165.56 | 0.16(0.1–0.23) |
| DPR Korea | 9.43(6.38–13.47) | 3.79(2.61–5.35) | 8.27(6.01–11.6) | 3.69(2.61–5.24) | -12.27 | -0.25(-0.6–0.11) |
| DR Congo | 3.45(2.15–5.41) | 0.81(0.57–1.15) | 8.12(4.94–12.17) | 0.84(0.58–1.19) | 135.55 | -0.22(-0.49–0.06) |
| Denmark | 2.63(1.8–3.91) | 5.94(4.01–8.97) | 3.89(2.71–5.52) | 8.08(5.31–11.93) | 47.99 | 1.18(1.04–1.32) |
| Djibouti | 0.1(0.07–0.14) | 2.13(1.63–2.79) | 0.37(0.26–0.54) | 3.24(2.42–4.29) | 269.6 | 1.56(1.48–1.64) |
| Dominica | 0.04(0.03–0.06) | 5.16(3.64–7.11) | 0.06(0.04–0.08) | 9.73(7.02–13.27) | 42.15 | 2.66(2.42–2.9) |
| Dominican Republic | 5.69(4.28–7.53) | 7.06(5.51–9.04) | 34.38(26.02–44.85) | 29.94(23.11–38.48) | 504.33 | 5.41(5.2–5.61) |
| Ecuador | 8.48(6.66–10.66) | 8.9(7.14–10.93) | 23.86(18.19–30.76) | 13.19(10.18–16.94) | 181.46 | 1.6(0.55–2.65) |
| Egypt | 15.73(9.37–24.37) | 2.21(1.41–3.32) | 50.06(31.16–76.77) | 4.75(3.05–7.07) | 218.22 | 2.51(2.35–2.67) |
| El Salvador | 21.88(17.69–27.01) | 44.1(36.6–53) | 97.7(78.24–123.48) | 144.59(116.47–181.17) | 346.53 | 4.36(4.21–4.51) |
| Equatorial Guinea | 0.05(0.03–0.07) | 0.99(0.74–1.34) | 0.23(0.14–0.36) | 1.53(1.04–2.2) | 405.5 | 1.72(1.49–1.95) |
| Eritrea | 0.48(0.33–0.68) | 1.75(1.34–2.26) | 1.54(1.08–2.18) | 2.69(2.05–3.47) | 221.1 | 1.47(1.32–1.62) |
| Estonia | 1.23(0.95–1.57) | 8.55(6.55–10.94) | 0.49(0.34–0.68) | 4.26(2.81–6.48) | -60.56 | -3.24(-3.84–-2.63) |
| Eswatini | 0.38(0.28–0.52) | 4.74(3.71–6.05) | 0.48(0.35–0.65) | 4.05(3.09–5.37) | 25.53 | -0.88(-1.28–-0.48) |
| Ethiopia | 19.97(14.11–27.15) | 4.87(3.68–6.3) | 30.81(22.13–42.49) | 3.84(2.96–5.05) | 54.33 | -1.06(-1.2–-0.92) |
| Fiji | 0.1(0.06–0.15) | 1.26(0.85–1.86) | 0.13(0.09–0.2) | 1.57(1.07–2.28) | 37.53 | 0.77(0.59–0.96) |
| Finland | 2.87(2.08–3.93) | 6.58(4.6–9.47) | 3.79(2.68–5.28) | 7.85(5.23–11.89) | 32.08 | 0.4(0.26–0.54) |
| France | 32.03(22.29–45.55) | 5.93(4.07–8.69) | 45.19(31.54–63.22) | 7.54(5.01–11.23) | 41.09 | 0.8(0.71–0.89) |
| Gabon | 0.14(0.09–0.21) | 1.28(0.92–1.78) | 0.27(0.18–0.41) | 1.53(1.06–2.2) | 94.01 | 0.56(0.38–0.74) |
| Gambia | 0.14(0.07–0.22) | 1.04(0.67–1.53) | 0.42(0.23–0.71) | 1.55(0.95–2.45) | 213.21 | 1.49(1.05–1.92) |
| Georgia | 3.62(2.72–4.75) | 6.69(5.07–8.74) | 1.24(0.94–1.63) | 3.82(2.73–5.21) | -65.61 | -2.29(-2.85–-1.73) |
| Germany | 39.12(26.77–57.36) | 5.63(3.69–8.78) | 55.86(38.99–78.38) | 7.86(5.17–11.97) | 42.81 | 1.14(0.99–1.29) |
| Ghana | 2.3(1.43–3.47) | 1.27(0.89–1.77) | 6.79(4.12–10.41) | 1.94(1.26–2.86) | 195.08 | 1.23(1.06–1.4) |
| Greece | 6.02(4.11–8.81) | 6.43(4.37–9.56) | 6.52(4.66–8.93) | 7.3(4.85–11.1) | 8.18 | 0.48(0.4–0.57) |
| Greenland | 0.03(0.03–0.05) | 5.98(4.49–7.92) | 0.03(0.02–0.04) | 6.42(4.81–8.59) | -5.67 | -0.17(-0.47–0.12) |
| Grenada | 0.05(0.03–0.07) | 4.51(2.91–6.67) | 0.06(0.04–0.09) | 7.23(4.86–10.4) | 38.38 | 1.47(1.3–1.63) |
| Guam | 0.07(0.05–0.1) | 5.14(3.92–6.81) | 0.05(0.03–0.06) | 2.76(1.94–3.9) | -35.65 | -2.78(-3.1–-2.47) |
| Guatemala | 9.37(7.55–11.56) | 13.26(10.89–16.14) | 107.46(83.34–140.11) | 54.44(43.27–68.73) | 1046.78 | 6.93(6–7.86) |
| Guinea | 1.34(0.87–1.99) | 1.77(1.3–2.41) | 3.26(2.12–4.88) | 2.32(1.67–3.12) | 142.62 | 0.87(0.79–0.95) |
| Guinea–Bissau | 0.24(0.16–0.36) | 2.3(1.75–3.01) | 0.52(0.34–0.74) | 2.71(1.99–3.66) | 112.11 | 0.57(0.53–0.61) |
| Guyana | 0.44(0.33–0.57) | 5.16(3.97–6.55) | 1.16(0.9–1.48) | 14.15(11.15–17.69) | 164.01 | 3.76(3.14–4.37) |
| Haiti | 4.85(3.61–6.47) | 8.24(6.16–10.69) | 13.7(10–18.88) | 10.57(7.85–14.16) | 182.55 | 0.81(0.68–0.94) |
| Honduras | 8.63(6.71–10.97) | 20.84(16.51–25.9) | 62.41(48.25–80.7) | 58.67(46.14–74.33) | 622.98 | 3.92(3.62–4.23) |
| Hungary | 2.75(1.83–4.19) | 3.19(2.08–4.92) | 4.09(2.85–5.91) | 5.77(3.69–8.93) | 48.91 | 2.28(2.15–2.4) |
| Iceland | 0.15(0.1–0.24) | 6.17(4.08–9.43) | 0.25(0.17–0.36) | 7.92(5.23–11.88) | 59.07 | 0.98(0.84–1.13) |
| India | 85.58(58.76–124.17) | 1.3(0.95–1.76) | 140.96(98.6–204.83) | 1.15(0.82–1.63) | 64.7 | -0.6(-0.74–-0.47) |
| Indonesia | 23.48(14.79–36.5) | 1.34(0.89–1.94) | 44.83(28.75–68.68) | 1.87(1.22–2.84) | 90.94 | 1.33(0.94–1.72) |
| Iran | 33.72(20.98–51.4) | 4.4(2.95–6.41) | 50.73(33.98–74.09) | 6.8(4.53–9.91) | 50.45 | 1.67(1.49–1.86) |
| Iraq | 11.82(8.71–15.72) | 6.5(5.08–8.23) | 89.54(68.43–115.47) | 20.89(16.43–26.2) | 657.35 | 4.92(4.61–5.24) |
| Ireland | 1.8(1.17–2.81) | 5.06(3.33–7.72) | 3.22(2.19–4.73) | 7.47(4.87–11.39) | 79.06 | 1.44(1.35–1.54) |
| Israel | 4.09(2.84–5.78) | 7.99(5.63–11.24) | 13.18(9.8–17.68) | 14.51(10.73–19.77) | 222.47 | 1.75(1.05–2.46) |
| Italy | 86.52(55.45–126.16) | 15.94(10.26–23.24) | 52.92(36.73–72.58) | 10.57(6.95–15.77) | -38.83 | -1.78(-2.22–-1.35) |
| Jamaica | 4.99(3.98–6.2) | 19.96(16.18–24.36) | 7.08(5.46–8.84) | 24.73(19.02–30.52) | 41.89 | 0.92(0.08–1.77) |
| Japan | 47.35(30.96–73.46) | 4.29(2.75–6.73) | 67.38(46.92–93.67) | 6.16(3.96–9.66) | 42.31 | 1.41(1.18–1.64) |
| Jordan | 1.94(1.29–2.84) | 4.14(2.99–5.7) | 11.55(8.41–15.64) | 10.08(7.65–13.17) | 496.75 | 3.49(3.33–3.66) |
| Kazakhstan | 4.45(3.23–6.07) | 2.62(1.92–3.55) | 5.63(4.05–7.92) | 3.26(2.33–4.59) | 26.33 | 1.21(0.96–1.47) |
| Kenya | 7.71(5.21–11.19) | 3.87(2.93–5.05) | 16.88(12.03–23.39) | 4.21(3.16–5.47) | 118.8 | 0.47(0.36–0.58) |
| Kiribati | 0.01(0.01–0.01) | 1.27(0.91–1.77) | 0.01(0.01–0.02) | 1.18(0.83–1.68) | 57.06 | -0.36(-0.78–0.06) |
| Kuwait | 1.05(0.7–1.54) | 5.56(3.8–7.94) | 3.22(2.18–4.61) | 9.48(6.29–13.82) | 208.17 | 1.8(1.66–1.93) |
| Kyrgyzstan | 1.41(1.01–1.95) | 2.82(2.09–3.79) | 1.87(1.21–2.82) | 2.76(1.84–4.12) | 32.8 | -0.38(-0.62–-0.15) |
| Lao People's Democratic Republic | 1.4(1.06–1.82) | 4.13(3.16–5.33) | 2.15(1.61–2.8) | 3.22(2.49–4.09) | 53.28 | -1.22(-1.42–-1.03) |
| Latvia | 1(0.76–1.29) | 4.08(3.03–5.36) | 0.61(0.44–0.84) | 3.7(2.51–5.58) | -38.78 | -0.44(-0.81–-0.07) |
| Lebanon | 4.68(3.4–6.33) | 13.08(9.79–17.31) | 7.69(5.74–10.11) | 15.24(11.21–20.25) | 64.17 | 0.11(-1.09–1.33) |
| Lesotho | 0.75(0.56–0.99) | 4.18(3.29–5.35) | 1.04(0.81–1.35) | 5.11(3.99–6.49) | 39.49 | 0.43(0.23–0.64) |
| Liberia | 0.27(0.17–0.4) | 1.18(0.85–1.63) | 0.65(0.39–1.01) | 1.26(0.83–1.85) | 143.95 | 0.26(-0.15–0.67) |
| Libya | 1.61(1.01–2.49) | 3.01(2.05–4.38) | 11.01(8.26–14.44) | 19.32(14.31–25.26) | 582.72 | 8.79(7.44–10.16) |
| Lithuania | 1.64(1.23–2.12) | 4.63(3.45–6.11) | 0.85(0.61–1.18) | 3.61(2.43–5.48) | -48.1 | -1.28(-1.73–-0.84) |
| Luxembourg | 0.19(0.13–0.27) | 5.64(3.84–8.38) | 0.46(0.33–0.67) | 8.69(5.78–13.04) | 145.64 | 1.45(1.35–1.55) |
| Madagascar | 1.32(0.78–2.09) | 1.03(0.69–1.47) | 3.17(1.9–5.04) | 1.14(0.76–1.66) | 141.12 | 0.31(-0.01–0.64) |
| Malawi | 0.88(0.52–1.35) | 0.83(0.58–1.17) | 2.19(1.28–3.51) | 1.12(0.75–1.64) | 149.85 | 1.25(1.05–1.46) |
| Malaysia | 3.4(2.2–5.2) | 1.88(1.3–2.7) | 10.36(7.25–14.72) | 3.53(2.47–4.96) | 205.03 | 2.35(2.2–2.51) |
| Maldives | 0.04(0.03–0.06) | 1.87(1.32–2.6) | 0.26(0.17–0.37) | 5.24(3.71–7.37) | 552.8 | 4.18(3.85–4.51) |
| Mali | 2.2(1.45–3.26) | 2.15(1.57–2.91) | 7.57(4.73–11.51) | 2.73(1.93–3.82) | 244.52 | 0.75(0.59–0.92) |
| Malta | 0.25(0.18–0.36) | 7.41(5.14–10.67) | 0.34(0.25–0.46) | 8.99(6.23–13.17) | 34.94 | 0.46(0.37–0.55) |
| Marshall Islands | 0.01(0.01–0.01) | 1.87(1.39–2.45) | 0.01(0.01–0.01) | 1.74(1.25–2.43) | 21.74 | -0.48(-0.73–-0.24) |
| Mauritania | 0.37(0.25–0.55) | 1.71(1.27–2.25) | 1.21(0.78–1.79) | 2.78(1.93–3.91) | 223.1 | 1.67(1.54–1.8) |
| Mauritius | 0.23(0.15–0.34) | 2.12(1.43–3.02) | 0.35(0.25–0.48) | 2.98(2.11–4.18) | 52.13 | 1.17(0.93–1.42) |
| Mexico | 127.15(89.54–180.77) | 14.82(10.91–19.83) | 355.92(251.75–491.97) | 27.99(20–38.54) | 179.92 | 2.73(2.29–3.16) |
| Micronesia | 0.02(0.01–0.02) | 1.93(1.48–2.48) | 0.02(0.01–0.03) | 2.05(1.49–2.82) | 14.57 | -0.05(-0.16–0.06) |
| Monaco | 0.01(0.01–0.02) | 5.1(3.21–7.97) | 0.02(0.01–0.03) | 6.58(4.23–10.14) | 61.38 | 1.01(0.85–1.16) |
| Mongolia | 0.26(0.17–0.39) | 1.12(0.79–1.55) | 0.61(0.4–0.92) | 1.85(1.23–2.79) | 138.6 | 2.29(1.92–2.66) |
| Montenegro | 0.36(0.26–0.5) | 6.02(4.33–8.36) | 0.36(0.26–0.49) | 6.87(4.76–9.89) | -1.56 | 0.46(0.4–0.53) |
| Morocco | 5.37(3.18–8.33) | 1.78(1.14–2.65) | 16.01(10.3–23.68) | 4.84(3.11–7.2) | 198.31 | 3.43(3.26–3.61) |
| Mozambique | 1.69(1.13–2.52) | 1.15(0.85–1.57) | 6.3(4.03–9.36) | 1.93(1.4–2.58) | 272.77 | 2.18(1.98–2.39) |
| Myanmar | 6.41(4.72–8.51) | 1.7(1.29–2.18) | 9.18(6.51–12.85) | 1.74(1.25–2.41) | 43.11 | -0.2(-0.68–0.27) |
| Namibia | 0.51(0.37–0.67) | 3.87(3.02–4.9) | 1.2(0.85–1.65) | 4.75(3.52–6.34) | 136.88 | 0.43(0.19–0.66) |
| Nauru | 0(0–0) | 2.21(1.65–2.96) | 0(0–0) | 2.31(1.69–3.19) | 5.21 | -0.18(-0.38–0.03) |
| Nepal | 1.77(1.17–2.73) | 1.04(0.74–1.51) | 4.72(3.08–7.36) | 1.67(1.15–2.5) | 166.69 | 1.72(1.62–1.82) |
| Netherlands | 8.83(6.05–12.87) | 6.35(4.3–9.54) | 11.2(7.69–16.02) | 7.67(5.07–11.69) | 26.81 | 0.07(-0.29–0.43) |
| New Zealand | 2(1.27–3.32) | 5.97(3.73–9.95) | 2.34(1.54–3.62) | 5.52(3.46–9.17) | 17.03 | -0.24(-0.51–0.03) |
| Nicaragua | 3.26(2.39–4.38) | 7.5(5.84–9.51) | 9.3(6.75–12.5) | 13.85(10.32–18.3) | 185.68 | 2.55(2.35–2.74) |
| Niger | 1.84(1.19–2.73) | 2.05(1.51–2.73) | 7.07(4.36–10.95) | 2.38(1.68–3.28) | 284.42 | 0.43(0.26–0.61) |
| Nigeria | 36.8(22.78–55.41) | 3.34(2.32–4.72) | 89.75(55.47–137.74) | 3.66(2.53–5.18) | 143.89 | 0.34(0.28–0.4) |
| Niue | 0(0–0) | 2.38(1.73–3.24) | 0(0–0) | 2.74(1.95–3.81) | -23.04 | 0.28(0.19–0.37) |
| North Macedonia | 0.56(0.38–0.81) | 2.84(1.93–4.13) | 1.01(0.72–1.38) | 5.6(3.8–8.07) | 80.53 | 2.4(1.91–2.89) |
| Northern Mariana Islands | 0.03(0.02–0.03) | 5.83(4.45–7.42) | 0.01(0.01–0.02) | 3.8(2.78–5.15) | -47.97 | -2.5(-2.94–-2.06) |
| Norway | 2.08(1.43–3.05) | 5.32(3.52–7.99) | 3.22(2.18–4.66) | 6.88(4.42–10.71) | 54.31 | 0.77(0.46–1.09) |
| Oman | 0.8(0.5–1.24) | 3.33(2.18–4.99) | 3.85(2.53–5.67) | 8.79(5.85–12.78) | 380.82 | 3.25(3.08–3.43) |
| Pakistan | 19.93(14.13–27.91) | 2.53(1.84–3.45) | 34.1(23.5–49.13) | 2.16(1.57–2.94) | 71.15 | -0.61(-0.67–-0.54) |
| Palau | 0(0–0) | 2.06(1.49–2.82) | 0(0–0) | 2.62(1.92–3.56) | 21.62 | 0.61(0.49–0.72) |
| Palestine | 1.14(0.77–1.65) | 4.32(3.14–5.87) | 12.8(9.66–16.9) | 25.44(20.01–32.16) | 1019.5 | 7.1(6.73–7.48) |
| Panama | 3.61(2.7–4.72) | 13.19(10.04–17) | 21.49(16.31–28.03) | 50.77(38.57–65.99) | 496.04 | 5.73(5.29–6.17) |
| Papua New Guinea | 0.64(0.43–0.98) | 1.72(1.21–2.41) | 2.53(1.71–3.78) | 2.87(2.03–4.06) | 297.11 | 1.9(1.66–2.13) |
| Paraguay | 5.71(4.37–7.44) | 14.27(11.13–18.17) | 16.76(12.12–22.55) | 22.75(16.76–30.39) | 193.75 | 0.88(0.55–1.2) |
| Peru | 8.04(5.81–11.06) | 3.33(2.5–4.43) | 23.96(17.32–32.74) | 7.16(5.14–9.74) | 198.12 | 2.93(2.72–3.15) |
| Philippines | 121.48(86.52–162.79) | 22.86(16.84–30.14) | 227.39(166.42–302.64) | 21.97(16.36–28.44) | 87.19 | -0.49(-0.66–-0.31) |
| Poland | 10.96(7–17.35) | 3.12(1.97–4.95) | 14.39(9.6–21.87) | 5.19(3.17–8.52) | 31.29 | 2.13(1.79–2.48) |
| Portugal | 5.57(3.99–7.63) | 6.02(4.32–8.31) | 7.14(5.19–9.72) | 7.83(5.41–11.53) | 28.1 | 0.63(0.51–0.75) |
| Puerto Rico | 33.49(25.57–44.11) | 89.33(68.7–117.67) | 37.26(29.11–48.21) | 122.05(93.89–158.5) | 11.23 | 1.83(1.36–2.3) |
| Qatar | 0.16(0.1–0.24) | 3.52(2.27–5.26) | 2.08(1.35–3.06) | 9.16(6.31–13.02) | 1210.86 | 3.88(3.62–4.14) |
| Republic of Korea | 13.38(8.37–22.06) | 2.95(1.89–4.64) | 27.67(19.12–39.72) | 6.3(4.11–9.8) | 106.8 | 2.56(2.36–2.75) |
| Republic of Moldova | 1.16(0.84–1.55) | 2.72(1.96–3.64) | 0.94(0.67–1.33) | 3.43(2.24–5.13) | -18.96 | 0.74(0.34–1.13) |
| Romania | 5.74(3.67–8.75) | 2.77(1.74–4.17) | 8.48(5.92–12.39) | 6.15(3.96–9.51) | 47.6 | 2.75(2.64–2.87) |
| Russian Federation | 55.35(37.92–79.97) | 3.97(2.67–5.82) | 44.61(31.5–64.05) | 3.58(2.37–5.36) | -19.4 | -0.54(-0.74–-0.35) |
| Rwanda | 1.01(0.68–1.49) | 1.3(0.98–1.73) | 2.1(1.3–3.19) | 1.56(1.06–2.26) | 107.75 | 0.53(-0.03–1.1) |
| Saint Kitts and Nevis | 0.07(0.05–0.09) | 15.08(11.63–19.55) | 0.36(0.28–0.47) | 63.55(48.52–82.97) | 448.55 | 6.26(5.84–6.68) |
| Saint Lucia | 0.11(0.08–0.15) | 7.3(5.58–9.39) | 0.42(0.32–0.54) | 24.88(19.01–32.44) | 273.36 | 4.88(4.3–5.46) |
| Saint Vincent and the Grenadines | 0.11(0.08–0.14) | 8.49(6.53–10.93) | 0.2(0.15–0.26) | 18.73(14.24–23.89) | 91.64 | 3.41(3.13–3.68) |
| Samoa | 0.03(0.02–0.05) | 2.17(1.6–2.9) | 0.05(0.03–0.07) | 2.3(1.65–3.25) | 40.9 | -0.12(-0.26–0.02) |
| San Marino | 0.01(0.01–0.02) | 5.67(3.62–8.83) | 0.02(0.01–0.03) | 7.17(4.59–11.03) | 67.01 | 0.96(0.82–1.09) |
| Sao Tome and Principe | 0.02(0.01–0.04) | 1.62(1.11–2.29) | 0.06(0.04–0.09) | 2.82(1.84–4.15) | 151.83 | 1.97(1.66–2.28) |
| Saudi Arabia | 6.04(3.52–9.37) | 3.02(1.93–4.45) | 18.38(12.37–26.83) | 6.86(4.44–10.11) | 204.28 | 2.77(2.59–2.95) |
| Senegal | 0.85(0.48–1.36) | 0.9(0.59–1.32) | 2.58(1.45–4.27) | 1.44(0.88–2.22) | 202.56 | 1.76(1.4–2.13) |
| Serbia | 4.53(3.32–6.09) | 5.31(3.86–7.17) | 4.08(2.93–5.66) | 5.64(3.87–8.34) | -9.94 | -0.1(-0.21–0.02) |
| Seychelles | 0.02(0.02–0.03) | 3.34(2.54–4.33) | 0.05(0.04–0.06) | 4.98(3.76–6.46) | 112.34 | 1.15(1.06–1.23) |
| Sierra Leone | 0.37(0.23–0.57) | 0.88(0.6–1.25) | 1.06(0.62–1.69) | 1.14(0.75–1.69) | 188.29 | 0.87(0.39–1.34) |
| Singapore | 1.23(0.79–1.93) | 4.18(2.72–6.41) | 2.9(1.92–4.24) | 6.52(4.15–10.39) | 135.72 | 1.61(1.5–1.71) |
| Slovakia | 1.7(1.17–2.48) | 3.42(2.33–5.04) | 2.14(1.51–3.09) | 5.1(3.34–7.85) | 26.08 | 1.22(1.07–1.37) |
| Slovenia | 0.78(0.55–1.1) | 4.67(3.16–6.78) | 0.95(0.66–1.36) | 6.05(3.81–9.42) | 21.98 | 0.84(0.7–0.97) |
| Solomon Islands | 0.07(0.05–0.1) | 2.27(1.63–3.1) | 0.14(0.1–0.21) | 2.19(1.54–3.1) | 113.11 | -0.51(-0.64–-0.39) |
| Somalia | 1.32(0.94–1.82) | 2.08(1.63–2.66) | 4.65(3.27–6.51) | 2.7(2.1–3.46) | 252.73 | 1.09(0.82–1.36) |
| South Africa | 51.79(36.74–71.43) | 14.91(10.84–20.33) | 39.68(28.16–54.29) | 7.4(5.31–10.1) | -23.39 | -2.71(-2.87–-2.54) |
| South Sudan | 1.02(0.7–1.47) | 1.88(1.42–2.45) | 2.62(1.81–3.65) | 2.99(2.29–3.89) | 156.09 | 1.77(1.68–1.85) |
| Spain | 17.44(11.42–27.29) | 4.94(3.19–7.65) | 25.64(17.48–36.19) | 6.88(4.41–10.47) | 47.09 | 1.22(1.09–1.36) |
| Sri Lanka | 5.86(4.34–7.97) | 3.3(2.51–4.36) | 9.92(7.33–13.05) | 4.76(3.53–6.39) | 69.12 | 0.94(0.78–1.1) |
| Sudan | 3.63(2.05–5.83) | 1.38(0.88–2.06) | 17.29(11.32–25.17) | 3.61(2.52–5.09) | 376.69 | 3.25(3.14–3.36) |
| Suriname | 0.18(0.13–0.25) | 4.28(3.14–5.81) | 0.42(0.31–0.57) | 7.93(5.73–10.89) | 131.56 | 2.31(2.12–2.51) |
| Sweden | 4.38(3.02–6.31) | 5.75(3.75–8.74) | 6.74(4.72–9.42) | 7.5(4.98–11.29) | 53.83 | 0.94(0.6–1.29) |
| Switzerland | 4.37(3.04–6.21) | 6.86(4.68–10.18) | 6.13(4.23–8.64) | 8.1(5.25–12.4) | 40.37 | 0.51(0.33–0.68) |
| Syrian Arab Republic | 4.01(2.56–6.1) | 2.43(1.68–3.44) | 9.2(6.4–12.8) | 6.85(4.93–9.41) | 129.12 | 3.95(3.59–4.32) |
| Taiwan  (Province of China) | 10.24(7.3–14.15) | 5.17(3.71–7.12) | 15.04(10.96–20.09) | 7.93(5.49–11.05) | 46.96 | 1.63(1.5–1.76) |
| Tajikistan | 0.97(0.65–1.4) | 1.63(1.19–2.23) | 1.66(0.98–2.68) | 1.62(1.02–2.54) | 71.6 | -0.2(-0.62–0.22) |
| Thailand | 66.73(49.43–87.38) | 11.4(8.64–14.55) | 56.23(43.72–71.44) | 8.04(6.2–10.4) | -15.73 | -1.86(-2.07–-1.66) |
| Timor–Leste | 0.11(0.08–0.14) | 1.58(1.19–2.03) | 0.27(0.19–0.37) | 2.04(1.51–2.71) | 147.27 | 0.99(0.73–1.25) |
| Togo | 0.7(0.45–1.06) | 1.75(1.28–2.36) | 1.99(1.29–2.86) | 2.41(1.71–3.26) | 183.34 | 1.13(1–1.26) |
| Tokelau | 0(0–0) | 1.17(0.84–1.65) | 0(0–0) | 1.87(1.25–2.75) | 30.41 | 1.54(1.31–1.77) |
| Tonga | 0.02(0.01–0.03) | 2.26(1.69–3.02) | 0.02(0.01–0.03) | 1.85(1.23–2.83) | -3.31 | -1.21(-1.67–-0.76) |
| Trinidad and Tobago | 1.11(0.83–1.45) | 8.6(6.5–11.08) | 5.67(4.31–7.28) | 45.42(33.96–59.33) | 410.12 | 7.71(6.56–8.87) |
| Tunisia | 2.73(1.69–4.18) | 2.75(1.78–4.07) | 6.77(4.55–9.64) | 6.8(4.48–9.8) | 148.08 | 3.21(3.03–3.4) |
| Turkey | 27.95(20–38.39) | 4.47(3.31–5.98) | 60.65(44.89–81.21) | 8.54(6.15–11.86) | 116.98 | 2.21(2.08–2.34) |
| Turkmenistan | 0.91(0.67–1.25) | 2.34(1.8–3.1) | 1.5(1.04–2.15) | 2.93(2.07–4.16) | 63.78 | 0.77(0.43–1.11) |
| Tuvalu | 0(0–0) | 1.47(1.11–1.93) | 0(0–0) | 1.83(1.31–2.53) | 56.18 | 0.38(0.27–0.49) |
| Uganda | 2.68(1.74–3.96) | 1.39(1.04–1.88) | 9.52(5.97–14.23) | 1.95(1.38–2.69) | 255.54 | 0.97(0.84–1.11) |
| Ukraine | 13.35(9.05–19.63) | 2.93(1.95–4.33) | 11.56(8.12–16.05) | 3.22(2.16–4.82) | -13.41 | 0.16(-0.05–0.37) |
| United Arab Emirates | 0.59(0.36–0.89) | 2.97(1.93–4.41) | 3.16(2.1–4.6) | 6.35(4.04–9.48) | 438.38 | 2.44(2.25–2.63) |
| United Kingdom | 26.2(17.71–39.24) | 5.18(3.32–8.03) | 42.03(28.47–61.09) | 7.35(4.72–11.6) | 60.4 | 1.36(1.01–1.71) |
| United Republic of Tanzania | 5.31(3.38–7.91) | 1.77(1.3–2.38) | 16.82(10.59–25.72) | 2.42(1.69–3.46) | 216.98 | 1.1(0.97–1.24) |
| United States of America | 963.62(651.2–1380.73) | 37.89(25.28–54.69) | 1180.42(796.42–1658.34) | 38.95(25.89–55.89) | 22.5 | 0(-0.11–0.12) |
| United States Virgin Islands | 0.5(0.39–0.66) | 48.32(37.16–63.71) | 0.65(0.51–0.81) | 73.02(55.29–94.11) | 28.62 | 1.39(0.7–2.08) |
| Uruguay | 3.02(2.26–3.94) | 10.18(7.55–13.31) | 8.85(6.82–11.5) | 27.95(21.24–36.65) | 192.91 | 3.31(3.06–3.56) |
| Uzbekistan | 4.02(2.6–6) | 1.63(1.13–2.37) | 9.9(6.29–15.52) | 2.88(1.87–4.45) | 146.42 | 2.32(2.02–2.61) |
| Vanuatu | 0.02(0.01–0.03) | 1.2(0.82–1.73) | 0.04(0.03–0.07) | 1.42(0.95–2.11) | 131.71 | 0.45(0.13–0.78) |
| Venezuela | 59.37(44.23–76.89) | 27.9(21.18–35.55) | 400.25(305.63–510.89) | 147.41(112.29–189.26) | 574.16 | 5.7(4.63–6.78) |
| Viet Nam | 12.76(8.48–19.41) | 1.92(1.34–2.73) | 40.88(28.34–59.43) | 4.48(3.1–6.52) | 220.38 | 3.25(2.97–3.53) |
| Yemen | 2.78(1.74–4.3) | 1.64(1.18–2.27) | 14.04(9.18–20.34) | 3.74(2.59–5.24) | 405.3 | 3.48(3.27–3.69) |
| Zambia | 1.27(0.84–1.89) | 1.44(1.08–1.92) | 5(3.39–7.18) | 3.09(2.33–4.11) | 295.48 | 3.15(2.97–3.32) |
| Zimbabwe | 2.53(1.58–3.88) | 2.08(1.48–2.88) | 2.9(1.9–4.29) | 2.08(1.48–2.88) | 14.48 | -0.79(-1.29–-0.28) |

EAPC: estimated annual percentage change; ASR, age-standardized rate; CI, confidence interval; UI: uncertainty interval.

**Table S5**. Global burden and trends in death and DALYs due to physical violence by firearm in sexes, SDI areas, and regions, 1990-2019.

| **Characteristics** | **Death** | | | | **DALYs** | | | |  |
| --- | --- | --- | --- | --- | --- | --- | --- | --- | --- |
|  | 2019 | | 1990-2019 | | 2019 | | 1990-2019 | | |
|  | Number  ×10^3^ (95% UI) | ASR/100,000  (95% UI) | % | EAPC  (95%CI) | Number  ×10^3^ (95% UI) | ASR/100,000  (95% UI) | % | EAPC  (95%CI) | |
| **Overall** | 177.47  (163.86–192.65) | 2.23  (2.06–2.43) | 37.60 | -0.49  (-0.64–-0.33) | 10028.58  (9276.49–10915.01) | 127.56  (117.85–138.91) | 34.46 | -0.40  (-0.56–-0.24) | |
| **Sex** |  |  |  |  |  |  |  |  | |
| Male | 158.79  (146.66–172.8) | 3.97  (3.67–4.31) | 41.54 | -0.35  (-0.50–-0.20) | 8970.17  (8286.46–9731.81) | 225.51  (208.31–245.21) | 38.68 | -0.26  (-0.41–-0.11) | |
| Female | 18.68  (17.04–20.73) | 0.48  (0.43–0.53) | 11.27 | -1.47  (-1.71–-1.23) | 1058.41  (961.01–1177.87) | 27.61  (25.04–30.77) | 6.92 | -1.36  (-1.59–-1.13) | |
| **SDI** |  |  |  |  |  |  |  |  | |
| Low | 19.09  (14.92–23.02) | 1.95  (1.55–2.34) | -0.38 | -0.62  (-0.74–-0.51) | 1108.82  (858.71–1345.74) | 101.83  (79.71–122.42) | 103.94 | -0.52  (-0.64–-0.41) | |
| Low-middle | 55.68  (49.83–62.14) | 3.05  (2.73–3.41) | 144.69 | 1.75  (1.42–2.07) | 3166.46  (2834.34–3531.35) | 168.68  (150.82–188.17) | 141.07 | 1.91  (1.56–2.26) | |
| Middle | 70.03  (63.32–77.54) | 2.76  (2.50–3.05) | 23.25 | -1.20  (-1.48–-0.92) | 3894.81  (3535.73–4301.08) | 155.64  (141.23–171.88) | 18.93 | -1.09  (-1.37–-0.81) | |
| High-middle | 17.61  (16.63–18.65) | 1.23  (1.16–1.31) | -14.72 | -2.13  (-2.81–-1.44) | 1001.74  (945.54–1058.90) | 73.67  (69.47–78.08) | -16.45 | -1.96  (-2.65–-1.27) | |
| High | 14.88  (13.95–15.38) | 1.55  (1.45–1.60) | 56.90 | -1.40  (-1.73–-1.08) | 846.62  (792.2–876.20) | 93.20  (87.41–96.44) | -24.49 | -1.42  (-1.74–-1.09) | |
| **Regions** |  |  |  |  |  |  |  |  | |
| East Asia | 0.40  (0.34–0.48) | 0.03  (0.02–0.03) | -57.44 | -4.48  (-4.81–-4.16) | 37.51  (30.87–45.85) | 2.37  (1.98–2.84) | -44.66 | -3.62  (-3.95–-3.29) | |
| South Asia | 10.96  (8.80–13.63) | 0.61  (0.49–0.75) | 37.13 | -1.67  (-1.89–-1.44) | 572.93  (457.06–711.62) | 30.28  (24.29–37.51) | 34.25 | -1.51  (-1.75–-1.28) | |
| Southeast Asia | 12.59  (10.17–15.58) | 1.73  (1.4–2.14) | -14.79 | -2.87  (-3.12–-2.61) | 658.53  (538.51–808.48) | 90.78  (74.46–111.29) | -21.74 | -3.01  (-3.29–-2.74) | |
| Central Asia | 0.31  (0.27–0.36) | 0.33  (0.29–0.38) | -49.75 | -5.26  (-5.82–-4.70) | 17.22  (15.07–19.77) | 17.51  (15.30–20.06) | -51.26 | -5.46  (-6.02–-4.89) | |
| High-income Asia Pacific | 0.04  (0.04–0.04) | 0.02  (0.02–0.02) | -47.89 | -3.53  (-3.85–-3.21) | 4.57  (3.62–5.74) | 2.17  (1.75–2.66) | -16.53 | -1.40  (-1.61–-1.19) | |
| Oceania | 0.12  (0.05–0.17) | 1.04  (0.45–1.47) | 161.44 | 1.21  (0.68–1.75) | 6.62  (2.58–9.27) | 50.94  (20.52–71.28) | 149.74 | 0.93  (0.44–1.42) | |
| Australasia | 0.05  (0.05–0.06) | 0.17  (0.16–0.19) | -54.44 | -4.29  (-5.14–-3.44) | 3.21  (2.94–3.50) | 11.06  (10.2–12.03) | -50.09 | -3.88  (-4.65–-3.09) | |
| Eastern Europe | 1.51  (1.30–1.74) | 0.67  (0.58–0.77) | -37.96 | -3.92  (-4.90–-2.93) | 77.00  (66.61–88.11) | 36.20  (31.39–41.36) | -43.32 | -4.09  (-5.06–-3.11) | |
| Western Europe | 0.99  (0.95–1.04) | 0.21  (0.2–0.22) | -60.75 | -4.07  (-4.29–-3.84) | 54.86  (51.41–59.25) | 12.79  (12.05–13.7) | -59.99 | -3.85  (-4.09–-3.61) | |
| Central Europe | 0.31  (0.25–0.37) | 0.25  (0.20–0.30) | -55.48 | -5.20  (-6.02–-4.37) | 16.08  (13.36–19.16) | 14.31  (11.95–17.11) | -57.23 | -5.11  (-5.94–-4.28) | |
| High-income North America | 13.65  (12.73–14.1) | 3.90  (3.63–4.03) | -20.73 | -1.50  (-1.87–-1.14) | 771.65  (717.06–799.15) | 230.94  (214.78–239.04) | -23.59 | -1.59  (-1.95–-1.23) | |
| Andean Latin America | 1.81  (1.41–2.25) | 2.76  (2.16–3.44) | 31.22 | -1.36  (-2.17–-0.55) | 99.95  (77.79–125.16) | 150.49  (117.18–187.98) | 26.96 | -1.31  (-2.14–-0.47) | |
| Central Latin America | 52.88  (44.62–62.32) | 20.09  (16.95–23.68) | 51.48 | -0.58  (-0.88–-0.28) | 2968.12  (2510.67–3488.19) | 1122.25  (949.35–1319.25) | 46.26 | -0.48  (-0.77–-0.19) | |
| Caribbean | 4.22  (2.96–5.41) | 8.68  (6.07–11.14) | 91.07 | 1.59  (1.25–1.93) | 239.55  (167.09–307) | 496.24  (345.36–635.66) | 83.95 | 1.57  (1.25–1.89) | |
| Tropical Latin America | 48.02  (46.08–50.22) | 20.34  (19.51–21.28) | 98.51 | 0.83  (0.46–1.19) | 2794.09  (2678.02–2919.52) | 1200.52  (1149.99–1255.51) | 96.13 | 1.08  (0.71–1.44) | |
| Southern Latin America | 2.19  (2.05–2.33) | 3.15  (2.95–3.35) | 34.32 | -0.32  (-0.82–0.19) | 121.83  (113.8–129.83) | 179.2  (167.11–191.63) | 37.11 | -0.12  (-0.62–0.39) | |
| Eastern sub-Saharan Africa | 8.64  (6.84–10.58) | 2.58  (2.06–3.11) | 126.34 | -0.13  (-0.27–0.01) | 492.86  (387.12–606.04) | 128.27  (101.5–156.52) | 122.78 | -0.12  (-0.26–0.02) | |
| Southern sub-Saharan Africa | 3.38  (2.77–4.38) | 4.12  (3.43–5.28) | -42.03 | -4.86  (-5.48–-4.24) | 188.20  (152.99–246.85) | 220.66  (180.77–287.54) | -42.88 | -4.85  (-5.46–-4.23) | |
| Western sub-Saharan Africa | 8.18  (5.92–10.56) | 2.04  (1.50–2.60) | 145.37 | -0.41  (-0.71–-0.12) | 493.01  (354.69–640.19) | 108.64  (78.9–140) | 147.44 | -0.36  (-0.67–-0.05) | |
| North Africa  and Middle East | 6.54  (5.30–8.40) | 1.04  (0.84–1.32) | 67.52 | 0.04  (-0.43–0.52) | 371.09  (301.39–469.83) | 57.28  (46.58–72.21) | 61.07 | 0.10  (-0.39–0.59) | |
| Central sub-Saharan Africa | 0.67  (0.45–1.22) | 0.57  (0.38–1.06) | 104.45 | -0.63  (-0.85–-0.40) | 39.70  (26.28–70.66) | 30.98  (20.67–56.71) | 100.08 | -0.63  (-0.86–-0.40) | |

DALYs: disability adjusted life years; EAPC: estimated annual percentage change; ASR, age-standardized rate; CI, confidence interval; UI: uncertainty interval; SDI: socio-demographic index.

**Table S6**. Burden and trends in death and DALYs caused by physical violence by firearm at the national level,1990-2019.

|  | **Death** | | | **DALYs** | | |
| --- | --- | --- | --- | --- | --- | --- |
| **Characteristics** | ASR/100,000  (95% UI) | Percent  (%) | EAPC  (95%CI) | ASR/100,000  (95% UI) | Percent (%) | EAPC  (95%CI) |
| Afghanistan | 1.32(0.67–2.75) | 111.65 | -1.82(-2.83–-0.8) | 73.11(36.49–151.31) | 117.6 | -1.99(-2.87–-1.1) |
| Albania | 1.39(1–1.84) | -60.63 | -6.7(-8.48–-4.88) | 76.28(55.55–99.84) | -64.03 | -6.66(-8.45–-4.83) |
| Algeria | 0.31(0.21–0.48) | 13.48 | -2.23(-2.91–-1.54) | 18.74(13.15–28.5) | 12.34 | -2.02(-2.68–-1.36) |
| American Samoa | 0.44(0.28–0.65) | -63.16 | -5.55(-6–-5.09) | 22.31(13.96–34.65) | -65 | -5.54(-6.01–-5.07) |
| Andorra | 0.08(0.05–0.12) | -24.38 | -2.51(-2.75–-2.28) | 6.22(4.31–8.43) | -10.23 | -1.78(-2–-1.56) |
| Angola | 0.61(0.37–1.11) | 111.92 | -1(-1.09–-0.91) | 33.74(20.2–60.27) | 108.93 | -0.99(-1.09–-0.89) |
| Antigua and Barbuda | 1.9(1.58–2.24) | 144.1 | 1.94(1.31–2.58) | 113.58(94.91–134.46) | 136.45 | 1.89(1.27–2.51) |
| Argentina | 3.64(3.36–3.92) | 32.78 | -0.63(-1.2–-0.06) | 204.68(187.38–222.18) | 35.91 | -0.48(-1.04–0.09) |
| Armenia | 0.64(0.53–0.77) | -27.73 | -1.75(-2.56–-0.93) | 33.77(27.81–40.49) | -34.74 | -1.88(-2.72–-1.03) |
| Australia | 0.18(0.16–0.19) | -51.88 | -4.36(-5.29–-3.42) | 11.36(10.39–12.42) | -46.84 | -3.91(-4.75–-3.06) |
| Austria | 0.13(0.12–0.14) | -61.92 | -4.58(-5.37–-3.79) | 8.16(7.39–9.02) | -58.8 | -4.08(-4.82–-3.34) |
| Azerbaijan | 0.27(0.18–0.46) | -56.79 | -7.6(-9.3–-5.87) | 15.06(9.73–26.45) | -58.61 | -8.01(-9.82–-6.17) |
| Bahamas | 19.72(16.07–24.36) | 182.02 | 3.64(2.97–4.3) | 1123.61(919.41–1382.06) | 172.48 | 3.74(3.07–4.42) |
| Bahrain | 0.36(0.23–0.56) | 179.23 | -0.76(-1.1–-0.42) | 20.09(13.3–30.78) | 166.72 | -0.5(-0.81–-0.2) |
| Bangladesh | 0.78(0.57–1.14) | 92.35 | -1.02(-1.99–-0.05) | 37.69(27.79–56.27) | 75.36 | -0.82(-1.72–0.09) |
| Barbados | 4.3(3.56–5.24) | 54.28 | 1.47(1.02–1.93) | 248.97(206.68–303.87) | 42.99 | 1.34(0.9–1.79) |
| Belarus | 0.43(0.32–0.57) | -20.73 | -2.29(-3.15–-1.43) | 23.26(17.39–30.34) | -29.79 | -2.57(-3.39–-1.74) |
| Belgium | 0.34(0.31–0.36) | -49.29 | -4.22(-4.84–-3.59) | 19.06(17.54–20.7) | -49.87 | -4.14(-4.75–-3.54) |
| Belize | 15.04(13.13–17.13) | 876.98 | 5.34(4.51–6.18) | 858.31(752.48–973.42) | 837.62 | 5.38(4.53–6.24) |
| Benin | 2.57(1.75–3.68) | 259.43 | 0.75(0.56–0.93) | 134.96(90.14–195.24) | 252.56 | 0.77(0.62–0.93) |
| Bermuda | 1.5(1.23–1.81) | -7.3 | 0.44(-0.18–1.06) | 86.25(70.81–104.23) | -14.58 | 0.42(-0.16–1.01) |
| Bhutan | 0.1(0.04–0.2) | -22.89 | -2.7(-2.99–-2.41) | 5.63(2.6–11.03) | -23.94 | -2.53(-2.83–-2.24) |
| Bolivia | 2.56(1.65–3.53) | 46.87 | -1.21(-1.28–-1.14) | 140.57(90.8–192.56) | 41.46 | -1.22(-1.29–-1.15) |
| Bosnia and Herzegovina | 0.26(0.18–0.37) | -43.68 | -1.02(-1.24–-0.8) | 14.49(10.43–21.65) | -46.81 | -0.79(-1.01–-0.57) |
| Botswana | 3.48(1.93–5.71) | 1450.45 | 7.23(4.72–9.8) | 191.86(102.68–312.32) | 1360.44 | 7.2(4.71–9.75) |
| Brazil | 20.8(19.96–21.74) | 98.03 | 0.86(0.5–1.22) | 1229.55(1179.1–1284.31) | 95.72 | 1.12(0.76–1.48) |
| Brunei Darussalam | 0.07(0.05–0.09) | 20.08 | -1.51(-1.88–-1.14) | 4.59(3.5–5.75) | 22.13 | -1.39(-1.73–-1.05) |
| Bulgaria | 0.55(0.43–0.69) | -27.38 | -3.67(-4.85–-2.48) | 30.29(24.07–37.54) | -34.85 | -3.8(-4.94–-2.64) |
| Burkina Faso | 3.91(2.87–5.11) | 175.71 | 0.12(-0.13–0.36) | 187.5(133.44–250.79) | 187.51 | 0.3(0.04–0.57) |
| Burundi | 0.28(0.16–0.47) | 140.27 | 0.23(-0.1–0.55) | 16.45(9.1–26.85) | 135.27 | 0.25(-0.06–0.56) |
| Cabo Verde | 5.78(4.22–8.01) | 105.54 | 0.56(0.34–0.78) | 324.32(232.8–449.64) | 92.94 | 0.48(0.26–0.71) |
| Cambodia | 0.95(0.64–1.5) | 30.39 | -1.57(-1.78–-1.37) | 51.76(34.21–81.69) | 23.28 | -1.63(-1.84–-1.42) |
| Cameroon | 1.03(0.64–1.48) | 195.07 | -0.75(-1.02–-0.48) | 58.23(36.29–84.59) | 197.92 | -0.64(-0.91–-0.37) |
| Canada | 0.5(0.45–0.55) | -19.79 | -0.79(-1.33–-0.24) | 31.46(28.25–35.04) | -14.7 | -0.53(-1.05–-0.01) |
| Central African Republic | 1.27(0.78–2.76) | 80.6 | 0.23(-0.19–0.65) | 66.63(40.06–143.59) | 80.4 | 0.28(-0.16–0.72) |
| Chad | 2.99(2.06–4.08) | 303.78 | 1.25(0.71–1.8) | 154.68(107.41–212.26) | 307.89 | 1.22(0.68–1.77) |
| Chile | 1.82(1.66–1.99) | 40.62 | 0.62(0.2–1.04) | 106.32(96.17–116.79) | 39.73 | 0.86(0.45–1.26) |
| China | 0.02(0.02–0.03) | -58.96 | -4.62(-4.91–-4.32) | 2.2(1.82–2.68) | -44.05 | -3.64(-3.96–-3.32) |
| Colombia | 24.8(19.4–31.92) | -34.44 | -4.26(-4.92–-3.6) | 1427.4(1125.57–1826.54) | -36.25 | -4.16(-4.8–-3.52) |
| Comoros | 3.16(1.79–4.77) | 191.99 | 0.96(0.38–1.55) | 157.24(85.28–241.43) | 163.63 | 0.72(0.09–1.36) |
| Congo | 0.89(0.52–1.73) | 59.33 | -1.6(-1.76–-1.44) | 46.23(26.73–89.45) | 51.81 | -1.62(-1.79–-1.45) |
| Cook Islands | 0.16(0.1–0.26) | -62.76 | -3.84(-4.1–-3.58) | 8.62(4.98–13.52) | -65.62 | -3.72(-3.98–-3.47) |
| Costa Rica | 6.94(5.24–8.8) | 331.68 | 4.09(3.6–4.58) | 394.92(300.53–498.43) | 324.36 | 4.25(3.8–4.7) |
| Croatia | 0.34(0.27–0.43) | -75.76 | -7.63(-8.52–-6.73) | 18.78(15.1–23.21) | -77.07 | -7.56(-8.44–-6.67) |
| Cuba | 0.28(0.22–0.35) | -68.28 | -4.95(-5.75–-4.16) | 18.46(14.83–22.71) | -68.52 | -4.75(-5.52–-3.97) |
| Cyprus | 0.51(0.41–0.64) | 18.04 | -1.92(-2.04–-1.79) | 25.77(20.86–32.37) | 21.35 | -1.69(-1.81–-1.56) |
| Czechia | 0.15(0.12–0.18) | -32.97 | -4.44(-5.55–-3.31) | 9.48(7.92–11.31) | -29.93 | -3.96(-4.98–-2.93) |
| Côte d'Ivoire | 3.28(2.22–4.67) | 121.23 | -0.58(-0.84–-0.31) | 158.91(104.99–226.15) | 120.08 | -0.45(-0.73–-0.17) |
| DPR Korea | 0.12(0.07–0.19) | 2.59 | -1.13(-1.24–-1.01) | 6.77(4.41–10.09) | -12.42 | -1.3(-1.4–-1.2) |
| DR Congo | 0.49(0.29–0.89) | 116.21 | -0.39(-0.76–-0.02) | 26.47(15.36–47.81) | 109.86 | -0.41(-0.79–-0.03) |
| Denmark | 0.15(0.13–0.16) | -48.35 | -3.64(-4.26–-3.02) | 10.18(9.13–11.38) | -42.82 | -3.21(-3.76–-2.67) |
| Djibouti | 3.39(1.99–5.19) | 297.16 | 1.15(0.56–1.74) | 168.58(97.19–264.63) | 257.2 | 0.96(0.38–1.54) |
| Dominica | 3.53(2.45–4.71) | 71.16 | 2.68(2.22–3.15) | 206.46(144.16–275.61) | 62.58 | 2.67(2.18–3.16) |
| Dominican Republic | 11.28(7.88–15.46) | 194.85 | 3.56(3.03–4.09) | 620.95(438.08–842.11) | 181.35 | 3.46(2.97–3.95) |
| Ecuador | 5.5(4.13–7.2) | 38.54 | -1.33(-2.7–0.05) | 294.3(220.16–383.13) | 34.27 | -1.3(-2.71–0.14) |
| Egypt | 0.38(0.26–0.54) | 132.2 | 2.22(1.62–2.82) | 20.63(14.54–28.72) | 122.52 | 2.24(1.64–2.83) |
| El Salvador | 35.5(26.66–46.6) | 32.85 | 0.8(-0.12–1.73) | 1946.86(1474.45–2540.61) | 29.38 | 0.98(0.05–1.92) |
| Equatorial Guinea | 0.68(0.33–1.38) | 90.27 | -2.65(-2.98–-2.32) | 35.73(17–73.07) | 94.37 | -2.63(-2.94–-2.31) |
| Eritrea | 4.11(2.56–6.18) | 311.06 | 1.72(1.32–2.12) | 207.18(128.03–320.15) | 288.98 | 1.59(1.22–1.96) |
| Estonia | 0.27(0.2–0.34) | -90.49 | -11.77(-13.27–-10.25) | 15.39(12.03–19.34) | -90.89 | -11.7(-13.15–-10.23) |
| Eswatini | 3.66(2.19–5.73) | 2.63 | -1.66(-1.84–-1.48) | 204.17(119.93–321.78) | 1.58 | -1.49(-1.67–-1.32) |
| Ethiopia | 2.91(2.16–3.69) | 34.88 | -1.96(-2.08–-1.84) | 132.8(99.68–169.38) | 33.63 | -2.02(-2.15–-1.9) |
| Fiji | 0.16(0.11–0.21) | 33.01 | 0.14(-0.28–0.56) | 8.16(5.88–11.28) | 26.22 | 0.06(-0.31–0.44) |
| Finland | 0.21(0.2–0.24) | -68 | -4.84(-5.33–-4.34) | 13.2(12.12–14.61) | -65.7 | -4.49(-4.95–-4.03) |
| France | 0.32(0.29–0.35) | -45.77 | -2.97(-3.27–-2.67) | 17.8(16.39–19.62) | -47.62 | -2.88(-3.21–-2.55) |
| Gabon | 0.8(0.4–1.62) | 44.25 | -1.19(-1.28–-1.1) | 41.64(21.14–83.72) | 40.91 | -1.19(-1.28–-1.1) |
| Gambia | 0.24(0.15–0.32) | 224.25 | 0.37(-0.18–0.93) | 13.33(8.44–18.14) | 220.72 | 0.48(-0.06–1.03) |
| Georgia | 1.2(0.94–1.46) | -74.95 | -4.36(-5.8–-2.89) | 63.32(49.77–77.05) | -76.83 | -4.51(-5.93–-3.06) |
| Germany | 0.08(0.08–0.09) | -54.9 | -4.83(-5.43–-4.24) | 5.85(5.21–6.55) | -48.27 | -4.05(-4.62–-3.49) |
| Ghana | 0.73(0.5–0.99) | 101.96 | -0.45(-1.09–0.19) | 40.59(27.27–55.36) | 94.41 | -0.49(-1.07–0.09) |
| Greece | 0.35(0.32–0.38) | -32.85 | -1.79(-2.52–-1.05) | 19.01(17.43–20.7) | -36.3 | -1.68(-2.39–-0.97) |
| Greenland | 2.06(1.51–2.75) | -51.35 | -3.02(-3.34–-2.71) | 112.22(80.97–152.12) | -55.62 | -3.17(-3.51–-2.82) |
| Grenada | 0.66(0.54–0.79) | 130.92 | 2.67(1.91–3.42) | 40.9(33.57–48.5) | 123.51 | 2.63(1.91–3.35) |
| Guam | 0.58(0.42–0.78) | -64.95 | -4.9(-5.17–-4.64) | 30.81(22.14–42.05) | -67.3 | -4.87(-5.12–-4.61) |
| Guatemala | 28.23(22.18–35.37) | 262.63 | 2.28(0.75–3.84) | 1551.76(1219.05–1928.1) | 268.79 | 2.33(0.8–3.87) |
| Guinea | 2.65(1.83–3.72) | 174.2 | 1.16(0.93–1.39) | 138.68(95.14–197.33) | 171.18 | 1.08(0.87–1.29) |
| Guinea–Bissau | 3.43(2.42–4.67) | 124.23 | 0.46(0.24–0.68) | 173.33(121.77–238.03) | 116.82 | 0.41(0.2–0.63) |
| Guyana | 10.22(7.87–12.94) | 139.64 | 3.4(2.3–4.51) | 564.31(435.8–714.14) | 124.45 | 3.22(2.12–4.34) |
| Haiti | 9.79(1.8–15.95) | 112.03 | 0.04(-0.06–0.14) | 559.37(109.02–894.74) | 110.26 | 0.08(-0.03–0.19) |
| Honduras | 21.22(14.09–30.42) | 103.98 | 0.99(-0.35–2.36) | 1095.61(715.75–1572.96) | 96.38 | 0.84(-0.54–2.24) |
| Hungary | 0.07(0.06–0.09) | -66.38 | -6.01(-6.83–-5.18) | 5.05(4.25–5.99) | -59.9 | -5.16(-5.9–-4.4) |
| Iceland | 0.06(0.05–0.06) | -44.03 | -3.59(-4.15–-3.03) | 4.72(4.05–5.43) | -28.06 | -2.57(-2.91–-2.23) |
| India | 0.56(0.44–0.7) | 27.16 | -1.85(-2.14–-1.57) | 28.07(22.22–35.26) | 24.2 | -1.73(-2.03–-1.44) |
| Indonesia | 0.06(0.05–0.08) | -13.8 | -2.97(-4.32–-1.6) | 3.45(2.84–4.69) | -17.12 | -2.89(-4.22–-1.55) |
| Iran | 0.6(0.51–0.77) | 77.48 | 0.79(0.62–0.97) | 36.05(31.37–46.08) | 67.14 | 0.98(0.8–1.17) |
| Iraq | 6.57(4.71–8.88) | 497.52 | 3.23(2.46–4.01) | 337.79(241.28–457.55) | 447.65 | 2.95(2.19–3.72) |
| Ireland | 0.18(0.16–0.2) | 55.32 | -0.13(-1.25–1) | 11.29(10.1–12.59) | 58.01 | 0.1(-0.95–1.15) |
| Israel | 1.05(0.96–1.15) | 113.55 | 0.57(-0.48–1.64) | 59.75(54.5–65.43) | 106.28 | 0.49(-0.53–1.52) |
| Italy | 0.35(0.33–0.38) | -81.24 | -6(-6.51–-5.49) | 20.25(18.91–22.04) | -81.7 | -5.94(-6.46–-5.42) |
| Jamaica | 10.06(7.76–12.85) | 99.04 | -0.03(-1.58–1.54) | 525.72(409.11–669.07) | 86.24 | -0.13(-1.63–1.39) |
| Japan | 0.02(0.02–0.02) | -52.37 | -3.42(-3.76–-3.08) | 2(1.62–2.49) | -24.73 | -1.29(-1.55–-1.03) |
| Jordan | 1.51(1.17–1.92) | 274.61 | 0.35(-0.12–0.83) | 83.01(64.64–104.57) | 261.48 | 0.31(-0.16–0.79) |
| Kazakhstan | 0.56(0.47–0.67) | -16.74 | -2.57(-3.39–-1.74) | 30.51(25.38–36.38) | -24.11 | -2.84(-3.66–-2.02) |
| Kenya | 3.75(2.61–5.01) | 213.72 | 0.92(0.66–1.18) | 174.85(121.51–234.95) | 201.33 | 0.93(0.66–1.2) |
| Kiribati | 0.39(0.17–0.59) | -41.24 | -1.47(-2.46–-0.48) | 21.22(8.97–32.2) | -41.81 | -1.42(-2.39–-0.43) |
| Kuwait | 0.28(0.22–0.35) | 12.5 | -6.21(-8.03–-4.35) | 15.38(12.36–18.91) | 8.55 | -6.06(-7.77–-4.32) |
| Kyrgyzstan | 0.4(0.34–0.47) | -61.17 | -6.02(-6.71–-5.32) | 21.51(18.45–25.2) | -61.9 | -6.04(-6.76–-5.32) |
| Lao People's Democratic Republic | 2.47(1.45–3.91) | -2.69 | -2.68(-3.16–-2.19) | 125.33(72.22–198.87) | -6.79 | -2.74(-3.21–-2.26) |
| Latvia | 0.42(0.34–0.53) | -78.74 | -7.81(-9.1–-6.5) | 22.74(18.56–28.24) | -80.3 | -7.89(-9.17–-6.6) |
| Lebanon | 1.3(0.77–2.36) | -91.18 | -2.41(-4.59–-0.18) | 77.78(46.55–138.38) | -91.28 | -2.22(-4.43–0.03) |
| Lesotho | 5.3(3.32–8.18) | 84.98 | -0.02(-0.48–0.45) | 289.26(178.34–451.7) | 82.74 | 0.07(-0.36–0.51) |
| Liberia | 0.84(0.55–1.19) | -2.77 | -6.9(-10.17–-3.52) | 47.48(30.29–67.95) | -3.63 | -6.86(-10.12–-3.49) |
| Libya | 0.59(0.4–0.9) | 104.44 | 7.15(3.61–10.82) | 36.65(26–53.72) | 112.09 | 7.41(3.98–10.97) |
| Lithuania | 0.3(0.24–0.36) | -84.13 | -7.86(-8.85–-6.85) | 16.93(13.99–20.36) | -85.21 | -7.9(-8.87–-6.91) |
| Luxembourg | 0.21(0.18–0.24) | -29.67 | -3.84(-4.21–-3.47) | 12.87(11.35–14.62) | -22.68 | -3.47(-3.85–-3.08) |
| Madagascar | 0.6(0.4–0.83) | 249.69 | 1.8(0.5–3.13) | 34.46(22.69–48.74) | 237.53 | 1.79(0.49–3.1) |
| Malawi | 0.62(0.39–0.99) | 115.22 | 0.67(-0.23–1.59) | 35.47(21.7–57.4) | 107.77 | 0.59(-0.24–1.43) |
| Malaysia | 0.35(0.24–0.52) | 140.24 | 0.89(0.43–1.35) | 19.58(13.47–28.85) | 129.08 | 0.67(0.31–1.04) |
| Maldives | 0.15(0.1–0.2) | 199.18 | -0.18(-0.63–0.27) | 9.22(6.62–12.23) | 198.46 | 0.08(-0.35–0.52) |
| Mali | 4.45(2.98–6.07) | 275.84 | 0.32(-0.16–0.79) | 242.19(164.63–331.04) | 279.72 | 0.35(-0.14–0.85) |
| Malta | 0.45(0.4–0.51) | -11.68 | -1.55(-1.78–-1.33) | 26.44(23.43–29.54) | -12.68 | -1.38(-1.59–-1.16) |
| Marshall Islands | 1.07(0.65–1.7) | -17.36 | -2.08(-2.27–-1.89) | 57.25(34.22–92.45) | -22.04 | -2.1(-2.31–-1.89) |
| Mauritania | 1.83(1.02–2.82) | 77.67 | -0.41(-0.58–-0.23) | 92.56(50.29–143.6) | 74.22 | -0.42(-0.58–-0.27) |
| Mauritius | 0.17(0.14–0.22) | 167.62 | 3.91(0.98–6.93) | 8.89(7.17–10.93) | 127.3 | 3.47(0.87–6.15) |
| Mexico | 15.55(13.15–18.28) | 130.88 | 1.23(0.1–2.37) | 843.91(717.56–986.97) | 124.26 | 1.42(0.25–2.6) |
| Micronesia | 1.23(0.63–1.99) | -18.08 | -1.54(-1.67–-1.41) | 64.1(31.35–104.92) | -21.74 | -1.56(-1.69–-1.43) |
| Monaco | 0.09(0.06–0.13) | 13.9 | 0.16(-0.04–0.35) | 6.12(4.33–8.46) | 20.41 | 0.26(0.1–0.42) |
| Mongolia | 0.22(0.13–0.43) | 21.37 | -1.93(-2.43–-1.43) | 13.14(7.42–25.96) | 17.44 | -1.88(-2.38–-1.38) |
| Montenegro | 0.99(0.77–1.25) | -31.66 | -1.88(-2.23–-1.52) | 52.25(40.73–65.81) | -37.3 | -1.95(-2.31–-1.59) |
| Morocco | 0.46(0.23–0.88) | 196.72 | 1.93(0.79–3.09) | 25.19(12.77–49.05) | 172.36 | 1.86(0.7–3.03) |
| Mozambique | 2.36(1.57–3.32) | 262.61 | 2.77(2.25–3.28) | 127.81(84.26–178.81) | 254.6 | 2.57(2.08–3.07) |
| Myanmar | 0.38(0.25–0.57) | -57.26 | -4.02(-4.86–-3.17) | 21.13(14.01–32.08) | -59.03 | -4.05(-4.89–-3.2) |
| Namibia | 3.37(2.01–5.16) | 46.01 | -1.41(-1.8–-1.01) | 182.85(103.98–285.95) | 45.99 | -1.29(-1.7–-0.89) |
| Nauru | 1.18(0.69–2.22) | -18.55 | -1.19(-1.52–-0.85) | 61.15(34.92–119.05) | -18.85 | -1.25(-1.57–-0.92) |
| Nepal | 0.1(0.07–0.14) | 108.8 | 0.6(0.26–0.95) | 5.99(4.03–8.32) | 90.43 | 0.45(0.11–0.8) |
| Netherlands | 0.23(0.21–0.25) | -32.62 | -2.89(-3.58–-2.19) | 14.23(13.1–15.61) | -30.48 | -2.7(-3.35–-2.04) |
| New Zealand | 0.15(0.14–0.16) | -66.58 | -3.97(-4.87–-3.05) | 9.38(8.76–10.11) | -65.04 | -3.74(-4.58–-2.89) |
| Nicaragua | 2.9(2.17–3.98) | 11.02 | -2.07(-2.46–-1.68) | 149.95(110.92–208.53) | 2.32 | -2.25(-2.63–-1.86) |
| Niger | 2.83(1.32–4.49) | 167.12 | -0.38(-0.54–-0.23) | 150.52(71.08–240.2) | 165.79 | -0.36(-0.53–-0.2) |
| Nigeria | 1.81(1.1–2.63) | 113.43 | -0.55(-1.06–-0.03) | 98.49(59.81–143.46) | 117.63 | -0.48(-1.01–0.04) |
| Niue | 0.63(0.36–1.06) | -56.15 | -2.72(-2.92–-2.52) | 33.29(18.96–56.85) | -59.34 | -2.8(-3–-2.59) |
| North Macedonia | 0.98(0.74–1.25) | 18.16 | -0.58(-1.66–0.52) | 50.76(39.02–64.33) | 6.26 | -0.78(-1.88–0.34) |
| Northern Mariana Islands | 0.97(0.64–1.41) | -66.57 | -3.83(-4.2–-3.46) | 49.21(31.85–73.13) | -70.3 | -3.75(-4.11–-3.39) |
| Norway | 0.07(0.07–0.08) | -79.37 | -6.71(-7.62–-5.79) | 5.12(4.68–5.69) | -74.37 | -5.98(-6.78–-5.18) |
| Oman | 0.03(0.02–0.04) | 128.23 | -0.58(-0.74–-0.41) | 3.18(2.45–3.96) | 245.25 | 0.81(0.71–0.9) |
| Pakistan | 0.93(0.53–1.5) | 61.68 | -1.25(-1.66–-0.84) | 44.03(25.63–70.64) | 68.35 | -1.11(-1.53–-0.69) |
| Palau | 0.78(0.51–1.15) | 3.5 | -0.57(-0.66–-0.49) | 40.64(26.38–59.8) | -4.5 | -0.49(-0.58–-0.39) |
| Palestine | 5.01(4.12–6.18) | 839.08 | 7.45(6.38–8.52) | 264.91(217.58–328.34) | 785.91 | 7.21(6.17–8.26) |
| Panama | 10.6(7.98–13.58) | 238.73 | 3.69(2.68–4.7) | 626.77(477–799.36) | 228.81 | 3.7(2.7–4.72) |
| Papua New Guinea | 1.16(0.4–1.72) | 335.91 | 2.98(2.15–3.81) | 55.03(16.68–80.63) | 311.83 | 2.58(1.81–3.37) |
| Paraguay | 7.11(5.14–9.51) | 155.56 | -0.53(-1.43–0.38) | 379.95(275.94–506.97) | 147.1 | -0.56(-1.48–0.38) |
| Peru | 1.44(0.97–1.99) | 12.52 | -1.86(-2.44–-1.27) | 80.04(54.43–110.66) | 8.99 | -1.78(-2.33–-1.23) |
| Philippines | 8.28(6.41–10.56) | 27.85 | -1.62(-1.73–-1.51) | 414.14(322.7–527.31) | 16.45 | -1.9(-2.04–-1.75) |
| Poland | 0.08(0.07–0.1) | -50.16 | -5.34(-6.36–-4.31) | 5.41(4.54–6.27) | -46.21 | -4.72(-5.68–-3.76) |
| Portugal | 0.4(0.37–0.43) | -57.55 | -4.54(-5.17–-3.9) | 21.97(20.15–23.89) | -61.57 | -4.66(-5.29–-4.04) |
| Puerto Rico | 19.72(15.86–24.36) | -1.74 | 0.11(-0.41–0.63) | 1160.19(935.93–1425.06) | -5.53 | 0.1(-0.42–0.62) |
| Qatar | 0.13(0.07–0.2) | 396.13 | -1.21(-1.49–-0.93) | 8.4(5.34–12.3) | 448.94 | -1.03(-1.22–-0.85) |
| Republic of Korea | 0.02(0.02–0.03) | -37.53 | -3.71(-4.2–-3.23) | 2.47(1.93–3.01) | -0.3 | -1.61(-1.92–-1.3) |
| Republic of Moldova | 0.29(0.25–0.33) | -75.26 | -7.07(-8.06–-6.07) | 16.38(14.19–18.65) | -76.53 | -7.13(-8.12–-6.13) |
| Romania | 0.06(0.05–0.08) | -68.52 | -3.95(-4.57–-3.32) | 4.67(3.94–5.54) | -62.94 | -3.08(-3.62–-2.54) |
| Russian Federation | 0.72(0.6–0.86) | -41.88 | -4.27(-5.35–-3.18) | 38.81(32.69–45.78) | -47.23 | -4.47(-5.55–-3.38) |
| Rwanda | 1.06(0.7–1.52) | -1.31 | -2.42(-2.82–-2.02) | 60.57(39.46–87.07) | -4.9 | -2.38(-2.79–-1.98) |
| Saint Kitts and Nevis | 10.99(5.95–15.66) | 104.45 | 3.32(2.15–4.51) | 607.69(328.18–857.03) | 93.1 | 3.66(2.43–4.91) |
| Saint Lucia | 9.14(7.67–10.7) | 161.3 | 2.75(2.17–3.33) | 518.92(436.68–607.06) | 145.83 | 2.83(2.23–3.43) |
| Saint Vincent and the Grenadines | 9.11(7.66–10.6) | 45.75 | 1.93(1.23–2.64) | 512.12(432.54–594.17) | 33.71 | 1.85(1.16–2.55) |
| Samoa | 0.95(0.54–1.52) | -6.94 | -1.58(-1.74–-1.43) | 49.11(27.23–78.86) | -11.93 | -1.63(-1.79–-1.46) |
| San Marino | 0.08(0.05–0.12) | 24.36 | -0.27(-0.51–-0.03) | 5.31(3.74–7.66) | 29.3 | -0.07(-0.28–0.14) |
| Sao Tome and Principe | 1.54(1.01–2.28) | 109.21 | -0.14(-0.49–0.21) | 87.79(56.96–133.21) | 97.76 | -0.15(-0.5–0.21) |
| Saudi Arabia | 0.17(0.11–0.34) | 365.14 | 2.84(2.08–3.6) | 10.37(6.94–18.1) | 336.67 | 2.69(2.01–3.36) |
| Senegal | 0.44(0.27–0.63) | 79.31 | -0.01(-0.79–0.78) | 24.95(14.9–36.39) | 72.09 | 0.02(-0.77–0.82) |
| Serbia | 0.82(0.63–1.06) | -61.64 | -4.79(-5.27–-4.31) | 42.9(33.36–55.29) | -65.52 | -5.03(-5.53–-4.53) |
| Seychelles | 1.69(1.11–2.74) | 16.13 | -1.58(-1.75–-1.4) | 86.72(56.7–143.94) | 6.74 | -1.59(-1.77–-1.42) |
| Sierra Leone | 0.5(0.33–0.75) | 170.31 | -0.38(-1.24–0.49) | 29.98(19.14–44.56) | 168.16 | -0.28(-1.12–0.56) |
| Singapore | 0.01(0.01–0.01) | -32.68 | -4.22(-5.11–-3.33) | 2.24(1.76–2.8) | 44.5 | -1.25(-1.75–-0.74) |
| Slovakia | 0.25(0.18–0.34) | -68.54 | -5.17(-5.61–-4.72) | 14.51(10.77–19.22) | -69.04 | -5.04(-5.49–-4.58) |
| Slovenia | 0.18(0.14–0.23) | -46.7 | -4.78(-6.09–-3.45) | 10.73(8.71–13.32) | -47.1 | -4.33(-5.52–-3.13) |
| Solomon Islands | 1.91(1.34–2.65) | 24.73 | -2.53(-3.05–-2.01) | 101.97(70.42–142.17) | 25.61 | -2.45(-2.97–-1.93) |
| Somalia | 5.06(2.65–9.04) | 429.45 | 1.66(1.17–2.14) | 249.39(130.13–448.31) | 428.82 | 1.49(1.01–1.97) |
| South Africa | 4.87(3.99–6.33) | -47.64 | -5.27(-5.93–-4.61) | 263.2(211.47–351.75) | -48.59 | -5.25(-5.9–-4.6) |
| South Sudan | 2.78(1.51–4.71) | 118.93 | 1.08(0.79–1.37) | 136.94(73.49–231.52) | 109.26 | 0.94(0.68–1.2) |
| Spain | 0.13(0.12–0.15) | -29.85 | -2.68(-3.39–-1.98) | 8.34(7.6–9.16) | -28.48 | -2.35(-2.96–-1.72) |
| Sri Lanka | 1(0.69–1.42) | -42 | -3.54(-4.17–-2.91) | 56.72(39.17–80.35) | -46.17 | -3.73(-4.37–-3.08) |
| Sudan | 0.51(0.27–0.79) | 382.79 | -0.08(-3.57–3.55) | 29.26(15.89–44.93) | 388.4 | 0.01(-3.48–3.62) |
| Suriname | 2.89(2.16–3.78) | 67.56 | 0.75(0.5–1.01) | 165.66(123.31–217.08) | 55.77 | 0.6(0.35–0.84) |
| Sweden | 0.25(0.23–0.28) | 16.83 | 0.32(-0.38–1.02) | 15.79(14.45–17.4) | 20.44 | 0.51(-0.17–1.2) |
| Switzerland | 0.18(0.17–0.2) | -63.42 | -5.81(-6.47–-5.14) | 11.05(10.12–12.22) | -62.46 | -5.54(-6.16–-4.9) |
| Syrian Arab Republic | 1(0.7–1.42) | 57.09 | 1.1(0.58–1.62) | 53.02(37.64–75.49) | 40.01 | 0.86(0.4–1.31) |
| Taiwan  (Province of China) | 0.11(0.09–0.14) | -66.84 | -5.44(-6.38–-4.49) | 7.56(6.09–9.29) | -64.16 | -4.91(-5.77–-4.04) |
| Tajikistan | 0.18(0.14–0.27) | -22.69 | -7.95(-9.55–-6.31) | 9.85(7.15–14.3) | -19.89 | -8.01(-9.65–-6.34) |
| Thailand | 3.11(2.12–4.36) | -61.52 | -5.2(-5.68–-4.71) | 173.42(119.52–241.07) | -64.34 | -5.11(-5.59–-4.62) |
| Timor–Leste | 0.89(0.37–1.5) | 48.93 | -0.65(-1.01–-0.29) | 48.57(19.59–81.12) | 46.78 | -0.73(-1.12–-0.33) |
| Togo | 2.51(1.67–3.56) | 212.92 | 0.64(0.4–0.88) | 128.44(84.23–184) | 191.02 | 0.59(0.36–0.82) |
| Tokelau | 0.45(0.19–0.83) | -38.4 | -1.72(-1.82–-1.61) | 23.32(9.92–43.82) | -40.85 | -1.71(-1.82–-1.6) |
| Tonga | 0.23(0.13–0.34) | -82.71 | -7.25(-8.6–-5.87) | 11.83(6.95–18.44) | -82.66 | -7.13(-8.48–-5.75) |
| Trinidad and Tobago | 15.21(11.56–19.66) | 272.61 | 5.85(4.43–7.3) | 877.92(671.55–1128.98) | 255.88 | 5.93(4.49–7.39) |
| Tunisia | 0.2(0.13–0.34) | 22.85 | -0.62(-0.84–-0.4) | 12.31(8.11–20.33) | 22.68 | -0.38(-0.58–-0.17) |
| Turkey | 1.05(0.82–1.4) | -34.57 | -3.9(-4.3–-3.51) | 56.79(44.75–74.87) | -38.65 | -3.84(-4.24–-3.44) |
| Turkmenistan | 0.62(0.36–0.84) | -38.37 | -3.99(-4.77–-3.2) | 34.31(20.25–46.62) | -39.17 | -3.91(-4.7–-3.11) |
| Tuvalu | 1.11(0.67–1.7) | 3.53 | -0.89(-0.98–-0.8) | 58.29(34.22–90.02) | 2.31 | -0.91(-1.01–-0.81) |
| Uganda | 2.2(1.51–3.09) | 201.12 | 0.29(0.07–0.51) | 122.07(82.72–173.45) | 200.76 | 0.37(0.12–0.61) |
| Ukraine | 0.62(0.48–0.77) | 24.72 | -1(-1.92–-0.07) | 34.48(26.61–42.16) | 17 | -1.08(-2.01–-0.14) |
| United Arab Emirates | 0.3(0.12–0.58) | 257.75 | -0.98(-1.34–-0.62) | 17.54(8–32.85) | 247.51 | -0.77(-1.11–-0.42) |
| United Kingdom | 0.04(0.04–0.04) | -66.49 | -5.09(-5.66–-4.51) | 3.7(3.2–4.29) | -48.43 | -3.58(-4.05–-3.1) |
| United Republic of Tanzania | 2.22(1.59–3.09) | 134.22 | 0.15(0.05–0.25) | 123.96(87.4–173.36) | 130.34 | 0.27(0.16–0.37) |
| United States of America | 4.12(3.83–4.25) | -22.33 | -1.6(-1.97–-1.23) | 251.94(234.1–260.82) | -23.69 | -1.61(-1.98–-1.25) |
| United States Virgin Islands | 19.29(14.54–26.23) | -22.62 | -1.03(-1.55–-0.51) | 1021.48(767.21–1389.62) | -34.67 | -1.48(-2.12–-0.85) |
| Uruguay | 4.02(3.66–4.4) | 38.7 | 1.49(0.74–2.24) | 228.58(207.23–250.92) | 46.12 | 1.69(0.95–2.43) |
| Uzbekistan | 0.08(0.07–0.1) | -59.14 | -6.15(-7.15–-5.13) | 5.12(4.23–6.14) | -54.85 | -5.86(-6.86–-4.84) |
| Vanuatu | 0.17(0.1–0.28) | 65.96 | -1.35(-1.53–-1.16) | 9.57(5.67–15.35) | 66.02 | -1.28(-1.46–-1.1) |
| Venezuela | 32.75(25.2–42.27) | 305.4 | 3.91(3.13–4.7) | 1909.09(1476.7–2440.01) | 282.62 | 3.93(3.18–4.68) |
| Viet Nam | 0.12(0.04–0.36) | -19.69 | -3.43(-3.74–-3.12) | 7.2(3.35–20.11) | -15.87 | -3.04(-3.31–-2.76) |
| Yemen | 0.83(0.48–1.35) | 295.1 | 3.74(2.56–4.93) | 46.02(26.43–74.82) | 307.66 | 3.78(2.66–4.91) |
| Zambia | 3.71(2.51–4.98) | 280.34 | 2.43(1.99–2.87) | 180.16(122.19–246.76) | 230.23 | 1.64(1.23–2.04) |
| Zimbabwe | 0.88(0.55–1.32) | 92.68 | 0.42(0.22–0.61) | 39.96(27.26–53.34) | 93.33 | 0.61(0.38–0.85) |

DALYs: disability adjusted life years; EAPC: estimated annual percentage change; ASR, age-standardized rate; CI, confidence interval; UI: uncertainty interval.

**Table S7**. Incident burden and trends of self-harm by firearm at the national level from 1990 to 2019.

|  | **1990** | | **2019** | | **1990-2019** | |
| --- | --- | --- | --- | --- | --- | --- |
| **Characteristics** | Number  ×10^2^ (95% UI) | ASR/100,000  (95% UI) | Number  ×10^2^ (95% UI) | ASR/100,000  (95% UI) | Percent (%) | EAPC  (95%CI) |
| Afghanistan | 1.49(1.04–2.07) | 1.86(1.29–2.59) | 4.35(2.99–6.12) | 2.09(1.5–2.87) | 191.65 | 0.41(0.35–0.46) |
| Albania | 0.46(0.35–0.6) | 1.71(1.3–2.2) | 1.05(0.8–1.34) | 3.02(2.29–3.89) | 124.86 | 1.89(1.76–2.02) |
| Algeria | 2.51(1.71–3.47) | 1.49(1.03–2.04) | 6.68(4.59–9.32) | 1.57(1.1–2.16) | 166.28 | -0.01(-0.14–0.13) |
| American Samoa | 0.01(0.01–0.01) | 2.43(1.75–3.31) | 0.01(0.01–0.02) | 2.31(1.65–3.2) | 21.87 | -0.4(-0.56–-0.23) |
| Andorra | 0.02(0.02–0.03) | 3.46(2.49–4.63) | 0.04(0.03–0.05) | 3.08(2.23–4.04) | 78.71 | -0.63(-0.76–-0.5) |
| Angola | 1.14(0.82–1.57) | 2.19(1.56–2.99) | 3.96(2.85–5.44) | 2.6(1.88–3.52) | 246.32 | 0.58(0.51–0.66) |
| Antigua and Barbuda | 0.01(0–0.01) | 1.19(0.86–1.6) | 0.02(0.01–0.02) | 1.52(1.09–2.02) | 146.39 | 1.08(0.94–1.23) |
| Argentina | 13.24(10.78–16.32) | 4.12(3.35–5.09) | 20.44(16.11–25.38) | 4.17(3.3–5.19) | 54.46 | -0.53(-0.89–-0.18) |
| Armenia | 0.34(0.25–0.46) | 1.11(0.81–1.5) | 1.03(0.76–1.38) | 2.57(1.9–3.41) | 202.29 | 3.84(3.55–4.14) |
| Australia | 4.37(3.34–5.61) | 2.37(1.82–3.04) | 4.89(3.51–6.57) | 1.56(1.1–2.12) | 12.06 | -1.92(-2.22–-1.62) |
| Austria | 6.1(4.81–7.64) | 6.42(5.06–8.14) | 6.19(4.9–7.71) | 4.6(3.63–5.76) | 1.5 | -1.48(-1.6–-1.37) |
| Azerbaijan | 0.59(0.42–0.79) | 1.02(0.73–1.36) | 1.55(1.11–2.14) | 1.36(0.99–1.84) | 161.88 | 1.53(1.25–1.82) |
| Bahamas | 0.04(0.03–0.05) | 1.65(1.21–2.19) | 0.07(0.05–0.09) | 1.62(1.18–2.14) | 86.1 | -0.01(-0.14–0.12) |
| Bahrain | 0.07(0.05–0.1) | 1.8(1.3–2.42) | 0.37(0.25–0.51) | 2(1.45–2.69) | 424.65 | 0.28(0.06–0.5) |
| Bangladesh | 14.99(9.92–21.36) | 1.96(1.36–2.69) | 28.76(19.48–39.46) | 1.83(1.26–2.5) | 91.87 | -0.47(-0.63–-0.31) |
| Barbados | 0.04(0.03–0.06) | 1.73(1.28–2.27) | 0.07(0.05–0.09) | 1.81(1.31–2.41) | 55.73 | 0.1(0–0.2) |
| Belarus | 10.13(7.32–13.71) | 8.18(5.98–11.04) | 11.47(8.5–15.25) | 8.22(6.1–11.01) | 13.23 | 0.13(-0.35–0.61) |
| Belgium | 5.94(4.62–7.55) | 4.89(3.77–6.32) | 5.94(4.46–7.72) | 3.73(2.78–4.84) | 0 | -1.38(-1.52–-1.23) |
| Belize | 0.02(0.02–0.03) | 1.92(1.43–2.45) | 0.1(0.07–0.13) | 2.55(1.92–3.33) | 294.72 | 0.7(0.48–0.93) |
| Benin | 0.35(0.25–0.49) | 1.5(1.06–2.06) | 1.14(0.81–1.54) | 1.78(1.28–2.44) | 221.56 | 0.6(0.49–0.7) |
| Bermuda | 0.01(0.01–0.01) | 1.61(1.16–2.17) | 0.01(0.01–0.02) | 1.58(1.13–2.11) | 27.66 | -0.16(-0.27–-0.05) |
| Bhutan | 0.1(0.07–0.15) | 2.37(1.62–3.3) | 0.21(0.14–0.29) | 2.82(1.95–3.86) | 103.78 | 0.54(0.41–0.68) |
| Bolivia | 0.57(0.42–0.76) | 1.26(0.93–1.67) | 1.7(1.23–2.28) | 1.6(1.16–2.13) | 196.2 | 0.66(0.59–0.72) |
| Bosnia and Herzegovina | 1.37(1.02–1.81) | 2.89(2.17–3.74) | 2.07(1.56–2.64) | 4.01(3.04–5.18) | 50.51 | 1.68(1.45–1.92) |
| Botswana | 0.23(0.16–0.33) | 3.02(2.11–4.22) | 0.79(0.54–1.12) | 3.85(2.69–5.3) | 237.96 | 0.5(0.14–0.86) |
| Brazil | 20.06(13.45–28.4) | 1.68(1.15–2.38) | 49.32(33.76–70.53) | 2.03(1.39–2.91) | 145.81 | 0.81(0.6–1.02) |
| Brunei Darussalam | 0.02(0.01–0.03) | 1.01(0.69–1.43) | 0.05(0.03–0.07) | 1.08(0.73–1.51) | 152.94 | 0.4(0.14–0.66) |
| Bulgaria | 6.11(4.51–7.9) | 5.2(3.85–6.75) | 5.21(4.01–6.74) | 4.66(3.56–6.01) | -14.82 | -0.57(-0.65–-0.48) |
| Burkina Faso | 0.86(0.61–1.17) | 1.72(1.22–2.31) | 2.34(1.69–3.18) | 2(1.46–2.73) | 171.42 | 0.51(0.45–0.57) |
| Burundi | 0.75(0.53–1.03) | 2.72(1.92–3.77) | 1.54(1.12–2.09) | 2.53(1.84–3.39) | 105.26 | -0.2(-0.32–-0.08) |
| Cabo Verde | 0.05(0.04–0.07) | 2.31(1.68–3.15) | 0.17(0.12–0.24) | 3.38(2.42–4.59) | 248.8 | 1.43(1.3–1.55) |
| Cambodia | 0.43(0.27–0.64) | 0.67(0.43–0.99) | 1.57(1.01–2.28) | 1.05(0.69–1.5) | 266.83 | 1.5(1.44–1.56) |
| Cameroon | 0.99(0.7–1.34) | 1.78(1.28–2.4) | 3.82(2.71–5.22) | 2.39(1.72–3.21) | 287.5 | 1.15(0.98–1.32) |
| Canada | 8.09(5.92–10.77) | 2.68(1.97–3.54) | 8.98(6.4–12.26) | 1.93(1.38–2.65) | 10.95 | -1.47(-1.68–-1.27) |
| Central African Republic | 0.53(0.37–0.74) | 3.49(2.49–4.84) | 1.01(0.7–1.4) | 3.28(2.3–4.48) | 90.23 | -0.09(-0.17–-0.02) |
| Chad | 0.44(0.32–0.61) | 1.39(0.99–1.9) | 1.28(0.9–1.74) | 1.76(1.26–2.38) | 190.18 | 0.94(0.78–1.09) |
| Chile | 6.31(4.89–7.98) | 5.25(4.1–6.64) | 7.25(5.45–9.61) | 3.33(2.48–4.37) | 14.88 | -2.29(-2.56–-2.03) |
| China | 652.22(287.07–1127.94) | 6.09(2.76–10.23) | 1007.89(461.02–1692.36) | 5.24(2.36–8.7) | 54.53 | -0.46(-0.82–-0.09) |
| Colombia | 5.3(3.98–6.87) | 1.78(1.38–2.25) | 9.69(7.32–12.63) | 1.88(1.42–2.45) | 82.76 | 0.09(-0.14–0.32) |
| Comoros | 0.03(0.02–0.04) | 1(0.68–1.42) | 0.07(0.05–0.09) | 1.17(0.82–1.66) | 160.39 | 0.52(0.39–0.65) |
| Congo | 0.43(0.3–0.58) | 3.29(2.33–4.48) | 0.99(0.71–1.38) | 2.89(2.05–3.94) | 131.91 | -0.47(-0.6–-0.33) |
| Cook Islands | 0.01(0.01–0.01) | 4.76(3.37–6.53) | 0.01(0.01–0.01) | 4.73(3.32–6.54) | 17.19 | -0.19(-0.42–0.03) |
| Costa Rica | 0.52(0.4–0.67) | 2.18(1.73–2.73) | 1.19(0.92–1.5) | 2.27(1.75–2.86) | 127.04 | 0.2(0.12–0.28) |
| Croatia | 8.07(6.51–9.93) | 13.16(10.68–16.1) | 6.73(5.47–8.18) | 9.75(7.89–11.93) | -16.54 | -1.31(-1.45–-1.16) |
| Cuba | 4.58(3.3–6) | 4.21(3.04–5.54) | 5.31(3.81–7.03) | 3.31(2.39–4.38) | 16.04 | -1.05(-1.26–-0.84) |
| Cyprus | 0.13(0.1–0.18) | 1.66(1.21–2.22) | 0.3(0.22–0.4) | 1.78(1.29–2.39) | 123.45 | 0.23(0.13–0.33) |
| Czechia | 6.68(5.12–8.54) | 5.25(3.94–6.75) | 8.52(6.61–10.87) | 5.17(3.97–6.69) | 27.52 | -0.49(-0.66–-0.32) |
| Côte d'Ivoire | 1.25(0.89–1.68) | 2.18(1.56–2.91) | 3.23(2.3–4.38) | 2.17(1.56–2.94) | 158.73 | -0.07(-0.16–0.02) |
| DPR Korea | 13.68(9.38–19.29) | 6.93(4.84–9.61) | 16.13(11.12–22.66) | 4.94(3.45–6.94) | 17.9 | -1.39(-1.6–-1.17) |
| DR Congo | 4.16(2.98–5.71) | 2.09(1.49–2.83) | 11.13(8.04–15.13) | 2.35(1.7–3.21) | 167.21 | 0.41(0.33–0.5) |
| Denmark | 4.36(3.29–5.63) | 6.82(5.17–8.86) | 2.85(2.15–3.73) | 3.64(2.72–4.81) | -34.54 | -2.43(-2.69–-2.16) |
| Djibouti | 0.02(0.02–0.03) | 1.07(0.74–1.47) | 0.12(0.08–0.16) | 1.4(0.96–1.96) | 410.33 | 1.24(1.11–1.37) |
| Dominica | 0.01(0.01–0.01) | 1.62(1.18–2.15) | 0.01(0.01–0.02) | 1.73(1.24–2.28) | 30.65 | 0.24(0.14–0.35) |
| Dominican Republic | 0.85(0.62–1.12) | 1.55(1.16–2.01) | 2.97(2.24–3.88) | 2.78(2.1–3.6) | 248.86 | 2.04(1.82–2.25) |
| Ecuador | 1.17(0.84–1.56) | 1.43(1.06–1.87) | 3.83(2.74–5.12) | 2.23(1.61–2.93) | 227.33 | 1.63(1.36–1.9) |
| Egypt | 5.41(3.77–7.41) | 1.34(0.95–1.82) | 16.47(11.65–22.57) | 1.94(1.39–2.66) | 204.52 | 1.27(1.15–1.39) |
| El Salvador | 0.57(0.39–0.8) | 1.4(0.99–1.89) | 1.15(0.78–1.54) | 1.87(1.29–2.51) | 100.78 | 0.93(0.83–1.02) |
| Equatorial Guinea | 0.06(0.04–0.08) | 2.34(1.7–3.22) | 0.22(0.16–0.3) | 3.23(2.32–4.41) | 283.58 | 1.36(1.21–1.5) |
| Eritrea | 0.21(0.14–0.29) | 1.45(1–2) | 0.75(0.53–1.04) | 1.95(1.37–2.69) | 257.48 | 0.97(0.84–1.1) |
| Estonia | 2.49(1.88–3.22) | 13.34(10.03–17.28) | 1.38(1.05–1.8) | 7.11(5.38–9.24) | -44.46 | -2.59(-2.9–-2.28) |
| Eswatini | 0.12(0.08–0.16) | 2.77(1.95–3.85) | 0.36(0.25–0.51) | 4.28(3.01–5.96) | 209.84 | 1.65(1.42–1.89) |
| Ethiopia | 7.55(5.06–10.87) | 2.98(1.98–4.3) | 11.47(7.82–16.29) | 2.19(1.48–3.18) | 51.82 | -1.37(-1.49–-1.25) |
| Fiji | 0.21(0.14–0.3) | 3.35(2.3–4.59) | 0.34(0.23–0.47) | 3.69(2.53–5.06) | 61.1 | 0.22(0.05–0.38) |
| Finland | 6.46(5.12–8.1) | 10.93(8.63–13.66) | 3.28(2.57–4.13) | 4.54(3.47–5.84) | -49.3 | -3.08(-3.39–-2.77) |
| France | 57.38(45.48–71.27) | 8.56(6.76–10.69) | 48.28(38.41–60.2) | 5.23(4.05–6.71) | -15.85 | -1.96(-2.06–-1.87) |
| Gabon | 0.21(0.15–0.29) | 3.42(2.46–4.64) | 0.43(0.31–0.59) | 3.36(2.45–4.62) | 100.26 | -0.06(-0.14–0.02) |
| Gambia | 0.06(0.04–0.09) | 1.35(0.96–1.85) | 0.19(0.14–0.27) | 1.61(1.14–2.23) | 211.93 | 0.64(0.57–0.72) |
| Georgia | 1.04(0.76–1.4) | 1.66(1.22–2.21) | 1.32(1.01–1.69) | 2.62(2–3.33) | 27.44 | 1.77(1.52–2.02) |
| Germany | 38.46(29.27–49.45) | 3.67(2.76–4.77) | 42.44(32.03–54.85) | 3.28(2.4–4.31) | 10.35 | -0.66(-0.79–-0.53) |
| Ghana | 1.11(0.79–1.53) | 1.35(0.97–1.85) | 3.72(2.68–5.14) | 1.78(1.28–2.4) | 235.06 | 0.98(0.9–1.06) |
| Greece | 2.15(1.64–2.79) | 1.74(1.3–2.29) | 2.95(2.27–3.78) | 2.02(1.53–2.63) | 37.24 | 0.63(0.54–0.73) |
| Greenland | 0.07(0.05–0.1) | 11.34(8.11–15.74) | 0.05(0.04–0.07) | 7.87(5.63–10.84) | -22.32 | -1.55(-1.67–-1.43) |
| Grenada | 0.01(0.01–0.02) | 1.81(1.32–2.41) | 0.02(0.02–0.03) | 1.91(1.39–2.53) | 85.59 | 0.09(-0.01–0.19) |
| Guam | 0.09(0.06–0.12) | 6.42(4.71–8.66) | 0.12(0.08–0.16) | 6.91(4.95–9.61) | 30.51 | -0.03(-0.15–0.1) |
| Guatemala | 0.85(0.62–1.12) | 1.47(1.1–1.89) | 1.8(1.26–2.43) | 1.13(0.82–1.48) | 111.11 | -1.22(-2.07–-0.35) |
| Guinea | 0.46(0.32–0.63) | 1.23(0.87–1.68) | 1.12(0.8–1.52) | 1.68(1.2–2.27) | 146.84 | 1.21(1.1–1.31) |
| Guinea–Bissau | 0.11(0.08–0.15) | 2.12(1.5–2.88) | 0.24(0.17–0.34) | 2.41(1.72–3.27) | 123.36 | 0.51(0.45–0.58) |
| Guyana | 0.22(0.15–0.31) | 4.01(2.79–5.49) | 0.39(0.26–0.54) | 5.11(3.5–7.03) | 74.1 | 0.77(0.69–0.85) |
| Haiti | 0.89(0.64–1.19) | 1.94(1.42–2.59) | 2.02(1.4–2.77) | 1.94(1.4–2.62) | 127.09 | -0.02(-0.2–0.15) |
| Honduras | 0.34(0.24–0.45) | 1.03(0.76–1.35) | 0.77(0.55–1.02) | 0.96(0.7–1.27) | 128.52 | -0.44(-0.63–-0.24) |
| Hungary | 11.1(8.08–14.48) | 8.24(6.02–10.8) | 10.57(7.9–13.62) | 6.89(5.12–8.95) | -4.74 | -0.71(-0.8–-0.61) |
| Iceland | 0.11(0.08–0.14) | 4.13(3.21–5.22) | 0.13(0.1–0.17) | 3.01(2.28–3.89) | 22.08 | -1.35(-1.43–-1.27) |
| India | 401.91(254.8–597.71) | 5.62(3.69–8.29) | 817.03(525.33–1203.57) | 5.83(3.8–8.54) | 103.29 | -0.07(-0.19–0.05) |
| Indonesia | 4.86(2.84–7.81) | 0.34(0.2–0.54) | 7.34(4.39–11.64) | 0.27(0.16–0.42) | 50.88 | -1.35(-1.66–-1.04) |
| Iran | 9.02(6.03–12.72) | 2.33(1.58–3.27) | 22.62(15.05–32.12) | 2.44(1.67–3.42) | 150.95 | -0.11(-0.29–0.07) |
| Iraq | 3.54(2.64–4.65) | 3.16(2.38–4.12) | 12.52(9.39–16.49) | 3.6(2.75–4.66) | 253.91 | 0.46(0.4–0.52) |
| Ireland | 0.98(0.73–1.3) | 2.73(2.04–3.62) | 1.7(1.23–2.3) | 2.82(1.99–3.83) | 73.32 | -0.07(-0.22–0.08) |
| Israel | 1.33(1.03–1.66) | 2.82(2.2–3.55) | 2.26(1.73–2.91) | 2.33(1.78–3.01) | 70.6 | -1.05(-1.45–-0.66) |
| Italy | 30.01(20.95–42.35) | 4.08(2.83–5.79) | 35.74(25.54–49.33) | 3.95(2.76–5.46) | 19.11 | 0.07(-0.35–0.5) |
| Jamaica | 0.22(0.16–0.3) | 1.16(0.83–1.56) | 0.47(0.36–0.61) | 1.55(1.18–2.02) | 111.59 | 1.36(1.19–1.54) |
| Japan | 34.24(21.3–53.2) | 2.1(1.31–3.25) | 51.12(33.49–77.2) | 2.76(1.74–4.19) | 49.28 | 1.2(1.06–1.33) |
| Jordan | 0.32(0.22–0.44) | 1.5(1.06–2.04) | 1.65(1.17–2.24) | 1.65(1.2–2.23) | 411.43 | 0.23(0.08–0.38) |
| Kazakhstan | 7.09(5.08–9.7) | 4.96(3.58–6.78) | 9.79(6.79–13.59) | 4.97(3.45–6.81) | 38.09 | 0.23(-0.18–0.64) |
| Kenya | 2.31(1.56–3.3) | 2.26(1.52–3.28) | 7.46(5.06–10.63) | 2.62(1.79–3.76) | 223.09 | 0.88(0.68–1.08) |
| Kiribati | 0.03(0.02–0.05) | 5.55(3.56–8.06) | 0.06(0.03–0.09) | 5.28(3.34–7.86) | 70.76 | -0.36(-0.46–-0.25) |
| Kuwait | 0.21(0.14–0.29) | 1.47(1.07–1.96) | 0.96(0.65–1.35) | 1.85(1.33–2.48) | 355.05 | 0.71(0.6–0.82) |
| Kyrgyzstan | 0.94(0.66–1.3) | 2.9(2.02–4.03) | 1.18(0.8–1.67) | 2.09(1.45–2.94) | 25.23 | -1.49(-1.67–-1.31) |
| Lao People's Democratic Republic | 0.26(0.17–0.39) | 0.92(0.59–1.35) | 0.52(0.32–0.77) | 0.84(0.53–1.25) | 99.76 | -0.69(-0.83–-0.55) |
| Latvia | 4.48(3.37–5.74) | 13.73(10.43–17.68) | 2.46(1.86–3.15) | 8.64(6.54–11.16) | -45.09 | -1.79(-2–-1.59) |
| Lebanon | 0.42(0.3–0.58) | 1.59(1.13–2.17) | 1.12(0.79–1.52) | 2.09(1.48–2.83) | 167.16 | 1.03(0.81–1.25) |
| Lesotho | 0.33(0.22–0.45) | 2.79(1.95–3.91) | 0.96(0.67–1.37) | 5.59(3.89–7.83) | 194.59 | 2.83(2.65–3.01) |
| Liberia | 0.18(0.13–0.25) | 1.47(1.04–2.02) | 0.46(0.32–0.65) | 1.63(1.14–2.29) | 154.39 | 0.49(0.38–0.6) |
| Libya | 0.41(0.28–0.57) | 1.52(1.05–2.08) | 1.46(1.01–2.04) | 1.97(1.41–2.66) | 256.63 | 1.04(0.93–1.16) |
| Lithuania | 4.75(3.42–6.38) | 11.16(8.03–14.98) | 3.82(2.72–5.17) | 9(6.38–12.15) | -19.56 | -0.98(-1.28–-0.69) |
| Luxembourg | 0.29(0.22–0.36) | 6.08(4.68–7.67) | 0.44(0.34–0.57) | 5.46(4.13–7.05) | 54.2 | -0.61(-0.74–-0.48) |
| Madagascar | 0.76(0.53–1.05) | 1.2(0.83–1.67) | 1.98(1.35–2.76) | 1.29(0.9–1.8) | 159.36 | 0.26(0.14–0.37) |
| Malawi | 0.74(0.52–1.04) | 1.5(1.06–2.07) | 1.6(1.15–2.19) | 1.64(1.18–2.2) | 114.56 | 0.41(0.26–0.56) |
| Malaysia | 0.99(0.62–1.45) | 0.76(0.48–1.12) | 3.09(1.99–4.59) | 0.97(0.63–1.43) | 211.61 | 0.82(0.75–0.9) |
| Maldives | 0.01(0.01–0.01) | 0.63(0.4–0.93) | 0.04(0.02–0.06) | 0.74(0.47–1.08) | 404.65 | 0.25(0–0.5) |
| Mali | 0.66(0.46–0.92) | 1.35(0.95–1.87) | 1.62(1.14–2.22) | 1.5(1.08–2.07) | 144.32 | 0.32(0.23–0.4) |
| Malta | 0.08(0.06–0.11) | 1.94(1.45–2.53) | 0.13(0.09–0.16) | 2.18(1.59–2.89) | 54.62 | 0.21(-0.03–0.44) |
| Marshall Islands | 0.03(0.02–0.04) | 7.81(5.44–10.75) | 0.04(0.03–0.06) | 7.28(5.06–10.16) | 61.82 | -0.36(-0.42–-0.3) |
| Mauritania | 0.15(0.11–0.21) | 1.35(0.95–1.88) | 0.33(0.23–0.47) | 1.37(0.96–1.91) | 115.24 | -0.09(-0.22–0.03) |
| Mauritius | 0.1(0.06–0.16) | 1.01(0.62–1.52) | 0.14(0.09–0.21) | 0.88(0.54–1.33) | 39.42 | -0.86(-0.99–-0.73) |
| Mexico | 7.5(4.92–10.78) | 1.15(0.78–1.64) | 18.35(11.87–26.64) | 1.41(0.92–2.04) | 144.76 | 0.62(0.06–1.18) |
| Micronesia | 0.06(0.04–0.08) | 7.56(5.34–10.6) | 0.07(0.05–0.1) | 7.22(4.95–10.05) | 21.2 | -0.29(-0.35–-0.23) |
| Monaco | 0.02(0.01–0.02) | 4.15(3.04–5.61) | 0.02(0.02–0.03) | 4.11(3.01–5.53) | 23.39 | -0.06(-0.09–-0.03) |
| Mongolia | 0.32(0.22–0.43) | 2.42(1.72–3.36) | 1.05(0.7–1.5) | 3.09(2.13–4.37) | 230.69 | 1.07(0.85–1.29) |
| Montenegro | 0.54(0.43–0.69) | 8.26(6.53–10.46) | 0.84(0.66–1.06) | 9.54(7.53–12.12) | 54.41 | 0.66(0.51–0.81) |
| Morocco | 3.37(2.3–4.67) | 1.86(1.3–2.57) | 9.03(6.3–12.4) | 2.44(1.72–3.33) | 168.18 | 0.87(0.77–0.96) |
| Mozambique | 0.81(0.56–1.1) | 1.08(0.76–1.49) | 2.96(2.11–4.03) | 2.01(1.44–2.71) | 266.83 | 2.55(2.42–2.68) |
| Myanmar | 2.26(1.41–3.36) | 0.72(0.46–1.06) | 3.68(2.35–5.43) | 0.66(0.43–0.98) | 62.65 | -0.56(-0.67–-0.45) |
| Namibia | 0.17(0.12–0.24) | 1.99(1.4–2.74) | 0.54(0.38–0.75) | 2.93(2.08–4.01) | 215.18 | 1.27(1.12–1.42) |
| Nauru | 0.01(0–0.01) | 7.21(5.03–10.01) | 0.01(0–0.01) | 6.84(4.79–9.57) | 9.13 | -0.43(-0.51–-0.34) |
| Nepal | 4.14(2.77–5.82) | 2.91(2–4.01) | 9.65(6.54–13.28) | 3.51(2.39–4.82) | 132.95 | 0.48(0.41–0.54) |
| Netherlands | 4.55(3.19–6.28) | 2.59(1.79–3.56) | 6.75(4.83–9.12) | 2.94(2.04–4.02) | 48.33 | 0.56(0.34–0.78) |
| New Zealand | 0.73(0.49–1.04) | 1.99(1.33–2.86) | 0.36(0.23–0.55) | 0.64(0.4–0.97) | -49.94 | -5.77(-6.37–-5.17) |
| Nicaragua | 0.3(0.22–0.39) | 1.24(0.93–1.61) | 0.78(0.57–1.03) | 1.33(0.99–1.74) | 162.79 | 0.44(0.33–0.55) |
| Niger | 0.44(0.31–0.61) | 1.2(0.85–1.65) | 1.42(1–1.94) | 1.42(1.01–1.94) | 220.09 | 0.67(0.61–0.73) |
| Nigeria | 7.57(5.11–10.93) | 1.44(0.98–2.08) | 17.37(11.8–25.05) | 1.51(1.03–2.16) | 129.53 | 0.25(0.16–0.35) |
| Niue | 0(0–0) | 4.94(3.48–6.85) | 0(0–0) | 4.29(2.98–5.99) | -22.68 | -0.74(-0.83–-0.64) |
| North Macedonia | 0.6(0.45–0.78) | 2.97(2.2–3.83) | 1.14(0.87–1.47) | 3.73(2.87–4.75) | 89.07 | 0.91(0.85–0.97) |
| Northern Mariana Islands | 0.03(0.02–0.04) | 5.51(4.07–7.44) | 0.03(0.02–0.03) | 5.2(3.68–7.07) | -5.94 | -0.39(-0.46–-0.32) |
| Norway | 3.15(2.23–4.34) | 6.57(4.62–9.16) | 2.58(1.79–3.63) | 3.83(2.62–5.51) | -18.2 | -2.09(-2.19–-2) |
| Oman | 0.2(0.13–0.28) | 1.54(1.09–2.08) | 0.74(0.48–1.05) | 1.71(1.22–2.32) | 268.38 | 0.22(0.06–0.38) |
| Pakistan | 26.51(16.77–39.46) | 3.26(2.13–4.74) | 74.29(46.34–111.81) | 4(2.61–5.92) | 180.28 | 0.75(0.6–0.9) |
| Palau | 0.01(0.01–0.01) | 5.05(3.56–6.91) | 0.01(0.01–0.02) | 5.7(3.97–7.92) | 64.54 | 0.26(0.17–0.36) |
| Palestine | 0.15(0.11–0.21) | 1.34(0.95–1.82) | 0.59(0.41–0.8) | 1.59(1.14–2.11) | 280.38 | 0.46(0.26–0.65) |
| Panama | 0.24(0.18–0.32) | 1.23(0.92–1.58) | 0.55(0.4–0.72) | 1.3(0.96–1.71) | 123.95 | -0.15(-0.33–0.04) |
| Papua New Guinea | 0.71(0.48–0.99) | 2.19(1.54–2.98) | 1.89(1.31–2.62) | 2.2(1.59–2.97) | 166.14 | -0.15(-0.27–-0.03) |
| Paraguay | 0.38(0.28–0.49) | 1.23(0.93–1.58) | 1.27(0.97–1.63) | 1.9(1.47–2.43) | 235.56 | 1.51(1.43–1.58) |
| Peru | 1.54(1.06–2.08) | 0.91(0.64–1.2) | 4.11(2.95–5.53) | 1.19(0.86–1.58) | 167.38 | 1.11(1–1.22) |
| Philippines | 5.43(3.27–8.45) | 1.19(0.75–1.81) | 10.37(6.43–15.75) | 1.04(0.66–1.57) | 90.97 | -0.79(-0.97–-0.62) |
| Poland | 21.65(14.77–31.2) | 5.09(3.42–7.4) | 30.52(20.59–44.41) | 5.42(3.62–7.94) | 40.93 | 0.3(0.2–0.4) |
| Portugal | 4.01(3.07–5.12) | 3.36(2.57–4.28) | 6.4(5.02–8.07) | 3.71(2.88–4.72) | 59.78 | 0.49(0.31–0.67) |
| Puerto Rico | 1.19(0.9–1.55) | 3.31(2.48–4.33) | 0.99(0.73–1.31) | 2.21(1.63–2.93) | -17.17 | -1.45(-1.51–-1.39) |
| Qatar | 0.06(0.04–0.1) | 1.74(1.24–2.38) | 0.64(0.42–0.93) | 2.11(1.51–2.85) | 903.34 | 0.79(0.64–0.93) |
| Republic of Korea | 6.91(4.41–10.25) | 1.69(1.09–2.48) | 23.61(15.38–34.96) | 2.9(1.91–4.19) | 241.71 | 2.12(1.98–2.26) |
| Republic of Moldova | 2.5(1.79–3.38) | 5.4(3.86–7.32) | 2.71(1.93–3.64) | 5.16(3.65–6.92) | 8.16 | -0.1(-0.23–0.02) |
| Romania | 5.81(3.83–8.2) | 2.07(1.38–2.94) | 8.55(5.84–12.16) | 2.84(1.92–4.03) | 47.23 | 1.24(1.18–1.29) |
| Russian Federation | 259.23(179.93–373.74) | 14.84(10.2–21.14) | 213.1(148.57–302.68) | 10.56(7.25–14.93) | -17.79 | -1.42(-1.82–-1.02) |
| Rwanda | 0.65(0.45–0.91) | 1.8(1.24–2.49) | 1.19(0.84–1.64) | 1.52(1.07–2.1) | 82.04 | -0.69(-0.84–-0.53) |
| Saint Kitts and Nevis | 0(0–0.01) | 1.34(0.97–1.78) | 0.01(0.01–0.01) | 1.38(0.97–1.87) | 113.42 | -0.13(-0.37–0.12) |
| Saint Lucia | 0.02(0.02–0.03) | 2.16(1.58–2.85) | 0.05(0.04–0.06) | 2.3(1.66–3.06) | 121.32 | -0.04(-0.18–0.1) |
| Saint Vincent and the Grenadines | 0.02(0.01–0.02) | 1.88(1.34–2.49) | 0.03(0.02–0.04) | 2.07(1.5–2.75) | 75.86 | 0.38(0.26–0.49) |
| Samoa | 0.05(0.04–0.08) | 4.4(3.11–6.03) | 0.07(0.05–0.1) | 4.07(2.81–5.63) | 38.3 | -0.58(-0.67–-0.49) |
| San Marino | 0.01(0.01–0.01) | 3.91(2.94–5.16) | 0.02(0.01–0.02) | 3.56(2.67–4.68) | 50.27 | -0.42(-0.53–-0.31) |
| Sao Tome and Principe | 0(0–0.01) | 0.56(0.39–0.79) | 0.01(0.01–0.02) | 0.79(0.56–1.09) | 192.99 | 1.16(1.02–1.29) |
| Saudi Arabia | 1.19(0.82–1.66) | 1.15(0.83–1.55) | 8.02(5.49–11.25) | 2.13(1.55–2.86) | 572.61 | 2.51(2.29–2.74) |
| Senegal | 0.61(0.43–0.82) | 1.59(1.15–2.16) | 1.62(1.17–2.21) | 1.82(1.3–2.46) | 167.88 | 0.53(0.46–0.61) |
| Serbia | 8.84(6.89–11.26) | 7.84(6.14–9.92) | 9.72(7.61–12.12) | 7.44(5.82–9.25) | 9.98 | -0.32(-0.43–-0.2) |
| Seychelles | 0.01(0–0.01) | 1.03(0.65–1.56) | 0.01(0.01–0.02) | 1.17(0.74–1.73) | 137.84 | 0.07(-0.08–0.22) |
| Sierra Leone | 0.29(0.21–0.4) | 1.34(0.95–1.82) | 0.8(0.56–1.09) | 1.72(1.22–2.35) | 171.14 | 1.02(0.91–1.13) |
| Singapore | 0.39(0.25–0.57) | 1.3(0.85–1.88) | 1.07(0.73–1.52) | 1.34(0.92–1.88) | 172.63 | 0.22(0.15–0.28) |
| Slovakia | 4.24(3.3–5.4) | 7.43(5.75–9.45) | 5.45(4.18–6.97) | 6.91(5.3–8.86) | 28.38 | -0.35(-0.4–-0.3) |
| Slovenia | 2.88(2.2–3.68) | 12.17(9.28–15.46) | 3.23(2.51–4.06) | 9.44(7.36–11.86) | 12.08 | -1.06(-1.24–-0.88) |
| Solomon Islands | 0.2(0.14–0.29) | 8.37(5.79–11.56) | 0.48(0.32–0.68) | 8.37(5.84–11.63) | 134.43 | -0.29(-0.41–-0.18) |
| Somalia | 0.45(0.3–0.64) | 1.24(0.84–1.73) | 1.26(0.86–1.78) | 1.32(0.91–1.85) | 180.84 | 0.42(0.35–0.5) |
| South Africa | 10.24(6.52–15.6) | 3.63(2.34–5.48) | 15.95(10.67–23.52) | 2.9(1.93–4.21) | 55.71 | -0.81(-0.94–-0.67) |
| South Sudan | 0.34(0.24–0.47) | 1.14(0.8–1.57) | 0.56(0.39–0.76) | 1.09(0.77–1.49) | 64.86 | -0.07(-0.15–0.01) |
| Spain | 10(7.42–13.05) | 2.24(1.65–2.97) | 16.36(12.09–21.53) | 2.47(1.81–3.28) | 63.59 | 0.33(0.26–0.4) |
| Sri Lanka | 2.59(1.56–4) | 1.73(1.07–2.66) | 3.29(1.92–5.15) | 1.31(0.77–2.06) | 26.94 | -1.73(-2.03–-1.43) |
| Sudan | 1.66(1.11–2.37) | 1.24(0.85–1.77) | 4.69(3.11–6.65) | 1.51(1.05–2.09) | 182.8 | 0.51(0.42–0.6) |
| Suriname | 0.15(0.11–0.2) | 4.63(3.37–6.06) | 0.34(0.25–0.45) | 5.44(4.04–7.23) | 125.39 | 0.65(0.6–0.71) |
| Sweden | 5.9(4.02–8.48) | 5.53(3.75–7.96) | 4.86(3.34–6.93) | 3.66(2.49–5.28) | -17.54 | -1.66(-1.81–-1.5) |
| Switzerland | 7.74(6.26–9.54) | 9.27(7.46–11.49) | 4.82(3.82–6.06) | 3.69(2.9–4.67) | -37.8 | -3.53(-3.74–-3.32) |
| Syrian Arab Republic | 0.85(0.59–1.16) | 1.12(0.79–1.51) | 2.14(1.52–2.92) | 1.47(1.06–1.98) | 150.67 | 1.01(0.89–1.13) |
| Taiwan  (Province of China) | 12.35(8.68–16.48) | 6.3(4.5–8.37) | 29.92(21.08–40.19) | 9.05(6.25–12.08) | 142.33 | 1.31(0.93–1.68) |
| Tajikistan | 0.36(0.24–0.51) | 1.12(0.76–1.59) | 0.77(0.52–1.09) | 1.09(0.75–1.51) | 114.72 | -0.04(-0.37–0.29) |
| Thailand | 17.23(11.31–25.65) | 3.04(2.09–4.41) | 20.61(13.66–29.03) | 2.33(1.52–3.34) | 19.65 | -1.54(-1.89–-1.19) |
| Timor–Leste | 0.02(0.01–0.04) | 0.45(0.28–0.68) | 0.06(0.04–0.08) | 0.59(0.37–0.87) | 137.6 | 0.83(0.57–1.08) |
| Togo | 0.28(0.2–0.39) | 1.69(1.19–2.29) | 1.01(0.71–1.37) | 2.06(1.46–2.77) | 256.22 | 0.85(0.74–0.95) |
| Tokelau | 0(0–0) | 2.86(2.02–4.01) | 0(0–0) | 3.01(2.08–4.26) | 6.92 | 0.05(-0.02–0.11) |
| Tonga | 0.01(0.01–0.02) | 1.87(1.34–2.53) | 0.02(0.01–0.03) | 2.26(1.58–3.08) | 51.41 | 0.52(0.41–0.62) |
| Trinidad and Tobago | 0.29(0.2–0.41) | 2.81(1.96–3.82) | 0.46(0.32–0.62) | 2.72(1.89–3.69) | 55.1 | 0.02(-0.09–0.12) |
| Tunisia | 0.92(0.64–1.25) | 1.44(1.01–1.94) | 2.52(1.78–3.42) | 1.91(1.35–2.57) | 172.65 | 0.95(0.85–1.05) |
| Turkey | 13.16(9.93–17.02) | 2.67(2.05–3.45) | 21.13(16.35–27.35) | 2.26(1.74–2.9) | 60.5 | -0.9(-1.05–-0.75) |
| Turkmenistan | 0.32(0.22–0.45) | 1.41(0.97–1.99) | 0.78(0.53–1.1) | 1.61(1.12–2.24) | 140.97 | 0.37(0.22–0.51) |
| Tuvalu | 0(0–0) | 4.05(2.87–5.6) | 0.01(0–0.01) | 4.55(3.17–6.43) | 52.47 | 0.12(0.05–0.2) |
| Uganda | 1.11(0.79–1.54) | 1.36(0.96–1.88) | 2.96(2.08–4.13) | 1.53(1.06–2.09) | 165.95 | 0.38(0.15–0.61) |
| Ukraine | 86.84(60.59–124.4) | 13.1(9.08–18.88) | 74.7(52.27–105.63) | 12.12(8.34–17.12) | -13.98 | -0.92(-1.25–-0.58) |
| United Arab Emirates | 0.35(0.23–0.5) | 2.54(1.85–3.39) | 3.17(2.12–4.58) | 2.52(1.81–3.35) | 804.4 | -0.36(-0.51–-0.21) |
| United Kingdom | 17.3(11.83–24.58) | 2.54(1.71–3.66) | 22.19(15.24–31.71) | 2.63(1.76–3.78) | 28.26 | 0.07(-0.18–0.32) |
| United Republic of Tanzania | 1.93(1.36–2.69) | 1.42(1–1.97) | 4.76(3.37–6.55) | 1.5(1.07–2.04) | 147.28 | 0.25(0.15–0.36) |
| United States of America | 116.64(78.92–165.97) | 4.18(2.84–5.93) | 164.06(111.57–231.32) | 4.19(2.86–5.99) | 40.66 | -0.53(-0.87–-0.18) |
| United States Virgin Islands | 0.02(0.02–0.03) | 2.38(1.78–3.09) | 0.04(0.03–0.05) | 2.49(1.85–3.26) | 41.27 | 0.12(0.05–0.18) |
| Uruguay | 2.1(1.72–2.55) | 6.34(5.18–7.71) | 3.26(2.62–3.99) | 8(6.38–9.93) | 54.82 | 0.44(0.14–0.75) |
| Uzbekistan | 1.98(1.36–2.8) | 1.53(1.05–2.17) | 5.26(3.51–7.48) | 1.8(1.22–2.5) | 166.34 | 0.43(0.32–0.54) |
| Vanuatu | 0.08(0.05–0.11) | 6.51(4.55–8.98) | 0.16(0.11–0.23) | 6.32(4.28–8.92) | 115.29 | -0.38(-0.5–-0.27) |
| Venezuela | 3.83(2.89–5.03) | 2.36(1.82–3.01) | 9.26(7.15–11.83) | 3.18(2.45–4.08) | 141.53 | 0.89(0.62–1.15) |
| Viet Nam | 5.17(3.28–7.55) | 1.08(0.69–1.56) | 21.81(13.93–31.26) | 1.98(1.29–2.83) | 321.65 | 2.21(2.11–2.31) |
| Yemen | 1.12(0.77–1.57) | 1.54(1.09–2.13) | 3.82(2.6–5.41) | 1.78(1.27–2.47) | 241.06 | 0.66(0.59–0.73) |
| Zambia | 0.61(0.43–0.84) | 1.63(1.13–2.22) | 2.05(1.45–2.82) | 2.15(1.52–2.94) | 236.28 | 1.08(0.96–1.2) |
| Zimbabwe | 1.45(1.02–2.03) | 2.74(1.92–3.76) | 3.06(2.11–4.36) | 3.16(2.19–4.41) | 110.86 | 0.41(0.36–0.45) |

EAPC: estimated annual percentage change; ASR, age-standardized rate; CI, confidence interval; UI: uncertainty interval.

**Table S8**. Global burden and trends in death and DALYs due to self-harm by firearm in sexes, SDI areas, and regions, 1990-2019.

| **Characteristics** | **Death** | | | | **DALYs** | | | |  |
| --- | --- | --- | --- | --- | --- | --- | --- | --- | --- |
|  | 2019 | | 1990-2019 | | 2019 | | 1990-2019 | | |
|  | Number  ×10^3^ (95% UI) | ASR/100,000  (95% UI) | % | EAPC  (95%CI) | Number  ×10^3^ (95% UI) | ASR/100,000  (95% UI) | % | EAPC  (95%CI) | |
| **Overall** | 52.69  (46.32–61.87) | 0.65  (0.57–0.76) | 2.84 | -1.97  (-2.07–-1.88) | 2267.65  (2001.29–2678.88) | 28.1  (24.84–33.19) | -6.46 | -2.03  (-2.13–-1.92) | |
| **Sex** |  |  |  |  |  |  |  |  | |
| Male | 45.11  (38.75–54.63) | 1.15  (0.99–1.39) | 3.14 | -2.07  (-2.17–-1.97) | 1864.78  (1583.6–2284.05) | 46.37  (39.39–56.87) | -7.11 | -2.1  (-2.22–-1.99) | |
| Female | 7.58  (6.84–8.39) | 0.19  (0.17–0.21) | 1.06 | -1.66  (-1.75–-1.57) | 402.86  (359.85–450.51) | 10.16  (9.06–11.38) | -3.31 | -1.66  (-1.75–-1.56) | |
| **SDI** |  |  |  |  |  |  |  |  | |
| Low | 4.19  (2.75–6.18) | 0.54  (0.35–0.79) | 92.82 | -0.49  (-0.66–-0.32) | 208.39  (141.21–305.41) | 22.78  (15.19–33.36) | 95.11 | -0.47  (-0.63–-0.31) | |
| Low-middle | 7.52  (5.31–10.35) | 0.43  (0.3–0.6) | 26.79 | -1.34  (-1.52–-1.16) | 404.73  (296.61–530.87) | 22.44  (16.28–29.82) | 22.12 | -1.33  (-1.52–-1.15) | |
| Middle | 6.04  (4.87–7.98) | 0.24  (0.19–0.31) | 2.96 | -2.07  (-2.29–-1.85) | 306.6  (253.36–403.58) | 12.03  (10.02–15.76) | -4.78 | -2.05  (-2.27–-1.83) | |
| High-middle | 6.54  (5.5–8.05) | 0.38  (0.33–0.47) | -21.09 | -3.12  (-3.62–-2.63) | 286.73  (244.45–349.78) | 18.15  (15.75–22.18) | -27.95 | -3.11  (-3.61–-2.61) | |
| High | 28.39  (26.14–32.29) | 2.16  (1.99–2.48) | -1.99 | -1.24  (-1.46–-1.01) | 1060.29  (977–1222) | 94.61  (87.19–109.5) | -16.18 | -1.4  (-1.66–-1.15) | |
| **Regions** |  |  |  |  |  |  |  |  | |
| East Asia | 0.51  (0.36–0.93) | 0.03  (0.02–0.05) | -58.64 | -4.41  (-4.62–-4.2) | 37.66  (29.35–55.33) | 2.16  (1.68–3.2) | -50.35 | -3.73  (-4.01–-3.45) | |
| South Asia | 7.22  (4.75–10.26) | 0.39  (0.25–0.57) | 15.45 | -1.79  (-1.95–-1.63) | 407.5  (292.15–555.6) | 21.73  (15.43–29.64) | 13.94 | -1.78  (-1.94–-1.61) | |
| Southeast Asia | 0.92  (0.55–1.41) | 0.13  (0.08–0.21) | -10.49 | -3.03  (-3.36–-2.69) | 44.25  (26.75–67.39) | 6.15  (3.73–9.37) | -19.88 | -3.33  (-3.73–-2.93) | |
| Central Asia | 0.15  (0.12–0.19) | 0.17  (0.14–0.21) | 28.76 | -1.43  (-2.32–-0.53) | 7.16  (5.73–8.76) | 7.51  (6.11–9.2) | 24.35 | -1.63  (-2.56–-0.69) | |
| High-income Asia Pacific | 0.07  (0.05–0.09) | 0.03  (0.02–0.04) | -61.62 | -4.44  (-4.56–-4.31) | 3.61  (3.11–4.52) | 1.64  (1.41–2.13) | -57.21 | -3.72  (-3.84–-3.6) | |
| Oceania | 0.04  (0.02–0.06) | 0.36  (0.18–0.52) | 43.7 | -1.53  (-1.59–-1.46) | 2.27  (1.22–3.35) | 17.94  (9.63–26.07) | 41.19 | -1.53  (-1.59–-1.47) | |
| Australasia | 0.22  (0.19–0.31) | 0.6  (0.52–0.85) | -60.8 | -5.35  (-5.87–-4.83) | 8.13  (7.03–11.46) | 25.2  (21.85–35.31) | -70.36 | -5.94  (-6.49–-5.39) | |
| Eastern Europe | 1.68  (1.33–2.32) | 0.67  (0.54–0.94) | -7.47 | -2.35  (-3.54–-1.13) | 76.02  (61.49–104.86) | 32.97  (26.89–46.24) | -11.88 | -2.33  (-3.54–-1.1) | |
| Western Europe | 4.93  (3.8–6.12) | 0.74  (0.56–0.92) | -43.28 | -3.58  (-3.71–-3.46) | 153.19  (118.16–189.76) | 28.6  (22.09–35.56) | -57.02 | -4.23  (-4.39–-4.07) | |
| Central Europe | 0.78  (0.57–0.99) | 0.52  (0.39–0.66) | -31.06 | -2.42  (-2.74–-2.09) | 30.15  (22.96–38.13) | 23.01  (17.57–29.39) | -44.46 | -2.91  (-3.24–-2.58) | |
| High-income North America | 24.06  (22.73–27.08) | 5.32  (5–6.02) | 17.36 | -0.6  (-0.83–-0.37) | 923.53  (867.43–1053.42) | 233.15  (218.84–266.18) | 1.26 | -0.8  (-1.06–-0.54) | |
| Andean Latin America | 0.18  (0.1–0.25) | 0.28  (0.15–0.4) | 20.43 | -2.24  (-2.6–-1.88) | 8.85  (5.04–12.79) | 13.6  (7.72–19.6) | 11.09 | -2.32  (-2.71–-1.94) | |
| Central Latin America | 2.29  (1.85–3.13) | 0.89  (0.72–1.22) | 34.48 | -1.8  (-2.13–-1.48) | 112.54  (90.34–154.27) | 42.85  (34.46–58.73) | 21.32 | -1.75  (-2.07–-1.42) | |
| Caribbean | 0.26  (0.2–0.39) | 0.52  (0.39–0.79) | -4.02 | -1.53  (-1.75–-1.31) | 12.21  (9.25–18.78) | 24.86  (18.83–38.31) | -11.61 | -1.59  (-1.82–-1.35) | |
| Tropical Latin America | 1.34  (1.16–1.78) | 0.55  (0.48–0.73) | -40.07 | -4.21  (-4.46–-3.95) | 62.31  (53.81–82.2) | 25.87  (22.33–34.06) | -46.73 | -4.2  (-4.46–-3.94) | |
| Southern Latin America | 1.49  (1.3–1.82) | 2  (1.76–2.47) | -4.00 | -2.59  (-3.1–-2.06) | 62.92  (55.18–79.69) | 88.8  (77.72–113.72) | -3.18 | -2.36  (-2.94–-1.78) | |
| Eastern sub-Saharan Africa | 1.64  (1–2.42) | 0.64  (0.41–0.97) | 115.19 | -0.31  (-0.55–-0.07) | 79  (47.65–118.73) | 24.88  (15.41–36.67) | 122.72 | -0.26  (-0.52–-0.01) | |
| Southern sub-Saharan Africa | 0.58  (0.32–0.92) | 0.76  (0.43–1.2) | 69.46 | -0.37  (-1.16–0.42) | 28.28  (15.25–45.93) | 34.78  (19.32–56.01) | 64.49 | -0.36  (-1.24–0.52) | |
| Western sub-Saharan Africa | 1.59  (1.05–2.47) | 0.6  (0.41–0.89) | 129.74 | 0.01  (-0.2–0.22) | 72.27  (46.74–114.68) | 21.33  (14.03–33.2) | 143.91 | 0.07  (-0.13–0.28) | |
| North Africa  and Middle East | 2.14  (1.32–2.84) | 0.36  (0.23–0.49) | 22.77 | -2.06  (-2.25–-1.87) | 108.39  (66.77–140.43) | 17.15  (10.67–22.35) | 14.41 | -2.18  (-2.39–-1.98) | |
| Central sub-Saharan Africa | 0.59  (0.35–0.89) | 0.75  (0.45–1.11) | 172.66 | 0.39  (0.12–0.66) | 27.4  (16.48–41.82) | 28.39  (16.93–42.29) | 182.75 | 0.53  (0.23–0.83) | |

DALYs: disability adjusted life years; EAPC: estimated annual percentage change; ASR, age-standardized rate; CI, confidence interval; UI: uncertainty interval; SDI: socio-demographic index.

**Table S9**. Burden and trends in death and DALYs due to self-harm by firearm at the national level from 1990 to 2019.

|  | **Death** | | | **DALYs** | | |
| --- | --- | --- | --- | --- | --- | --- |
| **Characteristics** | ASR/100,000  (95% UI) | Percent  (%) | EAPC  (95%CI) | ASR/100,000  (95% UI) | Percent (%) | EAPC  (95%CI) |
| Afghanistan | 0.37(0.22–0.61) | 192.52 | -0.1(-0.42–0.21) | 17.08(9.96–28.93) | 215.69 | -0.14(-0.48–0.2) |
| Albania | 0.89(0.51–1.24) | 4.89 | -0.47(-1.25–0.3) | 47.73(27.09–67.2) | -5.73 | -0.47(-1.29–0.37) |
| Algeria | 0.18(0.1–0.29) | 22.02 | -1.61(-1.7–-1.52) | 8.37(4.8–13.71) | 9.77 | -1.72(-1.79–-1.65) |
| American Samoa | 0.6(0.35–0.95) | -24.75 | -2.37(-2.71–-2.02) | 30.07(17.71–48.96) | -29.24 | -2.28(-2.62–-1.94) |
| Andorra | 0.42(0.15–1.55) | 2.32 | -2.43(-2.63–-2.23) | 16.94(6.06–62.99) | -16.11 | -2.52(-2.74–-2.3) |
| Angola | 0.66(0.38–1.01) | 141.08 | -0.66(-0.91–-0.41) | 24.72(14.04–37.94) | 146.04 | -0.62(-0.88–-0.36) |
| Antigua and Barbuda | 0.06(0.05–0.08) | 47.06 | -0.71(-0.92–-0.5) | 3.13(2.46–4.07) | 59.18 | -0.41(-0.71–-0.12) |
| Argentina | 2.44(2.13–3.02) | 10.3 | -2.19(-2.79–-1.59) | 109.59(95.3–141.73) | 18.33 | -1.88(-2.57–-1.19) |
| Armenia | 0.42(0.31–0.54) | 136.71 | 2.62(1.71–3.53) | 18.29(11.61–23.45) | 91.6 | 2.21(1.15–3.28) |
| Australia | 0.58(0.49–0.83) | -62.84 | -5.61(-6.18–-5.04) | 24.02(20.27–34.01) | -72.21 | -6.26(-6.87–-5.64) |
| Austria | 1.55(1.06–1.81) | -26.39 | -2.87(-3.13–-2.61) | 55.36(36.51–64.85) | -46.36 | -3.69(-3.95–-3.42) |
| Azerbaijan | 0.09(0.05–0.13) | 44.93 | -0.3(-0.95–0.34) | 3.87(1.99–5.47) | 36.37 | -0.9(-1.62–-0.18) |
| Bahamas | 0.19(0.14–0.26) | 45.95 | -0.85(-1.07–-0.64) | 9.53(7.23–12.81) | 43.17 | -0.46(-0.65–-0.27) |
| Bahrain | 0.1(0.06–0.16) | 92.48 | -2.64(-2.84–-2.45) | 4.52(2.64–7.03) | 89.92 | -2.29(-2.52–-2.05) |
| Bangladesh | 0.15(0.08–0.26) | -39.96 | -3.98(-4.09–-3.87) | 7.62(4.62–13.24) | -42.65 | -3.91(-4.02–-3.8) |
| Barbados | 0.2(0.15–0.25) | -6.72 | -2.1(-2.43–-1.77) | 9.21(6.82–11.84) | -15.92 | -2.18(-2.54–-1.82) |
| Belarus | 0.41(0.28–0.62) | -7.1 | -2.4(-4.02–-0.76) | 17.79(12.46–27.01) | -21.18 | -2.74(-4.37–-1.09) |
| Belgium | 1.1(0.88–1.43) | -41.08 | -3.58(-3.96–-3.19) | 43.47(35.56–57.66) | -52.64 | -4.11(-4.52–-3.7) |
| Belize | 0.76(0.61–0.98) | 381.98 | 0.68(-0.23–1.6) | 36.67(29.06–47.76) | 377.82 | 0.77(-0.13–1.67) |
| Benin | 0.77(0.46–1.21) | 180.66 | 0.16(-0.04–0.36) | 28.16(15.73–45.35) | 203.19 | 0.31(0.09–0.52) |
| Bermuda | 0.04(0.03–0.05) | -66.14 | -4.28(-4.69–-3.87) | 1.89(1.34–2.4) | -68.12 | -3.93(-4.35–-3.52) |
| Bhutan | 0.17(0.09–0.33) | -18.55 | -2.56(-2.66–-2.47) | 8.65(4.89–16.25) | -19.62 | -2.49(-2.59–-2.38) |
| Bolivia | 0.44(0.23–0.65) | 11.95 | -2.58(-2.73–-2.42) | 18.74(10.32–28.13) | 1.27 | -2.77(-2.93–-2.62) |
| Bosnia and Herzegovina | 0.59(0.25–0.82) | -15.22 | -0.36(-0.76–0.04) | 25.75(11.9–35.38) | -31.3 | -0.64(-1.07–-0.2) |
| Botswana | 0.98(0.48–1.7) | 190.04 | -0.31(-1.25–0.63) | 42.33(21.4–75.95) | 194.69 | -0.18(-1.18–0.83) |
| Brazil | 0.53(0.46–0.72) | -43.11 | -4.38(-4.64–-4.12) | 24.8(21.39–33.69) | -49.7 | -4.38(-4.65–-4.11) |
| Brunei Darussalam | 0.05(0.03–0.07) | 17.52 | -1.18(-1.66–-0.69) | 2.38(1.42–3.42) | 7.51 | -1.57(-2.09–-1.06) |
| Bulgaria | 0.76(0.41–1.01) | 8.63 | -0.47(-1.04–0.1) | 36.48(19.92–48.59) | -9.96 | -0.87(-1.41–-0.32) |
| Burkina Faso | 0.94(0.63–1.42) | 154.25 | 0.15(0.07–0.23) | 33.64(22.08–55.7) | 185.41 | 0.44(0.36–0.53) |
| Burundi | 0.82(0.45–1.53) | 112.56 | -0.43(-0.62–-0.23) | 32.85(17.55–62.97) | 123.55 | -0.37(-0.58–-0.17) |
| Cabo Verde | 1.06(0.59–1.51) | 82.05 | -0.84(-1.15–-0.53) | 42.45(22.21–61.41) | 63.52 | -1.05(-1.36–-0.73) |
| Cambodia | 0.1(0.06–0.2) | 9.31 | -2.17(-2.22–-2.13) | 4.42(2.86–8.79) | 1.2 | -2.3(-2.35–-2.26) |
| Cameroon | 0.85(0.47–1.28) | 260.3 | 0.64(0.23–1.05) | 31.34(16.92–48.88) | 288.21 | 0.84(0.42–1.26) |
| Canada | 1.47(1.24–1.9) | -41.26 | -3.72(-4.22–-3.23) | 65.41(54.41–86.16) | -53.92 | -4.12(-4.69–-3.55) |
| Central African Republic | 1.47(0.68–2.62) | 161.42 | 0.94(0.69–1.19) | 59.23(25.35–110.09) | 173.46 | 1.06(0.79–1.34) |
| Chad | 0.98(0.58–1.53) | 209.09 | 1.02(0.58–1.47) | 35.27(20.25–57.28) | 246.68 | 1.18(0.72–1.64) |
| Chile | 0.57(0.41–0.69) | -60.74 | -5.44(-5.73–-5.14) | 25.22(18.7–31.03) | -66.38 | -5.62(-5.93–-5.3) |
| China | 0.03(0.02–0.05) | -60.22 | -4.57(-4.78–-4.36) | 2.04(1.58–3.1) | -52.06 | -3.89(-4.17–-3.61) |
| Colombia | 0.93(0.67–1.35) | -13.25 | -2.88(-3.42–-2.35) | 47.53(33.9–68.01) | -22.2 | -2.91(-3.43–-2.39) |
| Comoros | 0.45(0.22–0.9) | 110.63 | -0.37(-0.68–-0.05) | 17.44(7.77–36.23) | 110.32 | -0.3(-0.74–0.15) |
| Congo | 0.64(0.38–0.96) | 70.68 | -1.49(-1.65–-1.32) | 23.36(14.02–35.79) | 64.69 | -1.72(-1.91–-1.53) |
| Cook Islands | 0.46(0.22–0.71) | -39.5 | -1.92(-2.37–-1.48) | 23.03(11.11–36.23) | -45.83 | -1.85(-2.3–-1.4) |
| Costa Rica | 1.16(0.85–1.66) | 71.85 | -1.51(-1.97–-1.05) | 52.59(38.54–76.06) | 58.37 | -1.42(-1.91–-0.92) |
| Croatia | 1.34(0.87–1.77) | -50.51 | -4.36(-4.9–-3.82) | 58.48(36.24–76.6) | -61.72 | -5.05(-5.64–-4.47) |
| Cuba | 0.2(0.14–0.26) | -65.09 | -4.98(-5.4–-4.55) | 9.8(6.39–12.65) | -73.7 | -5.52(-5.98–-5.06) |
| Cyprus | 0.64(0.25–0.8) | 43.65 | -1.24(-1.4–-1.09) | 29.83(10.44–38.8) | 41.18 | -1.09(-1.26–-0.91) |
| Czechia | 0.92(0.63–1.23) | -2.04 | -1.21(-1.73–-0.7) | 37.35(27.18–50.22) | -20.9 | -1.79(-2.36–-1.21) |
| Côte d'Ivoire | 1.02(0.57–1.69) | 140.57 | -0.31(-0.64–0.02) | 35.96(19.87–62.46) | 137.57 | -0.22(-0.57–0.12) |
| DPR Korea | 0.1(0.05–0.21) | -8.51 | -1.5(-1.54–-1.46) | 6.06(3.78–11.99) | -9.01 | -1.54(-1.61–-1.47) |
| DR Congo | 0.75(0.46–1.21) | 201.5 | 0.87(0.56–1.18) | 28.33(17.12–46.43) | 215.58 | 1.1(0.76–1.45) |
| Denmark | 0.66(0.47–0.81) | -67.71 | -4.88(-5.14–-4.62) | 26.44(18.7–32.49) | -72.61 | -5.27(-5.53–-5.01) |
| Djibouti | 0.54(0.29–0.93) | 347.17 | 0.9(0.51–1.29) | 20.41(10.1–37.44) | 318.88 | 1.03(0.59–1.48) |
| Dominica | 0.15(0.09–0.23) | -1.72 | -0.66(-0.81–-0.51) | 7.38(4.58–11.23) | -4.76 | -0.55(-0.74–-0.35) |
| Dominican Republic | 0.89(0.48–1.61) | 116.86 | 1.37(0.96–1.78) | 41.47(22.77–76.97) | 98 | 1.29(0.89–1.69) |
| Ecuador | 0.52(0.3–0.8) | 34.47 | -2.31(-2.91–-1.7) | 25.18(15.33–39.28) | 23.77 | -2.46(-3.11–-1.81) |
| Egypt | 0.22(0.11–0.41) | 91.57 | 0.52(0.29–0.75) | 9.36(4.7–17.08) | 80.02 | 0.42(0.17–0.67) |
| El Salvador | 0.15(0.09–0.28) | -52.18 | -4.11(-4.67–-3.55) | 7.97(4.95–14.6) | -55.75 | -4.04(-4.61–-3.47) |
| Equatorial Guinea | 0.43(0.23–0.72) | 37.34 | -3.21(-3.63–-2.79) | 15.71(8.39–27.46) | 53.76 | -3.35(-3.78–-2.91) |
| Eritrea | 0.94(0.5–1.48) | 228.96 | 0.84(0.52–1.17) | 38.3(18.43–63.05) | 235.82 | 0.96(0.6–1.32) |
| Estonia | 0.71(0.4–1) | -64.56 | -5.16(-5.85–-4.46) | 32.26(18.04–45.78) | -71.14 | -5.58(-6.26–-4.89) |
| Eswatini | 1.44(0.53–2.9) | 240.28 | 2.59(1.6–3.59) | 64.83(23.99–129.1) | 259.53 | 3.09(2–4.2) |
| Ethiopia | 0.53(0.34–0.95) | 9.16 | -2.58(-2.79–-2.36) | 19.93(13.09–33.39) | 11.98 | -2.75(-2.98–-2.51) |
| Fiji | 0.05(0.03–0.09) | -13.62 | -1.55(-1.76–-1.34) | 3.44(2.55–5.43) | -4.18 | -1.05(-1.26–-0.84) |
| Finland | 2.01(1.71–2.73) | -67.96 | -5.28(-5.55–-5.01) | 87.86(74.87–125.31) | -75.56 | -5.72(-5.99–-5.44) |
| France | 1.7(1.28–2.07) | -49.86 | -4.25(-4.46–-4.04) | 61.98(46.21–74.92) | -64.09 | -5.02(-5.26–-4.77) |
| Gabon | 0.64(0.31–1.15) | 82.49 | -0.17(-0.43–0.1) | 23.55(11.22–42.7) | 84.28 | -0.25(-0.54–0.03) |
| Gambia | 0.73(0.37–1.28) | 231.88 | 0.66(0.42–0.91) | 26.19(13.24–46.5) | 235.86 | 0.85(0.56–1.13) |
| Georgia | 0.61(0.48–0.82) | 11.92 | 1.38(1.11–1.65) | 29.61(22.77–39.03) | 1.94 | 1.17(0.84–1.49) |
| Germany | 0.6(0.44–0.73) | -32.87 | -2.88(-3.02–-2.73) | 20.5(14.75–24.57) | -49.97 | -3.7(-3.89–-3.51) |
| Ghana | 0.44(0.28–0.66) | 112.12 | -0.18(-0.44–0.07) | 15.65(9.79–25.03) | 109.33 | -0.21(-0.45–0.02) |
| Greece | 0.66(0.55–0.83) | 17.9 | -0.01(-0.23–0.2) | 28.18(23.55–34.87) | -5.96 | -0.31(-0.56–-0.05) |
| Greenland | 14.11(10.24–21.67) | -41.11 | -2.43(-2.5–-2.37) | 596.86(422.98–886.94) | -52.48 | -2.77(-2.86–-2.69) |
| Grenada | 0.03(0.03–0.04) | -42.13 | -3.27(-3.45–-3.08) | 2.05(1.51–2.45) | -35.85 | -2.91(-3.12–-2.7) |
| Guam | 1.46(0.85–2.15) | -19.29 | -2.09(-2.52–-1.65) | 74.06(42.89–108.3) | -26.32 | -2(-2.39–-1.6) |
| Guatemala | 0.39(0.24–0.51) | -21.91 | -5.95(-7.45–-4.42) | 18.74(11.82–24.66) | -20.75 | -5.86(-7.38–-4.33) |
| Guinea | 0.89(0.48–1.54) | 145.86 | 1.26(1.05–1.46) | 32.5(17.14–56.1) | 170.55 | 1.42(1.21–1.63) |
| Guinea–Bissau | 1.24(0.67–1.91) | 86.08 | -0.04(-0.24–0.16) | 46.8(25.4–73.73) | 97.05 | 0.08(-0.13–0.3) |
| Guyana | 0.39(0.27–0.6) | 6.79 | -0.08(-0.56–0.41) | 19.25(13.46–30.54) | 3.32 | 0.15(-0.38–0.68) |
| Haiti | 0.55(0.32–0.96) | 20.68 | -1.93(-2.18–-1.67) | 25.71(15.27–45.26) | 19.47 | -1.96(-2.24–-1.67) |
| Honduras | 0.49(0.33–0.95) | -12.1 | -3.18(-3.44–-2.91) | 16.71(10.43–37.12) | -32.42 | -4.36(-4.59–-4.14) |
| Hungary | 0.35(0.18–0.46) | -39.01 | -2.87(-3.32–-2.43) | 15.27(8.27–19.86) | -51.84 | -3.56(-4–-3.13) |
| Iceland | 0.87(0.64–1.08) | -36.86 | -3.21(-3.35–-3.06) | 34.25(24.8–42.34) | -48.46 | -3.48(-3.64–-3.32) |
| India | 0.43(0.27–0.63) | 13.59 | -1.79(-1.97–-1.6) | 23.65(16.74–32.9) | 11.86 | -1.79(-1.97–-1.6) |
| Indonesia | 0.06(0.04–0.12) | 2.24 | -1.39(-1.5–-1.27) | 2.64(1.67–5.01) | -9.24 | -1.77(-1.9–-1.63) |
| Iran | 0.3(0.17–0.36) | 16.68 | -1.15(-1.31–-0.99) | 15.73(8.95–18.83) | 5.57 | -1.12(-1.28–-0.96) |
| Iraq | 1.22(0.75–1.73) | 125.23 | -1.16(-1.33–-1) | 51.78(32.8–80.07) | 116.13 | -1.38(-1.57–-1.19) |
| Ireland | 0.34(0.25–0.73) | -48.02 | -4.46(-4.96–-3.96) | 17.5(12.44–39.38) | -52.41 | -4.7(-5.24–-4.16) |
| Israel | 1.01(0.74–1.18) | 14.62 | -3.15(-3.85–-2.44) | 45.93(33.94–54.08) | 2.6 | -3.43(-4.25–-2.6) |
| Italy | 0.53(0.42–0.69) | -31.23 | -2.71(-2.91–-2.51) | 21.65(16.94–27.53) | -43.51 | -3.02(-3.24–-2.8) |
| Jamaica | 0.55(0.4–0.75) | 1809.27 | 11.52(9.9–13.15) | 25.74(19.09–35.73) | 1689.38 | 11.09(9.53–12.68) |
| Japan | 0.03(0.02–0.04) | -68.07 | -4.59(-4.71–-4.46) | 1.55(1.39–2.21) | -62.53 | -3.77(-3.88–-3.66) |
| Jordan | 0.1(0.07–0.16) | 91.26 | -2.7(-2.84–-2.56) | 4.14(2.97–7.1) | 82.4 | -2.8(-2.96–-2.63) |
| Kazakhstan | 0.25(0.19–0.33) | 9.13 | -1.67(-3.33–0.01) | 11.45(8.9–15.3) | 5.87 | -1.61(-3.35–0.16) |
| Kenya | 0.6(0.36–0.94) | 316.17 | 1.65(1.08–2.22) | 22.43(13.12–37) | 333.14 | 2.02(1.37–2.67) |
| Kiribati | 0.2(0.13–0.34) | 16.72 | -1.37(-1.48–-1.26) | 11.39(7.74–18.68) | 19.26 | -1.26(-1.35–-1.16) |
| Kuwait | 0.07(0.06–0.1) | 134.98 | -0.94(-1.39–-0.49) | 3.05(2.32–4.05) | 116.8 | -1.26(-1.67–-0.86) |
| Kyrgyzstan | 0.12(0.09–0.15) | -35.15 | -4.47(-5.14–-3.79) | 5.14(4.02–6.8) | -34.47 | -4.46(-5.12–-3.8) |
| Lao People's Democratic Republic | 0.13(0.08–0.3) | -24.74 | -3.54(-3.62–-3.47) | 6.25(3.58–13.63) | -27.46 | -3.68(-3.76–-3.6) |
| Latvia | 0.63(0.4–0.89) | -61.38 | -4.54(-5.33–-3.75) | 29.05(18.9–40.38) | -65.77 | -4.73(-5.51–-3.94) |
| Lebanon | 0.16(0.08–0.26) | 47.64 | -0.42(-0.8–-0.03) | 7.83(4.16–13.09) | 41.42 | -0.34(-0.76–0.09) |
| Lesotho | 2.32(0.51–5.91) | 260.19 | 4.37(3.69–5.05) | 101.17(24.1–266.02) | 299.36 | 5.04(4.27–5.82) |
| Liberia | 0.7(0.33–1.39) | 131.69 | -0.26(-0.47–-0.06) | 25.5(11.42–51.19) | 173.08 | -0.07(-0.27–0.13) |
| Libya | 0.22(0.13–0.33) | 134.33 | 0.51(0.26–0.75) | 10.47(6.21–16.13) | 116.78 | 0.5(0.23–0.78) |
| Lithuania | 0.47(0.32–0.66) | -37.21 | -2.56(-3.4–-1.71) | 22.06(15.22–30.39) | -46.15 | -2.84(-3.7–-1.98) |
| Luxembourg | 0.77(0.49–0.95) | -40.33 | -4.33(-4.6–-4.06) | 32.27(19.69–40.06) | -45.94 | -4.63(-5–-4.26) |
| Madagascar | 0.6(0.36–0.95) | 131.85 | 0.29(0.09–0.5) | 23.75(14.04–38.48) | 127.3 | 0.24(0.02–0.45) |
| Malawi | 0.83(0.48–1.39) | 146.45 | 0.53(0.07–1) | 32.42(17.29–55.36) | 156.62 | 0.69(0.18–1.2) |
| Malaysia | 0.13(0.06–0.19) | 85.61 | -0.57(-0.84–-0.29) | 6.14(3.06–9.17) | 76.89 | -0.61(-0.9–-0.31) |
| Maldives | 0.09(0.05–0.13) | 22.43 | -4.06(-4.57–-3.54) | 4.04(2.43–6.29) | 20.8 | -3.89(-4.42–-3.35) |
| Mali | 0.73(0.35–1.47) | 111.29 | -0.39(-0.44–-0.34) | 25.96(12.09–53.88) | 126.73 | -0.38(-0.46–-0.3) |
| Malta | 0.38(0.28–0.48) | -9.2 | -2.14(-2.29–-1.99) | 16.52(11.65–20.64) | -18.05 | -1.99(-2.2–-1.77) |
| Marshall Islands | 1.16(0.51–1.91) | -1.68 | -1.49(-1.63–-1.36) | 60.82(27.2–101.25) | -7.94 | -1.51(-1.66–-1.37) |
| Mauritania | 0.45(0.23–0.88) | 27.14 | -1.49(-1.57–-1.4) | 14.74(7–31.44) | 26.19 | -1.5(-1.62–-1.38) |
| Mauritius | 0.08(0.06–0.11) | -7.77 | -1.42(-1.95–-0.88) | 3.81(2.82–5.15) | -14.18 | -1.14(-1.67–-0.6) |
| Mexico | 0.72(0.57–1.14) | 37.73 | -1.92(-2.25–-1.6) | 32.13(25.09–53.79) | 18.85 | -2.02(-2.39–-1.65) |
| Micronesia | 1.28(0.44–2.22) | -19.56 | -1.55(-1.72–-1.37) | 67(23.42–116.3) | -21.84 | -1.51(-1.67–-1.35) |
| Monaco | 1.81(1.3–2.8) | 2.72 | -0.57(-0.64–-0.5) | 77.68(54.74–129.98) | -6.29 | -0.67(-0.73–-0.61) |
| Mongolia | 0.6(0.25–0.85) | 77.66 | -1.46(-1.98–-0.93) | 29.21(11.09–42.48) | 71.85 | -1.41(-1.95–-0.88) |
| Montenegro | 2.75(1.89–4.44) | -1.17 | -0.67(-1.03–-0.31) | 120.36(83.48–197.59) | -20.85 | -1.12(-1.53–-0.71) |
| Morocco | 0.27(0.12–0.5) | 50.37 | -0.31(-0.5–-0.12) | 12.04(5.9–21.85) | 35.1 | -0.49(-0.72–-0.25) |
| Mozambique | 1.05(0.52–1.53) | 275.84 | 2.58(2.28–2.89) | 41.43(19.99–61.48) | 312.49 | 2.94(2.61–3.28) |
| Myanmar | 0.09(0.05–0.17) | -46.96 | -3.9(-4.07–-3.73) | 4.18(2.42–8) | -50.43 | -3.97(-4.13–-3.8) |
| Namibia | 0.76(0.44–1.21) | 122.48 | 0(-0.67–0.67) | 31.5(18.5–53.03) | 137.29 | 0.27(-0.49–1.04) |
| Nauru | 1.28(0.35–2.5) | -16.18 | -1.23(-1.66–-0.79) | 66.68(18.64–133.38) | -15.41 | -1.12(-1.54–-0.69) |
| Nepal | 0.46(0.24–0.73) | -4.09 | -2.25(-2.54–-1.97) | 22.15(12.24–34.25) | -5.78 | -2.28(-2.56–-2) |
| Netherlands | 0.2(0.16–0.29) | -9.84 | -1.62(-1.91–-1.32) | 9.01(7.46–13.19) | -22.14 | -1.84(-2.13–-1.55) |
| New Zealand | 0.71(0.6–0.99) | -46.94 | -3.99(-4.28–-3.71) | 31.79(26.81–45.39) | -58.03 | -4.38(-4.68–-4.08) |
| Nicaragua | 0.35(0.22–0.61) | 35.87 | -2.4(-2.65–-2.16) | 14.16(9.17–23.95) | 22.06 | -2.52(-2.76–-2.28) |
| Niger | 0.78(0.41–1.47) | 184.1 | 0(-0.11–0.11) | 27.99(13.46–55.14) | 194.95 | 0.09(-0.02–0.2) |
| Nigeria | 0.38(0.23–0.67) | 83.95 | -0.51(-0.75–-0.27) | 13.15(8.05–23.6) | 96.85 | -0.56(-0.79–-0.33) |
| Niue | 0.49(0.22–0.78) | -56.01 | -2.79(-2.95–-2.63) | 24.69(11.14–40.78) | -59.16 | -2.83(-3–-2.67) |
| North Macedonia | 0.73(0.37–1.05) | 32.91 | 0(-0.32–0.32) | 32.67(17.28–47.83) | 17.77 | -0.17(-0.51–0.18) |
| Northern Mariana Islands | 1.01(0.53–1.51) | -42.15 | -2.42(-2.71–-2.12) | 48.97(25.7–74.32) | -51.38 | -2.34(-2.66–-2.02) |
| Norway | 1.11(0.97–1.73) | -65.7 | -4.73(-4.93–-4.52) | 51(43.82–83.76) | -70.51 | -5.08(-5.31–-4.85) |
| Oman | 0.05(0.03–0.1) | 31.86 | -2.24(-2.42–-2.05) | 2.25(1.42–4.39) | 42.15 | -2.39(-2.6–-2.17) |
| Pakistan | 0.36(0.19–0.69) | 122.23 | -0.4(-0.71–-0.08) | 19.63(11.28–34.91) | 126.27 | -0.28(-0.57–0) |
| Palau | 0.74(0.38–1.08) | 10.81 | -0.62(-0.7–-0.54) | 38.03(19.35–55.11) | -0.19 | -0.54(-0.61–-0.46) |
| Palestine | 0.09(0.05–0.11) | 73.14 | -1.59(-1.91–-1.27) | 3.56(2.22–4.58) | 71.32 | -1.79(-2.16–-1.42) |
| Panama | 0.18(0.12–0.24) | -30.78 | -5.16(-5.97–-4.36) | 7.98(5.64–10.95) | -36.49 | -5(-5.78–-4.22) |
| Papua New Guinea | 0.21(0.12–0.36) | 90.07 | -0.91(-0.99–-0.84) | 10.39(5.99–17.82) | 89.22 | -0.94(-1.01–-0.86) |
| Paraguay | 1.26(0.75–1.74) | 177.55 | 0.71(0.33–1.09) | 59.11(35.87–81.54) | 164.35 | 0.67(0.27–1.07) |
| Peru | 0.12(0.07–0.18) | 5.41 | -2.08(-2.35–-1.81) | 6.07(3.56–9.2) | -0.98 | -2.03(-2.29–-1.77) |
| Philippines | 0.11(0.06–0.15) | 49.02 | -0.82(-1.2–-0.43) | 5.09(2.68–7.23) | 37.89 | -0.93(-1.33–-0.53) |
| Poland | 0.12(0.09–0.2) | -39.73 | -3.2(-3.68–-2.71) | 6.28(4.93–10.19) | -45.89 | -3.42(-3.84–-2.99) |
| Portugal | 0.91(0.74–1.17) | 15.56 | -0.52(-1.12–0.08) | 35.18(25.34–42.81) | -18.19 | -1.32(-1.93–-0.71) |
| Puerto Rico | 0.65(0.47–0.89) | -58.04 | -4.37(-4.77–-3.97) | 28.88(20.76–39.66) | -63.26 | -4.42(-4.83–-4.01) |
| Qatar | 0.05(0.03–0.12) | 254.88 | -3(-3.24–-2.76) | 2.4(1.46–4.75) | 289.27 | -2.85(-3.1–-2.59) |
| Republic of Korea | 0.03(0.02–0.05) | -35.71 | -3.42(-3.74–-3.09) | 1.88(1.11–2.62) | -40.21 | -3.2(-3.49–-2.91) |
| Republic of Moldova | 0.18(0.13–0.24) | -46.29 | -2.11(-2.59–-1.62) | 9.43(7.23–12.45) | -50.03 | -2.25(-2.7–-1.8) |
| Romania | 0.05(0.04–0.06) | -52.96 | -3.43(-4–-2.85) | 2.84(2.22–3.67) | -54.41 | -3.34(-3.88–-2.79) |
| Russian Federation | 0.6(0.45–0.88) | -12.13 | -2.58(-3.92–-1.22) | 29.19(22.66–44.09) | -18.53 | -2.71(-4.1–-1.31) |
| Rwanda | 0.54(0.31–0.9) | 37.74 | -2.56(-2.97–-2.15) | 21.23(11.56–37.58) | 39.55 | -2.62(-3.06–-2.18) |
| Saint Kitts and Nevis | 0.06(0.04–0.09) | -28.58 | -3.13(-3.57–-2.68) | 2.77(1.69–3.87) | -28.85 | -2.84(-3.37–-2.31) |
| Saint Lucia | 0.26(0.17–0.36) | 39.25 | -1.34(-1.63–-1.04) | 12.05(8.55–16.65) | 31.18 | -1.1(-1.33–-0.87) |
| Saint Vincent and the Grenadines | 0.14(0.1–0.17) | 8.46 | -1.14(-1.32–-0.96) | 6.69(5.1–8.27) | -2.07 | -1.09(-1.29–-0.9) |
| Samoa | 0.57(0.27–0.97) | -10.22 | -1.69(-1.81–-1.57) | 29.54(13.94–50.27) | -11.43 | -1.59(-1.69–-1.48) |
| San Marino | 2.86(1.68–4.76) | 25.46 | -1.03(-1.29–-0.77) | 113.7(67.83–195.81) | 7.18 | -1.14(-1.41–-0.88) |
| Sao Tome and Principe | 0.19(0.08–0.34) | 158.26 | 0.67(0.37–0.97) | 8.17(3.35–14.43) | 188.88 | 0.84(0.46–1.22) |
| Saudi Arabia | 0.26(0.13–0.37) | 246.45 | 0.86(0.43–1.28) | 11.71(5.93–17.19) | 238.59 | 1.05(0.58–1.52) |
| Senegal | 0.87(0.54–1.39) | 124.38 | 0.08(-0.12–0.27) | 30.78(17.57–51.99) | 130.02 | 0.27(0.03–0.5) |
| Serbia | 1.78(1.21–2.94) | -39.14 | -2.6(-2.85–-2.35) | 73.04(50.5–122.83) | -54.31 | -3.35(-3.61–-3.08) |
| Seychelles | 0.14(0.08–0.29) | 31.79 | -1.78(-2.08–-1.47) | 6.35(3.61–13.1) | 26.59 | -1.72(-2.08–-1.35) |
| Sierra Leone | 0.73(0.4–1.26) | 153.59 | 0.72(0.45–0.98) | 26.77(14.37–46.78) | 190.11 | 1(0.73–1.28) |
| Singapore | 0.02(0.01–0.03) | -65.63 | -6.69(-7.15–-6.23) | 1.21(0.85–1.44) | -63.64 | -6.16(-6.71–-5.6) |
| Slovakia | 0.73(0.43–1.1) | -46.53 | -2.89(-3.1–-2.67) | 33.97(20.27–51.86) | -52.58 | -3.1(-3.31–-2.88) |
| Slovenia | 1.02(0.67–1.45) | -34.98 | -3.03(-3.51–-2.54) | 41.92(27.4–58.99) | -50.84 | -3.55(-4.06–-3.05) |
| Solomon Islands | 2.02(0.64–3.87) | 61.08 | -1.01(-1.08–-0.95) | 105.35(34.45–200.99) | 59.62 | -0.97(-1.04–-0.91) |
| Somalia | 1.06(0.54–2.14) | 301.27 | 1.13(0.89–1.36) | 43.38(21.41–90.78) | 337.85 | 1.34(1.08–1.6) |
| South Africa | 0.68(0.36–1.05) | 45.81 | -1.03(-1.94–-0.11) | 32.44(16.5–52.81) | 40.05 | -1.09(-2.08–-0.08) |
| South Sudan | 0.53(0.3–0.95) | 84.57 | 0.39(0.21–0.58) | 19.6(10.46–36.77) | 92.44 | 0.51(0.31–0.7) |
| Spain | 0.33(0.24–0.42) | -11.41 | -1.85(-2.07–-1.63) | 14.77(10.18–17.91) | -28.2 | -2.36(-2.62–-2.1) |
| Sri Lanka | 0.08(0.05–0.14) | -66.8 | -6.01(-6.8–-5.22) | 3.94(2.45–6.51) | -71.16 | -6.28(-7.12–-5.43) |
| Sudan | 0.33(0.17–0.53) | 73.3 | -0.67(-0.82–-0.52) | 15.63(7.73–25.6) | 76.42 | -0.62(-0.79–-0.46) |
| Suriname | 1.04(0.68–1.69) | 31.76 | -1.21(-1.54–-0.88) | 49.21(32.13–79.15) | 17.7 | -1.31(-1.66–-0.95) |
| Sweden | 0.85(0.7–1.17) | -42.6 | -3.08(-3.21–-2.96) | 32.91(27.73–46.36) | -53.19 | -3.45(-3.58–-3.33) |
| Switzerland | 1.69(1.23–1.99) | -62.62 | -5.47(-5.67–-5.28) | 66.49(47.16–78.31) | -72.78 | -6.31(-6.56–-6.05) |
| Syrian Arab Republic | 0.12(0.08–0.19) | -14.15 | -1.87(-2.19–-1.55) | 5.5(3.29–8.22) | -23.76 | -1.98(-2.35–-1.6) |
| Taiwan  (Province of China) | 0.06(0.04–0.09) | -49.57 | -3.06(-3.63–-2.49) | 4.77(3.72–6.66) | -29.33 | -1.95(-2.43–-1.47) |
| Tajikistan | 0.04(0.03–0.05) | 62.21 | -0.89(-1.26–-0.52) | 1.6(1.1–2.34) | 68.96 | -1.41(-1.86–-0.95) |
| Thailand | 0.47(0.23–0.7) | -23.93 | -3.77(-4.42–-3.12) | 23.76(11.56–36.15) | -32.97 | -3.92(-4.65–-3.18) |
| Timor–Leste | 0.11(0.05–0.27) | 2.91 | -1.99(-2.44–-1.54) | 4.75(1.99–11.77) | -3.91 | -2.25(-2.77–-1.74) |
| Togo | 0.84(0.49–1.27) | 224.39 | 0.71(0.4–1.02) | 31.12(17.79–48.69) | 219.81 | 0.92(0.6–1.23) |
| Tokelau | 0.39(0.19–0.8) | -48.94 | -2.15(-2.23–-2.08) | 19.96(9.02–42.9) | -50.59 | -2.14(-2.21–-2.07) |
| Tonga | 0.2(0.1–0.39) | 4.05 | -0.39(-0.49–-0.29) | 10.41(5.04–19.71) | 5.64 | -0.15(-0.25–-0.05) |
| Trinidad and Tobago | 0.13(0.09–0.21) | -19.93 | -2.52(-3.02–-2.02) | 6.69(4.69–11.14) | -21.95 | -2.14(-2.64–-1.64) |
| Tunisia | 0.15(0.09–0.26) | 67.34 | -0.19(-0.3–-0.09) | 7.12(4.19–12.36) | 49.83 | -0.14(-0.25–-0.03) |
| Turkey | 0.64(0.4–0.9) | -34.86 | -3.85(-4.27–-3.43) | 32.75(19.27–44.68) | -39.78 | -3.84(-4.28–-3.4) |
| Turkmenistan | 0.12(0.09–0.2) | 27.4 | -2.09(-3.29–-0.87) | 6.54(4.57–10.97) | 26.88 | -1.89(-3.17–-0.6) |
| Tuvalu | 0.83(0.36–1.39) | -15.7 | -1.4(-1.56–-1.24) | 42.87(18.53–75.11) | -15.05 | -1.31(-1.45–-1.17) |
| Uganda | 0.46(0.23–1) | 225.19 | 0.12(-0.51–0.75) | 17.76(9.68–32.11) | 261.55 | 0.35(-0.34–1.05) |
| Ukraine | 1.02(0.72–1.35) | 17.14 | -1.56(-2.88–-0.22) | 51.51(36.56–68.16) | 22.64 | -1.06(-2.36–0.26) |
| United Arab Emirates | 0.33(0.14–0.6) | 587.72 | -0.43(-0.52–-0.34) | 14.11(6.4–27.73) | 537.1 | -0.23(-0.36–-0.11) |
| United Kingdom | 0.14(0.12–0.22) | -51.97 | -3.73(-3.93–-3.52) | 6.42(5.69–10.9) | -56.79 | -3.95(-4.18–-3.72) |
| United Republic of Tanzania | 0.57(0.35–0.94) | 152.12 | 0.27(0.07–0.47) | 21.71(12.62–35.98) | 155.2 | 0.34(0.13–0.55) |
| United States of America | 5.76(5.42–6.5) | 20.95 | -0.46(-0.67–-0.24) | 251.58(237.06–285.68) | 4.97 | -0.65(-0.9–-0.4) |
| United States Virgin Islands | 1.77(1.14–2.67) | 2.3 | -1.42(-1.67–-1.17) | 71.77(46.28–111.57) | -24.22 | -2.21(-2.69–-1.73) |
| Uruguay | 3.8(3.36–4.83) | 7.12 | -1.3(-1.89–-0.71) | 154.86(134.4–199.38) | -2.67 | -1.42(-2.02–-0.82) |
| Uzbekistan | 0.06(0.05–0.09) | 87.8 | -1(-1.78–-0.21) | 2.8(2.2–4.27) | 97.41 | -0.95(-1.72–-0.17) |
| Vanuatu | 1.16(0.49–1.9) | 78.35 | -1.13(-1.3–-0.96) | 60.27(25.69–99.37) | 77.28 | -1.07(-1.24–-0.91) |
| Venezuela | 2.45(1.68–3.28) | 145.34 | 0.51(0.07–0.96) | 130.62(89.06–175.89) | 135.25 | 0.82(0.32–1.32) |
| Viet Nam | 0.14(0.09–0.25) | 28.24 | -1.28(-1.41–-1.15) | 6.02(3.75–10.99) | 17.68 | -1.26(-1.41–-1.11) |
| Yemen | 0.37(0.21–0.6) | 191.75 | 0.06(-0.13–0.25) | 17.43(9.17–28.01) | 199.01 | 0.19(-0.02–0.39) |
| Zambia | 0.8(0.45–1.19) | 211.86 | 0.35(-0.23–0.92) | 31.44(17.37–46.25) | 224.27 | 0.54(-0.06–1.14) |
| Zimbabwe | 0.92(0.5–1.3) | 128.44 | 1.14(0.67–1.61) | 34.25(20.29–51.47) | 140.31 | 1.53(1–2.05) |

DALYs: disability adjusted life years; EAPC: estimated annual percentage change; ASR, age-standardized rate; CI, confidence interval; UI: uncertainty interval.

**Table S10**. Incident burden and trends of unintentional firearm injuries at the national level from 1990 to 2019.

|  | **1990** | | **2019** | | **1990-2019** | |
| --- | --- | --- | --- | --- | --- | --- |
| **Characteristics** | Number  ×10^2^ (95% UI) | ASR/100,000  (95% UI) | Number  ×10^2^ (95% UI) | ASR/100,000  (95% UI) | Percent (%) | EAPC  (95%CI) |
| Afghanistan | 34.14(21.94–51.22) | 25.64(17.26–36.49) | 113.4(72.01–172.19) | 25.22(17.08–35.84) | 232.12 | 0(-0.2–0.21) |
| Albania | 11.7(8.63–15.6) | 34.52(25.87–45.26) | 3.92(2.81–5.29) | 14.68(10.42–19.94) | -66.54 | -3.7(-4–-3.4) |
| Algeria | 82.77(53.87–120.59) | 30.49(21.07–41.88) | 135.16(90.84–190.32) | 32.11(21.74–44.84) | 63.29 | 0.3(0.1–0.5) |
| American Samoa | 0.34(0.23–0.49) | 64.43(46.29–88.55) | 0.21(0.14–0.31) | 38.01(24.89–55.69) | -37.69 | -2.29(-2.46–-2.13) |
| Andorra | 0.08(0.05–0.12) | 15.17(9.77–22.46) | 0.13(0.08–0.19) | 15.94(10.04–23.37) | 55.92 | 0.16(0.07–0.25) |
| Angola | 37.81(24.73–55.53) | 38.08(27.97–50.6) | 86.67(53.14–135.01) | 28.43(19.93–40.12) | 129.23 | -1.21(-1.32–-1.1) |
| Antigua and Barbuda | 1.24(0.93–1.61) | 198.1(152.13–254.74) | 2.07(1.57–2.7) | 229.75(174.49–298.6) | 67.51 | 0.47(0.17–0.77) |
| Argentina | 400.52(291.97–559.01) | 122.24(89.21–169.71) | 334.36(235.54–472.23) | 73.25(51.27–103.28) | -16.52 | -2.55(-2.88–-2.21) |
| Armenia | 12.32(9.11–16.44) | 37.45(28.24–49.27) | 5.82(4.18–8.04) | 19.44(13.7–27.17) | -52.78 | -2.26(-2.35–-2.17) |
| Australia | 28.04(18.68–40.46) | 16.98(11.28–24.67) | 35.81(23.62–51.54) | 15.59(9.95–23.05) | 27.72 | -0.45(-0.5–-0.39) |
| Austria | 12.54(8.47–17.58) | 16.28(10.81–22.9) | 15.11(10.27–21.84) | 17.34(11.21–25.38) | 20.55 | 0.24(0.13–0.35) |
| Azerbaijan | 12.04(8.01–17.53) | 16.12(10.88–22.84) | 16.73(11.25–24.32) | 16.55(10.87–23.94) | 39.05 | 0.23(0.06–0.39) |
| Bahamas | 1.76(1.18–2.59) | 66.17(45.64–95.01) | 2.84(1.95–3.99) | 78.85(53.43–112.28) | 61.38 | 0.73(0.48–0.98) |
| Bahrain | 1.82(1.18–2.71) | 33.74(22.52–48.62) | 5.18(3.36–7.5) | 37.37(25.07–52.82) | 183.95 | 0.6(0.4–0.79) |
| Bangladesh | 132.41(77.63–216.06) | 10.83(7.05–16.2) | 168.04(102.56–256.01) | 10.66(6.61–16.03) | 26.91 | -0.23(-0.41–-0.06) |
| Barbados | 2.75(1.98–3.8) | 105.46(76.33–145.1) | 2.51(1.83–3.35) | 90.8(64.49–127.26) | -8.59 | -0.69(-0.85–-0.52) |
| Belarus | 16.43(11.19–23.01) | 16.29(11.04–23.23) | 14.87(10.1–20.91) | 16.86(11.12–24.82) | -9.49 | 0.22(0.07–0.38) |
| Belgium | 15.51(10.53–22.07) | 15.73(10.43–22.7) | 17.58(11.84–25.22) | 15.66(10.16–23.4) | 13.39 | -0.56(-0.78–-0.33) |
| Belize | 2.82(2.03–3.8) | 153.52(116.76–200.15) | 6.92(5.11–9.2) | 166.77(125.26–217.99) | 145.81 | 0.11(-0.31–0.52) |
| Benin | 16.67(11–25.1) | 33.65(24.67–45.43) | 38.46(24.58–59.69) | 29.43(21.06–40.67) | 130.74 | -0.52(-0.61–-0.42) |
| Bermuda | 0.45(0.31–0.63) | 78.03(53.63–111.28) | 0.6(0.42–0.82) | 104.64(71.51–150.76) | 34.47 | 1.12(0.89–1.35) |
| Bhutan | 1.25(0.85–1.82) | 18.41(13–25.63) | 0.79(0.54–1.08) | 10.63(7.25–14.86) | -37.07 | -2.3(-2.49–-2.11) |
| Bolivia | 58.66(42.99–78.6) | 93.96(71–124.4) | 77.17(55.32–106.13) | 64.06(46.88–87.19) | 31.56 | -1.64(-1.79–-1.49) |
| Bosnia and Herzegovina | 9.36(6.35–13.24) | 21.02(14.49–29.48) | 5.5(3.75–7.73) | 16.78(11.11–23.94) | -41.27 | -0.86(-0.98–-0.73) |
| Botswana | 3.09(2.02–4.61) | 22.55(16–31.5) | 4.37(2.94–6.44) | 18.94(13.02–27.52) | 41.43 | -0.81(-0.92–-0.71) |
| Brazil | 994.07(667.87–1443.78) | 66.41(46.09–94.62) | 637(453.09–883.21) | 29.47(20.86–41.19) | -35.92 | -3.35(-3.79–-2.9) |
| Brunei Darussalam | 0.52(0.33–0.79) | 19.62(13.12–28.51) | 0.82(0.53–1.2) | 18.5(12.21–26.83) | 56.77 | -0.23(-0.29–-0.17) |
| Bulgaria | 17.28(12.17–23.81) | 20.61(14.55–28.64) | 13.06(9.15–18.07) | 19.08(13.01–26.69) | -24.43 | -0.16(-0.23–-0.09) |
| Burkina Faso | 29.88(19.24–45.39) | 31.38(23.14–42.26) | 64.2(40.74–99.42) | 28.16(20.16–39.11) | 114.88 | -0.41(-0.49–-0.32) |
| Burundi | 20.18(13.85–28.92) | 40.1(30.6–52.48) | 38.06(25.35–55.68) | 34.97(26.06–46.56) | 88.61 | -0.62(-0.72–-0.52) |
| Cabo Verde | 1.36(0.86–2.11) | 33.6(23.12–47.87) | 1.62(1.08–2.4) | 29.66(20.06–43.54) | 19.28 | -0.47(-0.62–-0.32) |
| Cambodia | 45.99(30.31–66.86) | 40.99(28.73–56.2) | 62.69(41.34–88.7) | 36.89(24.67–51.71) | 36.31 | -0.44(-0.59–-0.29) |
| Cameroon | 33.3(21.43–51.09) | 31.22(22.72–42.37) | 80.53(50.63–124.8) | 27.56(19–39.12) | 141.87 | -0.47(-0.56–-0.37) |
| Canada | 85.27(58.67–122.46) | 31.35(21.29–45.33) | 105.38(71.45–151.19) | 31.48(20.4–46.81) | 23.58 | -0.05(-0.22–0.13) |
| Central African Republic | 8.17(5.35–11.99) | 30.89(22.68–41.26) | 14.32(9.61–21.19) | 30.01(21.99–40.04) | 75.24 | -0.12(-0.19–-0.05) |
| Chad | 20.91(14.07–31.19) | 35.45(26.24–47.48) | 49.55(32.52–73.89) | 31.05(22.71–41.61) | 136.99 | -0.55(-0.6–-0.5) |
| Chile | 93.44(64.1–132.28) | 67.01(46.72–93.82) | 101.89(71.01–144.01) | 56.32(38.62–79.96) | 9.04 | -1.09(-1.74–-0.44) |
| China | 4207.82(2815.06–6104.31) | 35.92(24.01–51.52) | 3689.41(2494.79–5140.86) | 28.52(19.03–40.08) | -12.32 | -1.59(-2.02–-1.16) |
| Colombia | 221.06(155.68–309.6) | 66.72(49.1–88.47) | 214.44(152.33–296.23) | 44.91(31.37–62.8) | -2.99 | -1.78(-2.16–-1.4) |
| Comoros | 1.93(1.36–2.74) | 44.66(33.44–58.61) | 2.47(1.72–3.5) | 36.7(26.61–50.26) | 28.19 | -0.82(-0.93–-0.72) |
| Congo | 7.39(4.68–11.24) | 28.99(20.5–40.09) | 13.2(8.31–20.12) | 25.52(17.73–36.67) | 78.67 | -0.53(-0.62–-0.44) |
| Cook Islands | 0.07(0.04–0.11) | 32.31(20.02–49.47) | 0.05(0.03–0.08) | 33.2(19.78–50.61) | -20.53 | 0.12(-0.09–0.34) |
| Costa Rica | 14.13(9.35–20.39) | 45.61(31.77–63.99) | 24.23(16.62–34.07) | 51.84(34.67–73.97) | 71.48 | 0.62(0.46–0.78) |
| Croatia | 19.16(13.44–26.52) | 39.94(28.24–56.41) | 7.97(5.82–10.83) | 18.73(13.29–25.68) | -58.42 | -3.31(-3.77–-2.85) |
| Cuba | 74.77(52.31–104.64) | 68.81(48.07–96.18) | 133.75(97.51–179.87) | 124.79(87.85–175.08) | 78.89 | 2.64(2.37–2.9) |
| Cyprus | 1.72(1.16–2.43) | 22.47(15.18–31.84) | 2.24(1.5–3.27) | 17.33(11.3–25.35) | 29.78 | -1.26(-1.37–-1.15) |
| Czechia | 17.18(11.75–24.43) | 16.74(11.29–23.89) | 17.67(11.96–25.06) | 16.37(10.94–23.67) | 2.83 | -0.47(-0.66–-0.27) |
| Côte d'Ivoire | 39.39(25.39–59.95) | 32.17(23.44–43.56) | 74.45(48.42–112.91) | 28.82(20.37–40.39) | 89.01 | -0.47(-0.55–-0.38) |
| DPR Korea | 52.98(35.93–75.21) | 22.9(15.65–31.83) | 47.31(32.52–65.54) | 20.23(13.49–28.41) | -10.71 | -0.47(-0.55–-0.38) |
| DR Congo | 114.77(73.65–176.59) | 29.23(21.08–40.23) | 223.83(141.73–336.61) | 26.41(18.93–36.13) | 95.03 | -0.37(-0.46–-0.28) |
| Denmark | 8.34(5.62–11.94) | 16.43(10.8–23.68) | 9.7(6.46–13.8) | 17.03(10.99–25.18) | 16.25 | 0.13(0.07–0.2) |
| Djibouti | 1.78(1.22–2.6) | 41.43(30.78–55.06) | 3.71(2.55–5.34) | 33.65(24.52–46.24) | 108.27 | -0.86(-0.95–-0.76) |
| Dominica | 0.51(0.35–0.72) | 66.56(46.61–91.67) | 0.5(0.36–0.68) | 75.32(52.93–104.18) | -1.43 | 0.4(0.23–0.57) |
| Dominican Republic | 65.02(45.12–93.23) | 88.22(63.99–120.07) | 153.92(111.68–210.73) | 138.19(101.06–187.33) | 136.73 | 2.19(1.5–2.88) |
| Ecuador | 65.42(46.11–92.21) | 63.66(46.48–85.77) | 96.95(67.02–136.46) | 55.28(38.68–76.86) | 48.2 | -0.78(-1.01–-0.55) |
| Egypt | 203.02(132.88–296.58) | 32.81(22.63–46.11) | 283.24(187.48–410.12) | 27.77(18.69–39.68) | 39.52 | -0.66(-0.77–-0.54) |
| El Salvador | 21.51(14.12–31.92) | 39.88(27.61–56.07) | 29.45(19.94–42.89) | 46.46(31.27–66.24) | 36.9 | 0.6(0.35–0.85) |
| Equatorial Guinea | 1.52(1.01–2.23) | 34.81(25.75–46.55) | 3.85(2.36–5.9) | 27.68(18.8–39.92) | 153.96 | -0.89(-1.07–-0.72) |
| Eritrea | 11.26(7.71–15.98) | 45(33.44–59.13) | 20.94(14.37–30.17) | 35.94(26.58–48.19) | 86.06 | -0.89(-0.95–-0.83) |
| Estonia | 3.04(2.14–4.15) | 20.15(14.06–27.83) | 2.24(1.51–3.21) | 17.96(11.53–26.09) | -26.35 | -0.51(-0.7–-0.31) |
| Eswatini | 1.77(1.13–2.71) | 20.23(14.22–28.5) | 2.15(1.43–3.16) | 18.25(12.79–25.68) | 21.11 | -0.38(-0.47–-0.28) |
| Ethiopia | 240.98(167.94–341.5) | 60.78(45.5–79.35) | 386.99(255.45–565.43) | 40.68(29.91–54.43) | 60.59 | -1.64(-1.78–-1.5) |
| Fiji | 2.86(1.83–4.32) | 34.45(23.16–50.22) | 3(1.96–4.4) | 32.97(21.72–47.98) | 4.87 | -0.19(-0.42–0.03) |
| Finland | 8.2(5.6–11.74) | 17.12(11.3–24.82) | 9.37(6.47–13.16) | 17.38(11.48–25.29) | 14.17 | -0.35(-0.54–-0.16) |
| France | 132.72(90.51–187.33) | 22.91(15.52–32.74) | 143.34(99.98–202.32) | 21.01(14.12–30.04) | 8 | -0.4(-0.47–-0.33) |
| Gabon | 3.27(2.1–4.89) | 31.19(22.1–43.79) | 4.61(2.98–6.86) | 27.47(19.14–39.12) | 40.75 | -0.48(-0.58–-0.38) |
| Gambia | 3.53(2.23–5.44) | 34.07(24.73–46.41) | 6.95(4.46–10.61) | 30.73(21.88–43.12) | 97.11 | -0.37(-0.44–-0.29) |
| Georgia | 13.37(9.51–18.13) | 24.65(17.66–33.54) | 8.2(6.34–10.55) | 23.25(17.5–30.33) | -38.66 | 0.05(-0.1–0.2) |
| Germany | 130.67(87.99–186.16) | 16.55(11–24.02) | 141.21(95.11–202.56) | 16.98(10.96–25.02) | 8.07 | 0.11(0.06–0.17) |
| Ghana | 49.87(32.08–77.38) | 32.64(23.56–45.04) | 90.32(58.12–136.61) | 29.53(20.3–42) | 81.11 | -0.38(-0.48–-0.27) |
| Greece | 19.27(12.99–27.38) | 18.88(12.49–27.32) | 16.42(11.36–23.4) | 16.35(10.7–23.91) | -14.78 | -0.92(-1.11–-0.73) |
| Greenland | 0.82(0.58–1.14) | 136.61(97.29–186.38) | 0.28(0.2–0.39) | 52.33(36.81–74.79) | -65.34 | -3.64(-3.83–-3.45) |
| Grenada | 0.79(0.59–1.05) | 93.47(71.09–121.24) | 0.95(0.72–1.26) | 93.43(70.62–122.43) | 20.43 | -0.16(-0.28–-0.04) |
| Guam | 0.74(0.49–1.09) | 53.26(35.83–76.38) | 0.63(0.4–0.93) | 37.6(23.91–57.08) | -15.98 | -1.59(-1.75–-1.43) |
| Guatemala | 72.39(52.93–98.14) | 95.35(72.58–123.46) | 342.41(252.41–453.32) | 191.52(144.8–247.34) | 373.03 | 3.01(2.54–3.49) |
| Guinea | 22.82(15.26–33.31) | 35.86(26.02–48.1) | 37.09(24.78–55.28) | 30.64(22.48–40.97) | 62.52 | -0.6(-0.69–-0.5) |
| Guinea–Bissau | 3.56(2.38–5.24) | 36.68(27.35–48.67) | 5.25(3.47–7.95) | 29.21(21.28–40.16) | 47.47 | -0.91(-0.97–-0.85) |
| Guyana | 4.28(2.89–6.13) | 52.18(36.78–71.43) | 4.8(3.36–6.77) | 62.9(44.15–87.06) | 12.07 | 0.67(0.51–0.84) |
| Haiti | 183.87(134.14–249.48) | 308.27(224.47–410.96) | 260.14(183.43–352.68) | 212.95(152.83–285.34) | 41.48 | -1.52(-1.63–-1.4) |
| Honduras | 46.77(33.11–64.43) | 98.23(73.76–127.94) | 61.96(43.67–87.19) | 63.96(46.87–85.57) | 32.48 | -1.86(-1.99–-1.73) |
| Hungary | 17.53(12.02–24.81) | 17.08(11.38–24.28) | 16.6(11.46–23.68) | 17.03(11.42–24.6) | -5.31 | 0.09(-0.03–0.21) |
| Iceland | 0.4(0.26–0.57) | 15.5(10.08–22.25) | 0.56(0.37–0.8) | 16.48(10.57–24.29) | 41.52 | 0.21(0.17–0.26) |
| India | 1012.88(630.68–1526.43) | 11.41(7.52–16.49) | 1468.77(940.07–2173) | 10.74(6.92–15.71) | 45.01 | -0.29(-0.54–-0.05) |
| Indonesia | 1205.01(685.28–1946.86) | 60.05(35.49–92.49) | 1282.25(731.05–2026.54) | 50.87(29.29–80.21) | 6.41 | -0.68(-0.89–-0.47) |
| Iran | 197.2(125.57–297.96) | 30.56(20.74–43.13) | 222.63(149.7–313.76) | 27.18(18.3–38.61) | 12.89 | -0.38(-0.65–-0.12) |
| Iraq | 138.35(99.57–188.84) | 78.71(58.17–103.28) | 315.25(232.45–414.35) | 74.36(55.32–96.58) | 127.86 | -0.24(-0.3–-0.17) |
| Ireland | 6.17(4.17–8.88) | 17.25(11.7–24.89) | 8.41(5.69–11.99) | 17.54(11.43–25.24) | 36.44 | 0.06(-0.03–0.14) |
| Israel | 13.71(9.34–20.42) | 27.6(19–40.83) | 16.38(10.7–23.89) | 17.65(11.38–26.04) | 19.44 | -2.16(-2.43–-1.89) |
| Italy | 175.2(91.23–289.99) | 31.8(15.92–54.22) | 110.03(58.9–178.4) | 18.7(9.42–30.78) | -37.2 | -2.79(-3.31–-2.27) |
| Jamaica | 68.59(50.37–90.67) | 314.03(231.05–409.97) | 65.1(47.16–87.02) | 220.21(160.68–292.16) | -5.08 | -2.35(-2.74–-1.97) |
| Japan | 310.85(204.66–447.09) | 25.08(16.47–36.69) | 312.68(206.98–444.56) | 25.1(16.21–36.88) | 0.59 | 0.06(-0.11–0.23) |
| Jordan | 12.52(7.99–18.68) | 29.47(20.3–41.17) | 38.94(24.71–57.65) | 32.74(21.37–47.74) | 211.03 | 0.54(0.34–0.73) |
| Kazakhstan | 29.87(21.81–40.1) | 18.15(13.41–24.01) | 25.61(18.02–35.65) | 14.25(10–19.8) | -14.26 | -0.97(-1.08–-0.86) |
| Kenya | 87.99(57.15–131.13) | 42.42(31.17–57.81) | 166.08(107.58–243.59) | 35.65(25.22–50.13) | 88.75 | -0.73(-0.82–-0.63) |
| Kiribati | 0.23(0.15–0.35) | 27.18(18.19–39.56) | 0.38(0.24–0.57) | 29.43(19.21–42.06) | 64.47 | 0.38(0.17–0.58) |
| Kuwait | 6.54(4.19–9.59) | 35.49(23.75–50.81) | 17.2(11.21–24.69) | 39.52(26.1–57.09) | 163 | 0.45(0.34–0.55) |
| Kyrgyzstan | 6.96(4.45–10.37) | 15.41(10.26–22.16) | 9.6(6.18–14.15) | 14.71(9.71–21.42) | 38 | -0.03(-0.19–0.13) |
| Lao People's Democratic Republic | 17.3(11.82–24.53) | 39.03(27.79–52.16) | 24.56(16.36–35.14) | 33.02(22.23–46.85) | 41.95 | -0.79(-0.96–-0.61) |
| Latvia | 4.42(3.16–6.01) | 17.26(12.17–23.97) | 3.09(2.12–4.3) | 17.11(11.47–24.49) | -30.08 | 0(-0.16–0.17) |
| Lebanon | 12.88(8.49–18.59) | 36.32(24.65–50.68) | 18.58(12.57–26.23) | 36.06(24.22–51.06) | 44.26 | 0.03(-0.12–0.17) |
| Lesotho | 4.58(3.07–6.72) | 24.17(17.21–33.74) | 4.56(3.15–6.44) | 21.68(15.3–30.3) | -0.44 | -0.34(-0.37–-0.32) |
| Liberia | 6.68(4.52–9.74) | 34.35(25.32–45.91) | 12.38(8.05–18.7) | 27.68(19.67–38.5) | 85.38 | -0.85(-0.98–-0.72) |
| Libya | 13.97(8.92–20.45) | 29.75(20.29–41.13) | 17.42(11.64–24.38) | 27.62(18.29–38.92) | 24.66 | -0.16(-0.34–0.03) |
| Lithuania | 6.87(4.81–9.59) | 19.23(13.28–27.14) | 4.62(3.15–6.52) | 17.54(11.45–25.58) | -32.71 | -0.35(-0.5–-0.2) |
| Luxembourg | 0.65(0.44–0.93) | 17.35(11.46–25.18) | 1.06(0.72–1.54) | 17.45(11.39–25.66) | 62.52 | 0.02(-0.01–0.06) |
| Madagascar | 47.31(32.5–66.39) | 42.01(31.3–55.36) | 93.64(62.15–142.28) | 36.97(26.86–51.23) | 97.94 | -0.47(-0.56–-0.39) |
| Malawi | 29.5(19.25–45.1) | 32.19(23.56–43.98) | 52.76(32.88–80.94) | 29.55(20.97–41.43) | 78.83 | -0.4(-0.51–-0.3) |
| Malaysia | 77.23(49.3–113.88) | 40.76(26.97–58.85) | 120.83(80.01–177.79) | 39.36(25.68–57.59) | 56.46 | -0.13(-0.22–-0.03) |
| Maldives | 1.15(0.74–1.73) | 45.78(30.88–64.79) | 2.13(1.39–3.1) | 41.96(27.29–61) | 85.25 | -0.41(-0.58–-0.24) |
| Mali | 29.17(19.52–43.43) | 33.58(24.87–44.98) | 65.72(41.91–101.16) | 28.69(20.57–39.27) | 125.29 | -0.64(-0.76–-0.52) |
| Malta | 0.73(0.5–1.03) | 19.96(13.72–28.53) | 0.8(0.56–1.13) | 18.85(12.54–27.06) | 10.32 | -0.07(-0.16–0.01) |
| Marshall Islands | 0.25(0.16–0.38) | 46.86(33.35–64.29) | 0.21(0.14–0.31) | 36.2(24.28–51.42) | -16.14 | -1.11(-1.18–-1.04) |
| Mauritania | 7.25(4.73–11.16) | 33.44(24.3–46.02) | 11.35(7.24–17.51) | 28.12(19.46–39.94) | 56.46 | -0.68(-0.82–-0.54) |
| Mauritius | 4.4(2.79–6.63) | 39.52(25.55–58.59) | 4.82(3.1–6.97) | 41.68(26.35–61.82) | 9.49 | 0.2(0.09–0.31) |
| Mexico | 828.24(551.12–1229.15) | 97.05(68.4–137.04) | 667.2(450.14–964.11) | 53.77(36.22–76.8) | -19.44 | -1.7(-1.98–-1.41) |
| Micronesia | 0.55(0.36–0.81) | 45.52(32.43–63.03) | 0.34(0.23–0.5) | 33.11(22.4–47.46) | -37.66 | -1.41(-1.54–-1.28) |
| Monaco | 0.05(0.04–0.08) | 18.04(11.81–26.67) | 0.07(0.05–0.1) | 18.45(12.03–26.95) | 27.02 | 0.1(0.05–0.15) |
| Mongolia | 3.24(2.11–4.72) | 15.2(10.45–21.4) | 4.7(3.16–6.62) | 14.01(9.56–19.71) | 45.05 | -0.08(-0.44–0.29) |
| Montenegro | 1.09(0.73–1.56) | 17.51(11.67–24.9) | 1.05(0.71–1.5) | 17.04(11.26–24.66) | -3.66 | -0.06(-0.15–0.04) |
| Morocco | 134.21(92.83–185.12) | 51.26(36.68–68.48) | 149.01(104.67–205.02) | 41.87(29.51–57.32) | 11.03 | -0.82(-0.89–-0.75) |
| Mozambique | 42.09(28.35–61.71) | 35.52(26.49–46.8) | 84.89(54.77–130.12) | 29.67(21.55–41) | 101.69 | -0.64(-0.74–-0.54) |
| Myanmar | 230.13(162.14–308.16) | 53.26(38.06–70.37) | 209.55(143.63–292.48) | 38.32(26.12–53.47) | -8.94 | -1.42(-1.57–-1.26) |
| Namibia | 3.29(2.19–4.77) | 22.52(15.93–31.28) | 4.91(3.19–7.52) | 19.51(13.27–28.55) | 49.45 | -0.68(-0.77–-0.59) |
| Nauru | 0.05(0.03–0.08) | 40.7(27.22–58.59) | 0.04(0.02–0.06) | 34.09(22.31–51.51) | -19.51 | -0.75(-0.81–-0.69) |
| Nepal | 25.37(15.26–39.86) | 12.84(8.44–18.86) | 33.25(20.57–51.3) | 11.24(7.27–16.66) | 31.07 | -0.54(-0.66–-0.41) |
| Netherlands | 30.23(20.53–43.66) | 20.31(13.32–29.67) | 36.1(23.81–52.98) | 21.47(13.58–31.6) | 19.44 | -0.37(-0.72–-0.02) |
| New Zealand | 6.83(4.05–10.75) | 20.16(12.06–31.68) | 5.15(3.58–7.31) | 12.22(8.38–17.19) | -24.57 | -1.57(-1.96–-1.18) |
| Nicaragua | 26.95(18.36–39.39) | 67.95(49.85–90.96) | 31.45(20.98–45.24) | 48.53(33.32–68.6) | 16.72 | -1.45(-1.57–-1.32) |
| Niger | 25.78(16.58–39.02) | 32.62(23.9–43.85) | 66.81(40.9–106.46) | 27.84(19.86–38.27) | 159.1 | -0.66(-0.79–-0.54) |
| Nigeria | 345.97(228.52–524.64) | 39.07(28.2–53.74) | 612.55(385.06–949.86) | 28.49(19.83–40.41) | 77.05 | -1.35(-1.52–-1.18) |
| Niue | 0.01(0.01–0.01) | 37.83(24.88–55.6) | 0.01(0–0.01) | 33.41(21.91–49.66) | -45.6 | -0.6(-0.74–-0.45) |
| North Macedonia | 3.71(2.47–5.3) | 18.48(12.38–26.28) | 3.66(2.48–5.24) | 17.05(11.45–24.47) | -1.45 | -0.38(-0.44–-0.32) |
| Northern Mariana Islands | 0.29(0.2–0.41) | 68.84(49.26–93.61) | 0.15(0.1–0.21) | 41.97(27.76–62.16) | -48.36 | -2.02(-2.12–-1.91) |
| Norway | 9.19(6.26–12.99) | 21.79(14.46–31.51) | 11.27(7.48–16.16) | 21.07(13.75–30.99) | 22.64 | -0.16(-0.29–-0.02) |
| Oman | 7.79(5.03–11.1) | 36.65(25.12–50.49) | 18.5(12.06–26.49) | 38.31(25.73–53.38) | 137.45 | 0.26(0.04–0.49) |
| Pakistan | 132.41(82.03–207.08) | 11.28(7.56–16.13) | 226.65(138.16–349.61) | 10.07(6.59–14.53) | 71.18 | -0.48(-0.65–-0.31) |
| Palau | 0.07(0.05–0.09) | 41.86(29.07–58.88) | 0.06(0.04–0.08) | 36.7(24.92–52.9) | -15.65 | -0.53(-0.65–-0.42) |
| Palestine | 11.65(7.87–16.71) | 52.76(37.36–72.44) | 19(12.38–28.1) | 35.36(24.18–50.89) | 62.99 | -1.55(-1.87–-1.22) |
| Panama | 10.73(7.3–15.51) | 44.3(31.2–61.19) | 21.08(14.42–29.38) | 50.54(34.56–69.97) | 96.44 | 0.42(0.23–0.61) |
| Papua New Guinea | 17.37(11.08–26.12) | 36.17(24.49–51.62) | 39.78(25.96–58.62) | 35.44(24.15–49.75) | 129.06 | -0.11(-0.17–-0.05) |
| Paraguay | 18.54(13.11–26.49) | 45.05(33.17–60.86) | 23.45(16.25–33.53) | 33.24(23.55–46.78) | 26.53 | -1.3(-1.44–-1.17) |
| Peru | 225.5(164.25–302.44) | 105.09(77.93–138.84) | 284.19(206.55–389.97) | 82.47(59.91–113.01) | 26.03 | -0.73(-0.83–-0.63) |
| Philippines | 188.61(123.09–272.04) | 28.04(19–39.39) | 560.94(363.4–812.55) | 48.64(32.22–69.46) | 197.41 | 2.31(2.08–2.54) |
| Poland | 74.05(49.99–104.27) | 19.59(13.08–27.92) | 77.31(52.72–111.3) | 19.87(13.2–28.62) | 4.4 | 0.09(-0.1–0.29) |
| Portugal | 22.01(15.43–30.63) | 21.93(15.06–30.85) | 18.02(11.91–26.04) | 17.21(10.98–25.19) | -18.12 | -1.19(-1.33–-1.05) |
| Puerto Rico | 28.45(19.7–40.71) | 78.56(54.31–112.48) | 29.42(20.9–40.11) | 89.83(60.66–127.9) | 3.41 | 0.61(0.49–0.73) |
| Qatar | 1.75(1.17–2.51) | 38.05(26.25–53.86) | 11.02(7.41–15.76) | 39.08(26.35–55.29) | 527.99 | 0.26(-0.03–0.54) |
| Republic of Korea | 86.01(56.92–125) | 18.77(12.58–26.85) | 114.41(77.58–166.27) | 22.94(14.76–34.27) | 33.02 | 0.77(0.67–0.87) |
| Republic of Moldova | 8.48(5.86–11.69) | 19.23(13.42–26.47) | 5.58(3.77–7.92) | 15.93(10.48–23.31) | -34.17 | -0.82(-1.07–-0.56) |
| Romania | 39.36(27.68–54.92) | 16.99(11.86–23.63) | 33.63(23.07–47.64) | 17.4(11.65–24.97) | -14.57 | 0.09(-0.03–0.2) |
| Russian Federation | 348.48(241.12–487.63) | 23.96(16.35–33.91) | 260.73(178.08–367.13) | 18.57(12.34–26.99) | -25.18 | -0.98(-1.1–-0.86) |
| Rwanda | 25.86(17.19–38.05) | 38.8(28.55–51.69) | 34.72(22.33–52) | 29.01(20.34–40.18) | 34.28 | -1.28(-1.44–-1.12) |
| Saint Kitts and Nevis | 1.06(0.78–1.41) | 266.34(202.36–350.49) | 1.32(1–1.72) | 217.41(165.47–284.88) | 24.73 | -0.79(-0.98–-0.61) |
| Saint Lucia | 1.21(0.87–1.68) | 86.53(64.75–114.49) | 1.67(1.24–2.17) | 98.83(72.21–132.22) | 38.22 | 0.53(0.41–0.64) |
| Saint Vincent and the Grenadines | 1.13(0.82–1.53) | 101.26(76.29–132.32) | 1.09(0.82–1.43) | 98.7(74.56–131.16) | -3.45 | -0.17(-0.31–-0.04) |
| Samoa | 0.74(0.47–1.1) | 41.1(28.25–58.13) | 0.73(0.47–1.09) | 33.3(22.03–48.18) | -1.2 | -0.96(-1.1–-0.82) |
| San Marino | 0.04(0.03–0.05) | 16.13(10.61–23.44) | 0.06(0.04–0.08) | 17.17(11.32–25.17) | 50.87 | 0.27(0.22–0.32) |
| Sao Tome and Principe | 0.42(0.27–0.63) | 31.32(22.38–43.06) | 0.57(0.37–0.86) | 28.05(19.09–40.13) | 36.85 | -0.44(-0.56–-0.33) |
| Saudi Arabia | 95.99(67.72–131.77) | 58.66(42.62–77.34) | 145.29(102.9–195.01) | 39.91(28.4–53.72) | 51.35 | -1.37(-1.41–-1.33) |
| Senegal | 26.5(17.08–39.76) | 34.11(24.67–45.55) | 44.32(29.02–67.13) | 29.83(21.18–41.68) | 67.28 | -0.53(-0.63–-0.42) |
| Serbia | 19.31(13.3–26.87) | 20.7(14.06–29.02) | 16.07(11.13–22.8) | 18.18(12.17–26.23) | -16.76 | -0.6(-0.68–-0.52) |
| Seychelles | 0.37(0.25–0.55) | 49.86(33.49–71.73) | 0.43(0.28–0.62) | 44.17(28.2–65.46) | 15.7 | -0.45(-0.56–-0.35) |
| Sierra Leone | 12.23(8.24–17.81) | 32.95(24.17–44.54) | 21.89(14.61–32.36) | 27.79(20.19–38.19) | 79.02 | -0.64(-0.69–-0.59) |
| Singapore | 6.55(4.25–9.6) | 20.87(13.72–30.48) | 11.56(7.52–17.05) | 21.1(13.48–31.03) | 76.36 | 0.02(-0.08–0.12) |
| Slovakia | 12.1(8.57–16.52) | 22.76(16.05–31.1) | 10.68(7.44–14.92) | 19.26(13.06–27.11) | -11.71 | -0.74(-0.82–-0.65) |
| Slovenia | 3.27(2.27–4.46) | 16.72(11.44–22.93) | 3.6(2.46–5.19) | 16.97(11.27–24.41) | 10.28 | -0.51(-0.75–-0.26) |
| Solomon Islands | 1.9(1.26–2.81) | 44.96(31.75–62.54) | 2.95(1.97–4.28) | 38.44(26.45–54.76) | 55.01 | -0.63(-0.66–-0.6) |
| Somalia | 30.82(21.44–42.61) | 53.59(40.38–69.88) | 81.59(57.16–114.71) | 51.17(38.55–66.74) | 164.72 | -0.2(-0.26–-0.14) |
| South Africa | 89.83(59.47–134.09) | 23.6(16.1–34.07) | 114.07(75.14–170.37) | 21.12(13.9–31.47) | 26.99 | -0.41(-0.54–-0.29) |
| South Sudan | 22.9(15.64–32.89) | 44.96(33.18–59.28) | 32.52(21.94–47.95) | 39.07(28.61–52.38) | 41.98 | -0.56(-0.65–-0.46) |
| Spain | 89.17(61.2–126.32) | 23.03(15.53–33.18) | 77.96(52.32–113.85) | 17.3(11.25–25.21) | -12.57 | -1.19(-1.45–-0.93) |
| Sri Lanka | 131.42(94.03–177.57) | 74.58(55.13–98.32) | 91.97(64.4–127.54) | 44.57(30.7–62.33) | -30.02 | -2.3(-2.52–-2.07) |
| Sudan | 64.65(43.49–95) | 29.92(21.36–41.84) | 113.53(75.69–165.33) | 26.23(18.24–36.62) | 75.61 | -0.51(-0.66–-0.36) |
| Suriname | 2.69(1.89–3.76) | 66.58(48.32–90.26) | 4.21(3.05–5.75) | 74.78(53.91–103.45) | 56.72 | 0.43(0.33–0.53) |
| Sweden | 17.88(11.87–25.17) | 21.03(13.91–30.61) | 21.9(14.41–30.97) | 21.47(14.05–31.5) | 22.47 | 0.06(-0.02–0.15) |
| Switzerland | 13.87(9.77–19.59) | 20.16(13.97–28.66) | 13.55(9.06–19.65) | 15.6(10.13–22.66) | -2.31 | -1.53(-2.04–-1.03) |
| Syrian Arab Republic | 47.64(31.78–68.92) | 32.33(23.05–43.85) | 43.77(28.28–64.11) | 30.93(20.13–44.76) | -8.13 | -0.14(-0.29–0.01) |
| Taiwan  (Province of China) | 38.39(25.62–55.01) | 19.43(13.01–27.47) | 40.87(27.35–58.48) | 20.33(12.98–30.25) | 6.46 | 0.17(0.02–0.33) |
| Tajikistan | 7.93(4.96–12.41) | 14.76(9.64–21.67) | 13.15(8.33–19.96) | 14.03(9.26–20.55) | 65.78 | -0.16(-0.34–0.01) |
| Thailand | 305.13(214.07–427.88) | 52.91(37.54–73.03) | 248.66(171.74–350.75) | 39.88(27.08–56.99) | -18.51 | -1.31(-1.43–-1.18) |
| Timor–Leste | 3.68(2.48–5.27) | 43.16(30.31–59.31) | 5.38(3.47–7.81) | 38.09(25.41–54.01) | 46.25 | -0.49(-0.64–-0.35) |
| Togo | 12.19(7.86–18.74) | 32.4(23.88–44.57) | 22.52(14.48–34.95) | 28.89(20.29–40.66) | 84.65 | -0.45(-0.54–-0.36) |
| Tokelau | 0.01(0.01–0.01) | 41.15(27.67–59.23) | 0.01(0–0.01) | 33.79(21.63–50.69) | -38.77 | -0.94(-1.09–-0.79) |
| Tonga | 0.4(0.24–0.62) | 36.37(23.41–53.66) | 0.39(0.24–0.59) | 35.23(22.39–53.02) | -3.38 | -0.15(-0.31–0.01) |
| Trinidad and Tobago | 8.32(5.64–12.01) | 67.22(46.5–94.82) | 10.59(7.26–14.59) | 80.58(54.32–114.21) | 27.31 | 0.71(0.39–1.02) |
| Tunisia | 23.25(16.28–31.87) | 27.25(19.49–36.23) | 30.13(21.44–40.35) | 26.61(18.84–36.03) | 29.62 | -0.08(-0.31–0.15) |
| Turkey | 323.23(227.09–447.19) | 54.15(39.22–73.31) | 354.92(256.37–482.94) | 44.17(31.51–61.04) | 9.8 | -0.76(-1–-0.51) |
| Turkmenistan | 6.26(4.19–9.16) | 16.35(11.44–23.02) | 9.88(6.94–13.88) | 19.35(13.82–26.88) | 57.9 | 0.84(0.73–0.94) |
| Tuvalu | 0.04(0.03–0.07) | 43.94(29.89–61.78) | 0.04(0.03–0.06) | 34.46(22.15–50.55) | -9.64 | -1.06(-1.21–-0.92) |
| Uganda | 61.35(38.7–94.87) | 35.2(25.55–48.3) | 123.38(76.06–194.71) | 29.19(20.47–41.41) | 101.12 | -0.79(-0.93–-0.65) |
| Ukraine | 99.35(68.67–140.49) | 19.74(13.38–28.36) | 76.03(51.84–107.26) | 18.53(12.38–26.93) | -23.47 | -0.19(-0.38–0) |
| United Arab Emirates | 6.56(4.33–9.45) | 32.21(21.99–44.62) | 26.59(16.75–38.67) | 31.03(20.61–44.07) | 305.07 | -0.06(-0.24–0.11) |
| United Kingdom | 103.92(69.07–148.08) | 18.33(11.97–26.48) | 124.13(83.26–179.3) | 18.67(11.87–27.53) | 19.44 | 0.12(0.01–0.22) |
| United Republic of Tanzania | 91.18(59.17–138.04) | 35.66(26.25–48.45) | 181.82(117.59–279.47) | 32.22(22.91–45.01) | 99.4 | -0.42(-0.53–-0.31) |
| United States of America | 1648.13(1121.5–2376.48) | 65.76(45.06–95.14) | 1068.1(715.47–1522.82) | 33.82(22.29–49.54) | -35.19 | -3.03(-3.93–-2.13) |
| United States Virgin Islands | 0.89(0.62–1.24) | 86.1(61.58–119.77) | 0.95(0.69–1.27) | 99.81(70.93–137.08) | 6.96 | 0.54(0.47–0.61) |
| Uruguay | 131.31(98.74–173.16) | 420.15(312.66–555.45) | 58.37(43.22–78.92) | 165.6(120.52–229.17) | -55.55 | -3.78(-4.01–-3.55) |
| Uzbekistan | 34.69(21.99–53.34) | 16.67(11.03–24.13) | 58.36(37.55–86.08) | 17.38(11.45–25.24) | 68.24 | 0.25(0.05–0.45) |
| Vanuatu | 0.82(0.54–1.2) | 47.4(34.06–65.25) | 1.15(0.74–1.73) | 36.1(24.77–51.24) | 41.49 | -1.14(-1.23–-1.05) |
| Venezuela | 268.08(191.43–371.67) | 130.86(96.44–175.47) | 400.3(286.67–547.95) | 146.6(103.98–202.28) | 49.32 | 0.74(0.45–1.03) |
| Viet Nam | 421.45(294.7–582.49) | 59.85(43.62–79.66) | 366(249.97–504.98) | 39.56(27.11–55.35) | -13.16 | -1.82(-1.99–-1.65) |
| Yemen | 45.35(29.21–66.94) | 30.09(20.91–41.53) | 87.25(56.52–127.95) | 25.57(17.45–35.59) | 92.41 | -0.51(-0.66–-0.37) |
| Zambia | 26.84(18.29–39.09) | 38.25(28.54–50.32) | 51.04(32.7–77.74) | 29.87(21.77–41.11) | 90.14 | -1.04(-1.12–-0.96) |
| Zimbabwe | 20.11(11.74–32.82) | 17.07(11.37–25.22) | 26.49(16.07–42.66) | 16.31(10.7–24.87) | 31.75 | -0.16(-0.27–-0.05) |

EAPC: estimated annual percentage change; ASR, age-standardized rate; CI, confidence interval; UI: uncertainty interval.

**Table S11**. Global trends in death and DALYs due to unintentional firearm injuries in sexes, SDI areas, and regions, 1990-2019.

| **Characteristics** | **Death** | | | | **DALYs** | | | |  |
| --- | --- | --- | --- | --- | --- | --- | --- | --- | --- |
|  | 2019 | | 1990-2019 | | 2019 | | 1990-2019 | | |
|  | Number  ×10^3^ (95% UI) | ASR/100,000  (95% UI) | % | EAPC  (95%CI) | Number  ×10^3^ (95% UI) | ASR/100,000  (95% UI) | % | EAPC  (95%CI) | |
| **Overall** | 20.07  (14.71–25.08) | 0.26  (0.19–0.32) | -26.54 | -2.55(-2.66–-2.45) | 1363.71  (1051.13–1660.17) | 17.64  (13.47–21.53) | -25.35 | -2.31  (-2.44–-2.17) | |
| **Sex** |  |  |  |  |  |  |  |  | |
| Male | 15.2  (10.59–19.61) | 0.39  (0.27–0.5) | -29.29 | -2.72  (-2.83–-2.62) | 982.9  (736.83–1246.57) | 25.06  (18.73–31.86) | -28.46 | -2.49  (-2.61–-2.36) | |
| Female | 4.86  (3.53–6.37) | 0.13  (0.09–0.17) | -16.4 | -2.01  (-2.12–-1.89) | 380.81  (285.49–479.86) | 10.15  (7.39–13.06) | -15.93 | -1.78  (-1.94–-1.62) | |
| **SDI** |  |  |  |  |  |  |  |  | |
| Low | 6.83  (4.69–9.78) | 0.85  (0.6–1.17) | 25.59 | -1.77  (-1.9–-1.64) | 413.82  (284.29–589.84) | 38.38  (27.61–54.04) | 21.98 | -1.76  (-1.85–-1.67) | |
| Low-middle | 4.8  (3.41–6.08) | 0.29  (0.21–0.36) | -11.44 | -2.22  (-2.28–-2.15) | 318.96  (244.33–393.59) | 17.93  (13.76–22.1) | -12.8 | -1.94  (-2.02–-1.87) | |
| Middle | 4.75  (3.39–6.11) | 0.19  (0.14–0.25) | -49.58 | -3.89  (-4.15–-3.63) | 346.95  (272.38–425.55) | 14.06  (11.01–17.33) | -45.42 | -3.4  (-3.67–-3.13) | |
| High-middle | 2.3  (1.67–2.64) | 0.15  (0.11–0.18) | -47.95 | -3.61  (-3.83–-3.4) | 172.31  (135.62–200.45) | 11.64  (9.06–13.4) | -42.6 | -3.2  (-3.43–-2.96) | |
| High | 1.35  (1.07–1.71) | 0.13  (0.1–0.16) | -48 | -3.25  (-3.49–-3.01) | 108.83  (89.81–133.27) | 10.29  (8.62–12.54) | -40.77 | -2.88  (-3.19–-2.57) | |
| **Regions** |  |  |  |  |  |  |  |  | |
| East Asia | 0.36  (0.25–0.62) | 0.02  (0.02–0.04) | -92.04 | -10.76  (-11.63–-9.88) | 60.5  (44.33–87.18) | 3.64  (2.68–5.17) | -80.17 | -7.82  (-8.75–-6.89) | |
| South Asia | 1.25  (0.66–1.7) | 0.08  (0.04–0.1) | -30.65 | -3.8  (-4.01–-3.6) | 105.85  (69.92–134.41) | 5.99  (3.96–7.63) | -18.78 | -2.81  (-2.98–-2.64) | |
| Southeast Asia | 0.79  (0.4–1.14) | 0.12  (0.06–0.17) | -62.58 | -5.2  (-5.51–-4.9) | 83.81  (57.34–107.71) | 12.4  (8.3–16.23) | -49.11 | -3.97  (-4.2–-3.74) | |
| Central Asia | 0.18  (0.12–0.22) | 0.2  (0.14–0.24) | -18.61 | -2.33  (-2.61–-2.05) | 11.68  (8.57–13.6) | 12.32  (9.14–14.3) | -17.32 | -2.31  (-2.53–-2.09) | |
| High-income Asia Pacific | 0.04  (0.03–0.05) | 0.01  (0.01–0.02) | -53.82 | -4.13  (-4.44–-3.81) | 10.46  (7.51–14.43) | 4.13  (3.03–5.61) | -0.69 | -1.02  (-1.31–-0.74) | |
| Oceania | 0.03  (0.02–0.08) | 0.25  (0.14–0.53) | 49.32 | -1.23  (-1.38–-1.07) | 3.17  (1.79–7.7) | 21.05  (13.35–44.07) | 62.01 | -0.74  (-0.89–-0.58) | |
| Australasia | 0.01  (0.01–0.02) | 0.04  (0.03–0.07) | -67.53 | -5.83  (-6.45–-5.21) | 1.49  (1.18–1.93) | 4.75  (3.82–6.27) | -45.92 | -3.91  (-4.27–-3.54) | |
| Eastern Europe | 0.4  (0.29–0.51) | 0.18  (0.14–0.23) | -51.53 | -3.53  (-4.02–-3.03) | 26.92  (21.81–33.04) | 12.54  (10.33–15.47) | -52.27 | -3.44  (-3.85–-3.03) | |
| Western Europe | 0.33  (0.22–0.37) | 0.06  (0.04–0.07) | -48.14 | -3.25  (-3.37–-3.13) | 29.2  (23.14–35.71) | 5.73  (4.58–6.9) | -36.69 | -2.51  (-2.66–-2.36) | |
| Central Europe | 0.09  (0.06–0.11) | 0.07  (0.05–0.09) | -64.68 | -4.32  (-4.6–-4.03) | 8.26  (6.5–10.21) | 6.46  (5.14–7.89) | -54.53 | -3.42  (-3.72–-3.13) | |
| High-income North America | 0.69  (0.59–0.87) | 0.18  (0.16–0.23) | -59.16 | -4.38  (-4.73–-4.03) | 49.89  (42.3–61.2) | 13.62  (11.88–16.22) | -57.54 | -4.33  (-4.8–-3.86) | |
| Andean Latin America | 0.47  (0.32–0.66) | 0.73  (0.5–1.01) | -20.26 | -2.84  (-3.04–-2.64) | 32.61  (24.2–42.43) | 50.24  (37.45–65.31) | -18.53 | -2.62  (-2.78–-2.45) | |
| Central Latin America | 2.21  (1.74–2.68) | 0.85  (0.67–1.03) | -17.44 | -1.64  (-2.28–-1) | 144.39  (119.06–170.25) | 55.28  (45.71–65.16) | -16.81 | -1.43  (-2–-0.86) | |
| Caribbean | 0.97  (0.52–1.87) | 2.02  (1.08–3.87) | 37.95 | 0.22  (0.04–0.4) | 64.28  (37.87–114.47) | 136.46  (79.4–242.35) | 33.57 | 0.31  (0.15–0.47) | |
| Tropical Latin America | 0.69  (0.49–0.77) | 0.3  (0.21–0.33) | -31.23 | -2.95  (-3.31–-2.58) | 44.88  (34.28–50.17) | 19.49  (14.98–21.7) | -36.17 | -3.01  (-3.34–-2.69) | |
| Southern Latin America | 0.51  (0.46–0.57) | 0.72  (0.65–0.8) | -29.87 | -3.24  (-3.66–-2.82) | 33.86  (29.78–38.21) | 48.69  (43.05–54.55) | -30.42 | -3.15  (-3.52–-2.78) | |
| Eastern sub- Saharan Africa | 3.35  (2.26–4.45) | 1.41  (1–1.79) | 21 | -1.76  (-1.91–-1.62) | 186.84  (124.74–259.85) | 52.79  (37.4–69.19) | 14.7 | -1.94  (-2.08–-1.81) | |
| Southern sub- Saharan Africa | 0.37  (0.24–0.54) | 0.47  (0.3–0.68) | 1.99 | -2.08  (-2.56–-1.6) | 20.83  (14.14–29.95) | 25.21  (17.02–35.68) | -0.5 | -2.08  (-2.55–-1.6) | |
| Western sub- Saharan Africa | 2.83  (1.66–3.97) | 0.94  (0.59–1.25) | 25.68 | -1.84  (-1.96–-1.71) | 170.15  (96.88–244.84) | 38.39  (23.85–52.9) | 22.37 | -2.01  (-2.13–-1.88) | |
| North Africa  and Middle East | 3.66  (2.38–5.17) | 0.6  (0.39–0.85) | 6.6 | -2.21  (-2.33–-2.09) | 227.64  (157.84–310.14) | 36.11  (25.25–49.25) | 3.58 | -2.06  (-2.18–-1.95) | |
| Central sub- Saharan Africa | 0.8  (0.47–1.55) | 1.03  (0.64–1.83) | 40.51 | -1.31  (-1.58–-1.05) | 47  (27.86–89.81) | 41.68  (26.57–75.8) | 31.15 | -1.51  (-1.75–-1.28) | |

DALYs: disability adjusted life years; EAPC: estimated annual percentage change; ASR, age-standardized rate; CI, confidence interval; UI: uncertainty interval; SDI: socio-demographic index.

**Table S12**. Burden and trends in death and DALYs due to unintentional firearm injuries at the national level from 1990 to 2019.

|  | **Death** | | | **DALYs** | | |
| --- | --- | --- | --- | --- | --- | --- |
| **Characteristics** | ASR/100,000  (95% UI) | Percent  (%) | EAPC  (95%CI) | ASR/100,000  (95% UI) | Percent (%) | EAPC  (95%CI) |
| Afghanistan | 0.29(0.06–0.65) | 83.96 | -2.38(-3.02–-1.72) | 19.76(8.24–37.4) | 113 | -1.96(-2.48–-1.43) |
| Albania | 0.13(0.07–0.34) | -87.07 | -8.31(-9.3–-7.32) | 9.82(6.39–21.62) | -85.62 | -7.73(-8.61–-6.85) |
| Algeria | 0.12(0.06–0.3) | -22.38 | -2.69(-2.75–-2.64) | 11.28(7.25–20.87) | -5.47 | -1.93(-2.01–-1.84) |
| American Samoa | 0.18(0.11–0.41) | -73.35 | -7.25(-7.74–-6.75) | 11.1(7.57–22.36) | -70.29 | -6.16(-6.6–-5.73) |
| Andorra | 0.02(0.01–0.04) | 1.52 | -2.06(-2.18–-1.93) | 3.57(2.54–5.02) | 58.15 | -0.5(-0.59–-0.42) |
| Angola | 0.87(0.58–1.23) | 3.72 | -3.08(-3.31–-2.84) | 35.51(23.99–53.28) | 0.57 | -3.37(-3.58–-3.16) |
| Antigua and Barbuda | 3.85(3.14–4.64) | 47.09 | -0.48(-1.06–0.1) | 226.82(186.52–271.75) | 45.91 | -0.48(-1.06–0.1) |
| Argentina | 0.83(0.75–0.94) | -8.93 | -2.52(-3.17–-1.86) | 54.48(48.4–61.57) | -15.24 | -2.63(-3.2–-2.06) |
| Armenia | 0.44(0.36–0.53) | -55.07 | -3.97(-4.47–-3.48) | 20.95(17.01–25.25) | -64.28 | -4.26(-4.8–-3.72) |
| Australia | 0.04(0.03–0.06) | -67.56 | -6.11(-6.91–-5.31) | 4.66(3.71–6.23) | -42.58 | -3.92(-4.41–-3.44) |
| Austria | 0.03(0.02–0.04) | -47.95 | -2.91(-3.61–-2.2) | 4.54(3.56–5.62) | -16.71 | -1.41(-1.84–-0.98) |
| Azerbaijan | 0.15(0.07–0.25) | -11.25 | -2.91(-3.34–-2.47) | 10.7(6.57–16.1) | -8.75 | -2.66(-3–-2.33) |
| Bahamas | 0.1(0.07–0.14) | -4.71 | -1.79(-2.33–-1.24) | 17.2(13.11–21.97) | 43.02 | -0.46(-0.75–-0.17) |
| Bahrain | 0.12(0.05–0.19) | 162.86 | 0.09(-0.49–0.67) | 11.83(7.46–15.93) | 180.04 | 0.1(-0.31–0.5) |
| Bangladesh | 0.08(0.03–0.14) | -28.67 | -3.57(-3.99–-3.15) | 5.93(3.65–9.52) | -21.62 | -2.59(-3–-2.18) |
| Barbados | 0.67(0.53–0.84) | 8.13 | -1.18(-1.87–-0.48) | 47.04(38.69–57.14) | 0.52 | -1.21(-1.78–-0.64) |
| Belarus | 0.09(0.06–0.15) | -49.33 | -2.53(-3.17–-1.89) | 7.03(5.06–10.43) | -42.91 | -2.08(-2.51–-1.66) |
| Belgium | 0.05(0.03–0.06) | -44.53 | -3.05(-3.29–-2.81) | 4.88(3.91–5.87) | -23.94 | -2.09(-2.32–-1.86) |
| Belize | 3.02(2.54–3.55) | 198.55 | -0.2(-1.14–0.75) | 179.5(152.66–208.4) | 171.51 | -0.39(-1.25–0.47) |
| Benin | 1.07(0.63–1.59) | 42.09 | -1.63(-1.71–-1.55) | 45(24.67–79.27) | 38.74 | -1.64(-1.71–-1.57) |
| Bermuda | 0.15(0.11–0.2) | -43.89 | -1.86(-2.4–-1.32) | 16.61(12.7–21.73) | -12.02 | -0.68(-1–-0.37) |
| Bhutan | 0.27(0.09–0.83) | -74.8 | -5.33(-5.58–-5.07) | 17.08(7.05–47.39) | -77.22 | -5.38(-5.68–-5.08) |
| Bolivia | 0.87(0.55–1.32) | -22.13 | -3.3(-3.37–-3.22) | 55.74(38.92–78.53) | -22.79 | -3.18(-3.26–-3.11) |
| Bosnia and Herzegovina | 0.06(0.03–0.09) | -71.87 | -4.99(-5.5–-4.48) | 5.31(3.96–7.07) | -64.98 | -3.66(-4.08–-3.24) |
| Botswana | 0.52(0.18–1.26) | 14.08 | -3.01(-3.46–-2.55) | 26.06(9.84–60.39) | 11.1 | -2.95(-3.38–-2.52) |
| Brazil | 0.3(0.21–0.33) | -31.73 | -2.94(-3.31–-2.57) | 19.31(14.51–21.55) | -36.83 | -3.02(-3.35–-2.68) |
| Brunei Darussalam | 0.02(0.01–0.02) | -18.82 | -2.56(-2.77–-2.34) | 3.95(2.96–5.1) | 47.24 | -1.04(-1.15–-0.92) |
| Bulgaria | 0.22(0.13–0.3) | -42.32 | -1.17(-1.5–-0.84) | 14.52(10.3–19.01) | -45.25 | -1.23(-1.5–-0.95) |
| Burkina Faso | 1.22(0.8–1.72) | 60.61 | -1.21(-1.37–-1.05) | 50.01(31.63–81.01) | 62.32 | -1.12(-1.37–-0.86) |
| Burundi | 1.86(0.94–2.92) | 44.66 | -1.01(-1.13–-0.89) | 73.79(39.95–120.26) | 38.68 | -1.16(-1.25–-1.07) |
| Cabo Verde | 0.39(0.24–0.56) | -18.66 | -2.84(-3.07–-2.6) | 16.38(10.28–25.9) | -36.08 | -3.32(-3.53–-3.1) |
| Cambodia | 0.14(0.08–0.26) | -55.7 | -4.72(-4.83–-4.61) | 13.47(9.46–20.17) | -42.67 | -3.69(-3.8–-3.58) |
| Cameroon | 0.81(0.45–1.25) | 77.28 | -1.52(-1.77–-1.26) | 32.63(18.03–53.86) | 77.11 | -1.31(-1.53–-1.09) |
| Canada | 0.05(0.04–0.08) | -71.2 | -5.89(-6.32–-5.45) | 7.88(6.15–10.14) | -36.62 | -3.26(-3.67–-2.86) |
| Central African Republic | 2.23(1.3–3.69) | 65.28 | -0.33(-0.45–-0.22) | 92.17(52.95–153.15) | 58.85 | -0.45(-0.55–-0.35) |
| Chad | 1.5(0.9–2.22) | 66.9 | -1.11(-1.4–-0.83) | 61.61(35.99–98.37) | 70.89 | -1.22(-1.5–-0.94) |
| Chile | 0.2(0.12–0.23) | -67.36 | -5.36(-6.19–-4.53) | 19.86(15.16–24.05) | -50.84 | -4.14(-4.9–-3.38) |
| China | 0.02(0.02–0.04) | -92.64 | -11.07(-11.96–-10.18) | 3.55(2.61–5.09) | -80.82 | -8.01(-8.97–-7.05) |
| Colombia | 0.19(0.14–0.32) | -75.75 | -6.01(-6.36–-5.66) | 17.26(13.37–23.89) | -67.76 | -5.03(-5.38–-4.67) |
| Comoros | 1.11(0.66–1.76) | 20.14 | -1.52(-1.73–-1.3) | 40.54(23.42–67.3) | -2.62 | -1.78(-2.1–-1.45) |
| Congo | 0.74(0.4–1.22) | 18.91 | -2.16(-2.35–-1.97) | 29.11(17.16–48.66) | 12.3 | -2.37(-2.57–-2.16) |
| Cook Islands | 0.01(0.01–0.03) | -71.76 | -5.39(-5.68–-5.11) | 3.43(2.35–5.03) | -32.43 | -1.75(-2.04–-1.46) |
| Costa Rica | 0.1(0.08–0.14) | 29.83 | -1.34(-1.85–-0.83) | 13.08(10.2–16.23) | 53.68 | -0.61(-0.89–-0.33) |
| Croatia | 0.08(0.06–0.11) | -87.89 | -8.99(-9.93–-8.04) | 7.45(5.73–9.32) | -82.07 | -7.77(-8.67–-6.87) |
| Cuba | 0.11(0.09–0.16) | -34.72 | -2.82(-3.01–-2.62) | 20.27(14.92–26.62) | -0.65 | -0.6(-0.89–-0.32) |
| Cyprus | 0.06(0.04–0.09) | -66.37 | -7.4(-8.08–-6.72) | 5.54(4.33–7.34) | -42.96 | -5.2(-5.68–-4.72) |
| Czechia | 0.07(0.05–0.09) | -48.04 | -2.95(-3.2–-2.7) | 6.19(4.9–7.64) | -37.65 | -2.38(-2.53–-2.23) |
| Côte d'Ivoire | 0.91(0.5–1.31) | 33.84 | -1.51(-1.71–-1.31) | 36.56(18.87–57.99) | 22.07 | -1.55(-1.73–-1.36) |
| DPR Korea | 0.1(0.04–0.24) | -54.3 | -3.5(-3.65–-3.35) | 8.23(4.62–15.95) | -50.63 | -2.99(-3.13–-2.84) |
| DR Congo | 1.05(0.58–2.09) | 55.47 | -0.76(-1.08–-0.44) | 42.28(24.5–85.48) | 42.77 | -0.92(-1.21–-0.62) |
| Denmark | 0.02(0.02–0.04) | -62.13 | -4.67(-5.23–-4.1) | 3.97(3.11–5.08) | -24.16 | -2.07(-2.33–-1.82) |
| Djibouti | 1.1(0.62–1.78) | 97.5 | -1.21(-1.51–-0.91) | 40.39(21.47–68.38) | 67.87 | -1.47(-1.81–-1.13) |
| Dominica | 0.45(0.28–0.65) | -20.3 | -1.28(-1.79–-0.77) | 36.34(26.14–47.89) | -12.43 | -0.81(-1.2–-0.42) |
| Dominican Republic | 1.28(0.77–2.01) | 98.62 | 2.04(0.83–3.26) | 86.12(59.21–123.06) | 88.58 | 1.82(0.73–2.92) |
| Ecuador | 0.27(0.18–0.54) | -50.63 | -4.89(-5.28–-4.49) | 23.35(17.4–38.06) | -38.47 | -4.01(-4.36–-3.67) |
| Egypt | 0.29(0.09–0.5) | -16.93 | -2.38(-2.48–-2.27) | 18.77(8.85–29.54) | -16.05 | -2.27(-2.37–-2.17) |
| El Salvador | 0.04(0.03–0.11) | -85.1 | -8.1(-8.83–-7.37) | 9.36(6.81–13.29) | -62.59 | -4.27(-4.93–-3.61) |
| Equatorial Guinea | 0.39(0.16–0.79) | -53.75 | -6.45(-6.92–-5.98) | 16.64(8.83–31.92) | -47.84 | -6.51(-7.01–-6) |
| Eritrea | 1.88(1.12–2.77) | 56.2 | -0.94(-1.03–-0.86) | 65.88(37.61–101.79) | 39.45 | -1.29(-1.38–-1.2) |
| Estonia | 0.06(0.04–0.09) | -84.89 | -7.25(-7.85–-6.64) | 5.9(4.56–7.89) | -76.35 | -5.74(-6.32–-5.16) |
| Eswatini | 0.75(0.34–1.41) | 36.89 | -0.57(-1.12–-0.01) | 38.07(17.51–70.39) | 34.4 | -0.41(-0.96–0.14) |
| Ethiopia | 1.82(1.19–2.49) | -6.56 | -2.73(-2.98–-2.48) | 66.03(42.86–89.68) | -10.38 | -2.83(-3.09–-2.56) |
| Fiji | 0.05(0.04–0.08) | -39.1 | -3.25(-3.5–-2.99) | 7.96(6.06–10.47) | -11.39 | -1.49(-1.7–-1.28) |
| Finland | 0.04(0.03–0.04) | -63.46 | -4.04(-4.36–-3.71) | 4.91(3.84–6.03) | -33.04 | -2.44(-2.7–-2.18) |
| France | 0.15(0.1–0.18) | -25.53 | -2.21(-2.36–-2.06) | 10.61(7.93–12.29) | -28.51 | -2.1(-2.2–-2.01) |
| Gabon | 0.54(0.23–1.08) | -1.69 | -1.83(-2.02–-1.65) | 22.08(11.67–43.52) | -4.51 | -1.99(-2.13–-1.84) |
| Gambia | 1.09(0.6–1.88) | 63.61 | -1.13(-1.3–-0.95) | 41.66(21.33–78.4) | 42.17 | -1.27(-1.48–-1.05) |
| Georgia | 0.81(0.47–1.01) | -20.34 | 1.18(0.62–1.74) | 45.66(28.01–56.01) | -27.61 | 0.94(0.4–1.49) |
| Germany | 0.03(0.03–0.04) | -49.89 | -3.41(-3.63–-3.18) | 4.18(3.3–5.32) | -21.27 | -1.52(-1.66–-1.38) |
| Ghana | 0.74(0.51–1.04) | 27.51 | -1.73(-1.86–-1.59) | 28.79(18.93–44.79) | 21.35 | -1.58(-1.7–-1.47) |
| Greece | 0.08(0.06–0.11) | -55.78 | -3.63(-4.52–-2.74) | 7(5.57–8.56) | -47.98 | -2.91(-3.57–-2.24) |
| Greenland | 0.77(0.41–1.11) | -62.28 | -4.1(-4.67–-3.53) | 46.42(28.01–66.4) | -64.52 | -4.1(-4.64–-3.55) |
| Grenada | 1.29(1.03–1.56) | -12.02 | -1.61(-2.15–-1.06) | 82.48(67.22–97.23) | -8.16 | -1.38(-1.86–-0.91) |
| Guam | 0.07(0.04–0.21) | -77.52 | -8.52(-9.11–-7.92) | 6.5(4.31–12.65) | -67.8 | -6.21(-6.72–-5.7) |
| Guatemala | 4.25(2.76–5.47) | 486.78 | 3.81(3.03–4.58) | 253.66(174.6–320.02) | 457.34 | 3.68(2.95–4.41) |
| Guinea | 1.4(0.81–2.09) | 22.22 | -0.84(-0.94–-0.74) | 59.42(32.12–101.42) | 16.26 | -0.96(-1.06–-0.87) |
| Guinea–Bissau | 1.55(0.98–2.22) | 0.65 | -1.52(-1.64–-1.4) | 60.86(38.05–91.52) | -7.41 | -1.72(-1.88–-1.56) |
| Guyana | 0.62(0.45–0.82) | -9.99 | -0.14(-0.71–0.43) | 43.23(32.93–54.98) | -7.09 | -0.02(-0.45–0.42) |
| Haiti | 5.56(1.62–13.8) | 32.17 | -1.28(-1.43–-1.12) | 325.68(124.29–718.47) | 26.77 | -1.26(-1.42–-1.1) |
| Honduras | 0.92(0.46–1.52) | -11.23 | -3.22(-3.31–-3.12) | 53.12(31.94–79.16) | -17.1 | -3.52(-3.66–-3.38) |
| Hungary | 0.02(0.02–0.03) | -69.28 | -4.47(-4.97–-3.97) | 4.01(3.07–5.07) | -42.27 | -2.27(-2.67–-1.86) |
| Iceland | 0.02(0.02–0.03) | -36.05 | -2.79(-2.94–-2.65) | 3.86(2.97–4.92) | 9.95 | -1(-1.12–-0.88) |
| India | 0.05(0.02–0.07) | -51.96 | -5.18(-5.54–-4.81) | 4.34(3.08–5.47) | -33.72 | -3.73(-3.97–-3.49) |
| Indonesia | 0.06(0.02–0.11) | -69.08 | -3.79(-4.06–-3.51) | 9.26(4.77–14.29) | -49.68 | -2.8(-3.1–-2.51) |
| Iran | 0.11(0.04–0.15) | 33.12 | 0.11(-0.74–0.98) | 10.08(6.17–12.89) | 27.26 | -0.2(-0.78–0.38) |
| Iraq | 3.46(2.18–5.14) | 62.29 | -1.79(-2.04–-1.55) | 179.34(116.37–265.2) | 49.64 | -1.93(-2.16–-1.7) |
| Ireland | 0.04(0.02–0.05) | -59.55 | -3.49(-4.34–-2.64) | 4.99(3.83–6.05) | -29.18 | -2(-2.56–-1.45) |
| Israel | 0.1(0.08–0.12) | -49.7 | -5.13(-5.83–-4.43) | 7.87(6.58–9.36) | -40.7 | -4.41(-4.97–-3.85) |
| Italy | 0.08(0.05–0.09) | -17.02 | -1(-1.26–-0.73) | 6.32(4.67–7.41) | -34.67 | -2.11(-2.33–-1.88) |
| Jamaica | 2.35(1.79–2.98) | 55.48 | -1.82(-3.27–-0.35) | 150.02(120.33–182.24) | 48.27 | -1.88(-3.11–-0.64) |
| Japan | 0.01(0.01–0.02) | -31.4 | -2.81(-2.92–-2.71) | 3.85(2.8–5.35) | 5.27 | -0.63(-0.8–-0.45) |
| Jordan | 0.08(0.04–0.12) | 20.12 | -3.79(-3.95–-3.63) | 9.13(6.36–11.8) | 64.03 | -2.42(-2.55–-2.29) |
| Kazakhstan | 0.29(0.21–0.37) | -40.91 | -3.02(-3.65–-2.39) | 16.79(12.65–20.82) | -42.92 | -3.03(-3.63–-2.42) |
| Kenya | 1.36(0.92–1.87) | 78.34 | -0.5(-0.77–-0.22) | 47.63(33.54–64.48) | 64.3 | -0.63(-0.89–-0.37) |
| Kiribati | 0.11(0.06–0.27) | 3.55 | -1.7(-1.84–-1.55) | 10.73(7.31–18.7) | 24.65 | -0.89(-0.95–-0.83) |
| Kuwait | 0.01(0.01–0.02) | -9.72 | -3.9(-4.26–-3.54) | 6.43(4.67–8.41) | 146.75 | -0.64(-0.82–-0.47) |
| Kyrgyzstan | 0.2(0.13–0.24) | 21.28 | -0.42(-0.82–-0.02) | 12.29(9.16–14.64) | 18.76 | -0.57(-0.87–-0.26) |
| Lao People's Democratic Republic | 0.12(0.05–0.28) | -57.46 | -5.06(-5.15–-4.96) | 12.84(8.2–21.48) | -40.36 | -3.8(-3.89–-3.7) |
| Latvia | 0.08(0.05–0.12) | -74.2 | -4.84(-5.27–-4.4) | 6.85(5.31–9.06) | -67.25 | -3.9(-4.27–-3.53) |
| Lebanon | 0.08(0.03–0.21) | -11.11 | -2.17(-2.39–-1.94) | 9.28(5.97–16.36) | 8.36 | -1.51(-1.72–-1.3) |
| Lesotho | 1.2(0.61–1.91) | 20.32 | 0.23(-0.12–0.58) | 58.8(30.23–93.29) | 19.33 | 0.32(-0.03–0.67) |
| Liberia | 1.05(0.59–2.09) | 7.24 | -2.28(-2.56–-2) | 40.72(21.96–85.45) | -5.42 | -2.65(-2.91–-2.39) |
| Libya | 0.11(0.05–0.3) | 36.83 | -1(-1.3–-0.69) | 10.07(6.52–19.67) | 35.85 | -0.99(-1.24–-0.74) |
| Lithuania | 0.08(0.06–0.11) | -80.1 | -5.48(-5.91–-5.05) | 7.28(5.66–9.33) | -73.14 | -4.56(-4.89–-4.23) |
| Luxembourg | 0.04(0.03–0.05) | -46.78 | -4.27(-4.48–-4.07) | 5.02(3.93–6.18) | -10.52 | -2.51(-2.72–-2.29) |
| Madagascar | 1.17(0.76–1.64) | 21.17 | -1.36(-1.42–-1.3) | 44.38(28.69–64.46) | 10.64 | -1.72(-1.82–-1.62) |
| Malawi | 1.06(0.7–1.48) | -0.78 | -1.81(-1.94–-1.69) | 39.53(25.01–59.21) | -13.47 | -2.18(-2.29–-2.07) |
| Malaysia | 0.05(0.02–0.07) | -19.82 | -3.57(-4.27–-2.86) | 8.01(5.85–10.27) | 22.96 | -1.87(-2.17–-1.56) |
| Maldives | 0.04(0.02–0.05) | -64.72 | -7.29(-7.72–-6.86) | 7.9(5.71–10.24) | -17.38 | -4(-4.37–-3.63) |
| Mali | 1.15(0.68–1.87) | 32.24 | -1.92(-2.04–-1.79) | 48.83(27.66–85.3) | 31.33 | -2.06(-2.25–-1.87) |
| Malta | 0.13(0.08–0.16) | -34.35 | -2.34(-2.44–-2.25) | 10(7.09–11.81) | -23.63 | -1.69(-1.78–-1.59) |
| Marshall Islands | 0.39(0.17–0.67) | -43.69 | -3.43(-3.55–-3.31) | 23.29(13.2–37.51) | -41.51 | -3.06(-3.18–-2.95) |
| Mauritania | 0.58(0.32–1.01) | -22.21 | -2.84(-2.93–-2.75) | 21.89(11.84–41.51) | -27.08 | -2.81(-2.92–-2.71) |
| Mauritius | 0.02(0.02–0.03) | -61.99 | -4.78(-5.48–-4.07) | 4.62(3.33–6.47) | -5.94 | -1.52(-1.86–-1.17) |
| Mexico | 0.53(0.43–0.65) | -58.73 | -4.33(-5.28–-3.37) | 32.45(26.83–39.02) | -59.42 | -4.11(-4.98–-3.23) |
| Micronesia | 0.33(0.17–0.55) | -62.03 | -3.93(-4.16–-3.7) | 19.52(11.79–30.19) | -61.18 | -3.59(-3.78–-3.39) |
| Monaco | 0.04(0.03–0.06) | -31.91 | -2.07(-2.18–-1.95) | 3.99(2.92–5.55) | -7.18 | -1.3(-1.41–-1.18) |
| Mongolia | 0.05(0.03–0.09) | -18.16 | -2.66(-3.19–-2.13) | 5.38(3.92–7.64) | -1.53 | -1.94(-2.19–-1.7) |
| Montenegro | 0.06(0.04–0.12) | -40.49 | -2.57(-3.04–-2.11) | 5.9(4.27–8.69) | -30.77 | -1.95(-2.27–-1.64) |
| Morocco | 1.09(0.31–2.78) | -32.68 | -3(-3.18–-2.83) | 60.15(23.08–139.39) | -37.62 | -3.07(-3.29–-2.85) |
| Mozambique | 1.28(0.81–1.77) | 22.35 | -1.36(-1.45–-1.26) | 47.53(28.3–71.02) | 16.83 | -1.53(-1.67–-1.38) |
| Myanmar | 0.2(0.08–0.44) | -75.1 | -6.23(-6.52–-5.95) | 18.41(10.92–36.41) | -67.75 | -5.18(-5.41–-4.95) |
| Namibia | 0.48(0.21–0.82) | 8.97 | -2.35(-2.85–-1.85) | 24.09(11.35–40.89) | 10.64 | -2.15(-2.64–-1.64) |
| Nauru | 0.24(0.12–0.38) | -54.93 | -3.04(-3.93–-2.14) | 14.32(8.28–22.66) | -50.47 | -2.6(-3.33–-1.86) |
| Nepal | 0.14(0.02–0.25) | -31.67 | -2.64(-2.93–-2.36) | 6.95(2.85–11.97) | -33.97 | -2.76(-3.1–-2.43) |
| Netherlands | 0.01(0.01–0.02) | -55.19 | -4.15(-4.44–-3.85) | 4.17(3.13–5.42) | 5.98 | -1.17(-1.41–-0.93) |
| New Zealand | 0.06(0.05–0.08) | -67.39 | -4.39(-5.09–-3.69) | 5.25(4.29–6.51) | -58.58 | -3.66(-4.29–-3.02) |
| Nicaragua | 0.3(0.21–0.45) | -41.9 | -4.45(-4.66–-4.24) | 23.28(17.64–31.12) | -37.87 | -4.01(-4.19–-3.83) |
| Niger | 1.1(0.61–1.77) | 57.28 | -1.55(-1.64–-1.46) | 44.63(23.9–77.74) | 46.95 | -1.88(-2.02–-1.73) |
| Nigeria | 0.83(0.53–1.19) | 9.37 | -2.3(-2.49–-2.1) | 33.91(21.09–48.99) | 8.43 | -2.64(-2.86–-2.41) |
| Niue | 0.12(0.05–0.25) | -74.05 | -4.59(-4.84–-4.35) | 8.39(4.58–15.68) | -69.88 | -3.69(-3.92–-3.46) |
| North Macedonia | 0.08(0.05–0.14) | -54.45 | -3.81(-4.47–-3.13) | 7.13(5.36–10.51) | -44.63 | -3(-3.48–-2.51) |
| Northern Mariana Islands | 0.29(0.19–0.6) | -71.23 | -5.45(-5.91–-4.99) | 14.92(10.05–27.63) | -74.15 | -4.88(-5.23–-4.53) |
| Norway | 0.02(0.02–0.03) | -69.6 | -5.41(-5.93–-4.87) | 3.26(2.45–4.53) | -35.68 | -2.83(-3.25–-2.4) |
| Oman | 0.01(0.01–0.03) | -49.05 | -5.33(-5.73–-4.92) | 6.08(4.34–8.19) | 63.65 | -1.5(-1.86–-1.15) |
| Pakistan | 0.29(0.17–0.4) | 40.56 | -1.06(-1.19–-0.93) | 15.18(9.17–21.51) | 40.04 | -1.02(-1.13–-0.91) |
| Palau | 0.19(0.1–0.3) | -30.4 | -2.44(-2.53–-2.34) | 11.39(7.21–16.72) | -32.64 | -1.92(-2.04–-1.79) |
| Palestine | 0.61(0.05–0.96) | 5.38 | -2.39(-3.57–-1.2) | 40.6(7.67–61.16) | 5.49 | -2.25(-3.31–-1.17) |
| Panama | 0.08(0.05–0.11) | -50.85 | -5.03(-5.55–-4.52) | 11.94(9.21–15.08) | -10.22 | -2.78(-3.12–-2.44) |
| Papua New Guinea | 0.24(0.11–0.61) | 91.57 | -0.49(-0.77–-0.22) | 21.33(12.02–48.93) | 99.16 | -0.37(-0.6–-0.14) |
| Paraguay | 0.36(0.23–0.58) | -14.53 | -3.18(-3.5–-2.85) | 25.09(18.12–36.95) | -15.28 | -3.02(-3.3–-2.74) |
| Peru | 0.93(0.56–1.33) | -11.68 | -2.24(-2.55–-1.93) | 62.13(42.54–83.63) | -11.6 | -2.08(-2.34–-1.81) |
| Philippines | 0.12(0.05–0.17) | 51.87 | -0.38(-0.84–0.08) | 14.06(9.92–17.78) | 95.92 | 0.6(0.37–0.82) |
| Poland | 0.04(0.03–0.05) | -59.73 | -3.97(-4.34–-3.6) | 5.2(4.07–6.45) | -37.73 | -2.49(-2.85–-2.12) |
| Portugal | 0.06(0.05–0.09) | -78.63 | -7.08(-7.49–-6.66) | 5.33(4.32–6.9) | -70.17 | -5.57(-5.93–-5.21) |
| Puerto Rico | 0.44(0.34–0.56) | 21.73 | 0.32(-0.75–1.4) | 32.79(26.14–40.59) | 20.6 | 0.29(-0.62–1.2) |
| Qatar | 0.07(0.04–0.12) | 54.63 | -5.56(-5.85–-5.28) | 9.33(6.69–12.65) | 174.65 | -3.41(-3.73–-3.09) |
| Republic of Korea | 0.01(0.01–0.02) | -79.06 | -6.45(-7.05–-5.86) | 4.65(3.41–6.1) | -15.22 | -1.77(-2.25–-1.28) |
| Republic of Moldova | 0.08(0.04–0.1) | -81.18 | -6.16(-6.83–-5.49) | 6.92(5.01–8.37) | -74.48 | -5.17(-5.79–-4.55) |
| Romania | 0.04(0.02–0.05) | -73.88 | -4.52(-4.96–-4.07) | 4.95(3.64–6.15) | -55.99 | -2.99(-3.31–-2.67) |
| Russian Federation | 0.18(0.13–0.23) | -55.74 | -4.09(-4.66–-3.5) | 12.15(9.65–15.02) | -57.3 | -4.08(-4.56–-3.6) |
| Rwanda | 0.87(0.59–1.22) | -19.22 | -3.23(-3.52–-2.94) | 33.01(20.82–50.14) | -25.76 | -3.45(-3.73–-3.18) |
| Saint Kitts and Nevis | 2.97(1.8–4.06) | 12.15 | -1.1(-1.46–-0.74) | 160.44(100.04–216.67) | 6.53 | -0.78(-1.16–-0.39) |
| Saint Lucia | 1.21(0.97–1.47) | 54.62 | -0.04(-0.54–0.47) | 75.56(62.15–90.72) | 43.97 | 0.02(-0.42–0.47) |
| Saint Vincent and the Grenadines | 1.4(1.15–1.68) | 4.6 | -1.11(-1.76–-0.47) | 87.69(72.5–102.91) | -1.33 | -1.06(-1.65–-0.46) |
| Samoa | 0.18(0.1–0.29) | -54.48 | -3.88(-4.12–-3.65) | 12.91(8.68–18.84) | -46.58 | -3.15(-3.36–-2.95) |
| San Marino | 0.04(0.02–0.06) | 19.21 | -0.79(-0.99–-0.6) | 3.71(2.57–5.17) | 34.16 | -0.31(-0.43–-0.19) |
| Sao Tome and Principe | 0.59(0.32–1.03) | -20.61 | -1.97(-2.14–-1.79) | 26.6(14.39–51.87) | -32.33 | -2.45(-2.67–-2.24) |
| Saudi Arabia | 0.82(0.44–1.4) | 1.37 | -3.95(-4.04–-3.86) | 46.15(26.62–75.49) | -0.91 | -3.7(-3.78–-3.62) |
| Senegal | 0.93(0.59–1.35) | 16.18 | -1.33(-1.49–-1.16) | 36.04(21.9–57.85) | -0.74 | -1.53(-1.74–-1.31) |
| Serbia | 0.12(0.07–0.17) | -59.88 | -3.8(-4.08–-3.51) | 8.63(6.22–11.49) | -58.11 | -3.52(-3.75–-3.29) |
| Seychelles | 0.04(0.01–0.1) | -68.7 | -5.31(-6.01–-4.61) | 6.02(3.86–9.48) | -36.18 | -2.92(-3.41–-2.43) |
| Sierra Leone | 1.24(0.68–2.04) | 38.1 | -1.01(-1.21–-0.81) | 54.91(27.85–105.53) | 34.49 | -1.1(-1.27–-0.93) |
| Singapore | 0.01(0.01–0.02) | -23.17 | -3.49(-3.79–-3.18) | 4.34(3.23–5.67) | 79.11 | -0.79(-0.96–-0.61) |
| Slovakia | 0.16(0.08–0.24) | -50.57 | -2.94(-3.29–-2.59) | 11.09(6.89–15.25) | -47.16 | -2.66(-2.96–-2.37) |
| Slovenia | 0.02(0.01–0.04) | -77.2 | -6.29(-6.75–-5.83) | 3.75(2.81–5.05) | -39.94 | -3.22(-3.67–-2.77) |
| Solomon Islands | 0.56(0.3–0.87) | 32.64 | -1.03(-1.13–-0.93) | 38.13(20.09–59.93) | 29.7 | -1.01(-1.1–-0.92) |
| Somalia | 3.41(1.51–6.4) | 163.27 | 0.02(-0.07–0.1) | 130.54(56.33–250.76) | 160.6 | -0.1(-0.2–0.01) |
| South Africa | 0.46(0.3–0.66) | -8.07 | -2.61(-3.15–-2.07) | 25.16(17.35–34.96) | -10.78 | -2.64(-3.18–-2.09) |
| South Sudan | 1.13(0.47–1.98) | 1.57 | -1.16(-1.23–-1.09) | 43.51(17.82–81.19) | -3.89 | -1.35(-1.46–-1.23) |
| Spain | 0.05(0.04–0.06) | -78.42 | -6.15(-6.78–-5.52) | 5.24(4.17–6.42) | -65.16 | -4.5(-5.11–-3.9) |
| Sri Lanka | 0.33(0.13–0.52) | -66.15 | -5.52(-6.18–-4.85) | 21.18(11.45–30.79) | -64.63 | -5.49(-6.15–-4.82) |
| Sudan | 0.35(0.12–0.72) | -11.21 | -2.4(-2.63–-2.17) | 21.06(8.81–41.39) | -12.75 | -2.28(-2.5–-2.07) |
| Suriname | 0.74(0.46–1.01) | 43.46 | -0.43(-1–0.14) | 51.71(36.52–67.16) | 41.36 | -0.39(-0.84–0.07) |
| Sweden | 0.03(0.02–0.03) | -49.14 | -2.84(-3.05–-2.64) | 3.59(2.71–4.97) | -14.9 | -1.34(-1.47–-1.2) |
| Switzerland | 0.04(0.03–0.05) | -84.34 | -8.01(-8.46–-7.55) | 4.47(3.59–5.65) | -68.81 | -5.9(-6.52–-5.29) |
| Syrian Arab Republic | 0.11(0.06–0.19) | -77.46 | -6.41(-7.13–-5.68) | 11.04(7.64–15.64) | -71.64 | -5.25(-5.96–-4.54) |
| Taiwan  (Province of China) | 0.02(0.02–0.03) | -81.66 | -6.38(-6.99–-5.77) | 3.64(2.76–4.71) | -55.04 | -3.38(-3.98–-2.78) |
| Tajikistan | 0.24(0.08–0.35) | 88.54 | -1.12(-1.46–-0.78) | 14.12(6.83–19.74) | 83.12 | -1.17(-1.47–-0.86) |
| Thailand | 0.1(0.06–0.17) | -76.67 | -6.9(-7.69–-6.11) | 10.94(8.36–14.91) | -64.59 | -5.37(-5.9–-4.83) |
| Timor–Leste | 0.11(0.05–0.2) | -59.64 | -5.15(-5.63–-4.67) | 12.27(8.67–17.03) | -41.7 | -3.58(-3.93–-3.24) |
| Togo | 0.97(0.59–1.55) | 45.68 | -1.32(-1.48–-1.16) | 37.84(22.02–63.69) | 27.74 | -1.42(-1.56–-1.28) |
| Tokelau | 0.11(0.05–0.2) | -78.16 | -4.92(-5.09–-4.76) | 7.73(4.59–12.54) | -73.53 | -4.14(-4.29–-3.98) |
| Tonga | 0.07(0.04–0.12) | -49.52 | -3(-3.16–-2.85) | 8.4(6.13–11.15) | -28.92 | -1.53(-1.65–-1.4) |
| Trinidad and Tobago | 0.19(0.13–0.26) | -20.48 | -0.52(-1.35–0.32) | 23.04(17.82–28.55) | 6.76 | -0.08(-0.56–0.4) |
| Tunisia | 0.31(0.11–0.72) | -29.02 | -2.48(-2.61–-2.35) | 20.28(9.68–43.79) | -30.71 | -2.28(-2.43–-2.13) |
| Turkey | 0.77(0.38–1.05) | -11.84 | -1.74(-2.11–-1.37) | 47.38(25.92–62.95) | -11.21 | -1.29(-1.7–-0.88) |
| Turkmenistan | 0.35(0.2–0.55) | 99.41 | 1.09(0.66–1.51) | 22.14(13.89–32.31) | 74.61 | 0.85(0.5–1.19) |
| Tuvalu | 0.27(0.11–0.56) | -62.43 | -4.23(-4.46–-4.01) | 15.29(7.64–29.65) | -62.91 | -4.1(-4.31–-3.88) |
| Uganda | 0.88(0.59–1.22) | 21.28 | -2.32(-2.52–-2.12) | 33.09(21.04–50.93) | 18.26 | -2.36(-2.57–-2.14) |
| Ukraine | 0.24(0.17–0.34) | -23.71 | -1.41(-2.15–-0.66) | 16.28(12.64–22.62) | -23.47 | -1.14(-1.79–-0.49) |
| United Arab Emirates | 0.12(0.03–0.48) | 350.45 | -0.74(-1.02–-0.46) | 11.53(6.42–29.17) | 353.68 | -0.58(-0.82–-0.34) |
| United Kingdom | 0.02(0.02–0.03) | -40.82 | -2.46(-2.65–-2.27) | 3.96(3.08–5.03) | -2.72 | -0.82(-0.97–-0.66) |
| United Republic of Tanzania | 0.89(0.58–1.23) | 26.58 | -1.53(-1.59–-1.47) | 34.86(21.39–54.45) | 20.02 | -1.54(-1.63–-1.45) |
| United States of America | 0.2(0.17–0.24) | -58.67 | -4.33(-4.68–-3.98) | 14.2(12.43–16.97) | -58.5 | -4.4(-4.87–-3.92) |
| United States Virgin Islands | 0.62(0.38–0.87) | -19.12 | -1.32(-1.97–-0.67) | 36.49(25.06–50.28) | -28.23 | -1.65(-2.33–-0.96) |
| Uruguay | 2.07(1.91–2.24) | -56.07 | -3.81(-4.16–-3.46) | 125.49(113.03–139.29) | -57.9 | -3.82(-4.16–-3.48) |
| Uzbekistan | 0.04(0.03–0.05) | 55.88 | -0.16(-0.36–0.03) | 5.04(3.95–6.26) | 65.74 | -0.28(-0.42–-0.13) |
| Vanuatu | 0.45(0.2–0.78) | 4.3 | -2.88(-3.06–-2.71) | 26.07(14.78–41.47) | 6.82 | -2.53(-2.68–-2.39) |
| Venezuela | 1.99(1.49–2.59) | 77.76 | 1.31(0.84–1.78) | 137.08(105.64–172.02) | 67.42 | 1.18(0.75–1.6) |
| Viet Nam | 0.23(0.09–0.41) | -58.77 | -5.77(-6.27–-5.26) | 17.67(9.95–26.81) | -52.57 | -4.96(-5.38–-4.54) |
| Yemen | 0.51(0.13–1.28) | 87.48 | -1.34(-1.62–-1.06) | 30.71(10.44–69.02) | 76.46 | -1.23(-1.48–-0.98) |
| Zambia | 1.1(0.75–1.48) | 17.13 | -2.17(-2.48–-1.87) | 40.68(27.26–55.61) | 4.93 | -2.43(-2.74–-2.12) |
| Zimbabwe | 0.39(0.19–0.73) | 107.86 | 1.24(0.76–1.71) | 18.74(9.65–35.33) | 108.12 | 1.44(0.99–1.89) |

DALYs: disability adjusted life years; EAPC: estimated annual percentage change; ASR, age-standardized rate; CI, confidence interval; UI: uncertainty interval.
